# Nonsense-mediated mRNA decay factors target short poly(A)-tailed mRNAs lacking a premature termination codon

**DOI:** 10.1038/s41467-026-72132-1

**Published:** 2026-04-17

**Authors:** Toni Gouhier, Cosmin Saveanu, Gwenael Badis

**Affiliations:** 1 Institut de Biologie de l’Ecole Normale Supérieure (IBENS), Ecole Normale Supérieure, CNRS UMR8197, INSERM, PSL Research University, Paris, France; 2 Institut Pasteur, Université Paris Cité, CNRS UMR3525, Genetics of Macromolecular Interactions, Paris, France; 3 Sorbonne Université, Collège doctoral, Paris, France; 4 Institut Pasteur, Université Paris Cité, RNA Biology of Fungal Pathogens, Paris, France

**Keywords:** RNA quality control, Molecular biology, RNA decay

## Abstract

Nonsense-mediated mRNA decay (NMD) is a conserved eukaryotic surveillance pathway known to degrade mRNAs containing premature termination codons (PTCs). mRNA recognition by the Upf1, Upf2 and Upf3 NMD factors is favoured by long distances between the stop codon and the poly(A)-binding protein (Pab1) binding site. Using Nanopore direct RNA sequencing, we now show that PTC-containing NMD targets account for only 6% of Upf1-associated RNA and have long poly(A) tails, indicating that Upf1-binding occurs prior to their deadenylation. Conversely, most Upf1-associated mRNAs have short poly(A) tails, lack a PTC and correspond to highly expressed genes. A short poly(A) tail is thus an important feature of a new class of Upf1 targets, redefining the scope of the NMD RNA degradation pathway. Recognition of short poly(A)-tailed mRNAs by Upf1, Upf2 and Upf3 (the NMD machinery) triggers their decapping, uncovering a hitherto unknown role of the NMD machinery in the degradation of these transcripts.

## Abstract (web summary)

Nonsense-mediated mRNA decay (NMD) affects mRNAs with premature termination codons (PTC). Using Direct RNA sequencing, the authors identified thousands of short poly(A)-tailed transcripts specifically bound and destabilized by the NMD machinery and representing a new class of targets.

## Introduction

Nonsense-mediated mRNA decay (NMD) is a major cytoplasmic surveillance pathway, highly conserved across eukaryotes, that eliminates aberrant transcripts containing a premature termination codon (PTC) to prevent the production of truncated, potentially deleterious proteins. Upstream frameshift 1 (Upf1), the NMD core factor, is an ATP-dependent RNA helicase. In yeast, Upf1 associates with at least two distinct subcomplexes: the Upf12/3 complex, comprising Upf1, Upf2, and Upf3, and the Upf1-decapping complex, which includes the decapping enzyme Dcp1, Dcp2, Edc3, Ebs1 and Nmd4^1^. The Upf1-2/3 complex plays a central role in NMD activation and interacts with the translation release factors eRF1 and eRF3, which are crucial for efficient ribosome release^2,3^. In contrast, the Upf1-decapping complex mediates mRNA decapping, triggering subsequent 5′ to 3′ exonucleolytic degradation by Xrn1. Remarkably, this process occurs independently of prior deadenylation^4–6^. According to the faux 3′UTR consensus model, NMD-targeted mRNAs experience defective translation termination due to the presence of a PTC or an unusually long 3′UTR^7–9^. This model posits that inefficient translation termination arise from the absence of the stimulatory role of a proximal Pab1 on eRF3^10^.

In contrast to NMD substrates, translation termination for “normal” and relatively stable non-PTC-containing mRNAs (noPTC) is facilitated by the interaction between eRF3 and the poly(A)-binding protein Pab1, recruited close to the stop codon by the proximal poly(A) tail^11^. In addition, noPTC mRNA decay uses a deadenylation-dependent mechanism, where shortening of the poly(A) tail is followed by decapping and degradation^12,13^. Deadenylation is mostly carried out by two enzymatic complexes, Pan2/Pan3 and CCR4/NOT, both contributing to the shortening of the poly(A) tail to a residual length of 10–15 nucleotides^14,15^. The shortened poly(A) tail is then recognized by the Lsm1-7/Pat1 complex, which was proposed to facilitate decapping through the recruitment of Dcp1/2. Once decapped, mRNAs are predominantly degraded from 5′ to 3′ by the Xrn1 exonuclease, or in a minor way, from 3′ to 5′ by the Ski7-exosome complex (see Coller and Parker^16^ for a review).

PTC-containing mRNAs are common and include mRNA with upstream open reading frames, retained introns, or frameshift mutations, as well as non-coding RNAs like XUTs and SUTs (see Nickless et al.^17^ for a review). Initially thought to be rare, transcripts with alternative transcription start sites that generate PTCs have been identified in more than half of yeast genes. These transcripts, although representing a minority of the transcriptome, are selectively targeted and degraded by NMD^18^. Notably, NMD also downregulates a substantial fraction of non-mutated mRNAs lacking canonical PTC features^19,20^.

Several strategies have been employed to assess the transcriptome-wide scope of mRNA degradation by NMD. An initial approach involved the identification of transcripts that were up-regulated or stabilized upon Upf1 inactivation^19,21,22^. These studies revealed the most efficiently degraded NMD substrates, which presumably correspond to homogeneous populations of PTC-containing mRNAs, estimated to represent no more than 5% of all mRNAs^19^. To identify Upf1 direct targets in mammals, RNA immunoprecipitation followed by sequencing (RIP-seq) and cross-linking-based methods led to different outcomes. These approaches identified Upf1 binding not only to PTC-containing mRNAs but also to a wide range of noPTC transcripts, suggesting a broader role for Upf1 in general mRNA decay^23–25^. NMD in yeast is different from that in mammals since, for example, Upf1 phosphorylation does not seem to be determinant for its binding to NMD-targeted transcripts.

The above-cited studies did not measure the poly(A) tails of individual Upf1-associated RNAs. Each transcript is a complex mix of RNAs at different stages of polyadenylation, in relation to their degradation rates. Recent studies established that deadenylation is not required for decapping and degradation of most yeast mRNAs under normal growth conditions^26,27^, or during meiosis^28^. Consistently, unstable mRNAs, which are rapidly decapped and degraded 5′ to 3′, tend to have long poly(A) tails, whereas stable mRNAs harbour shorter poly(A) tails^29,30^. At steady state, mRNAs expressed from a single gene is a mix of molecules with long (>50 to 80 adenosine residues in yeast, up to 300 in humans), intermediate (25–50 A), short (<25 A), or even lacking poly(A) tails. Transcripts can be capped or decapped and may undergo partial 5′ degradation. Since NMD substrates are unstable, they are degraded before having time to undergo deadenylation^27^. Thus, the status of the poly(A) tail in transcript populations provides important and previously unexplored information about their degradation and targeting by NMD.

On one hand, NMD is deadenylation-independent and targets mRNAs with long poly(A) tails^27^. On the other hand, the Lsm1-7/Pat1 complex is involved in the recognition of short polyadenylated mRNAs, facilitating decapping^31^. Surprisingly, Upf1, Lsm1, Pat1 and Xrn1 degradation factors can coexist in the same mRNP^1^, Since the recognition of short polyadenylated mRNAs by the Lsm1-7/Pat1 complex enhances decapping^31^, these biochemical data suggest that Upf1 may also play a role in the degradation of short poly(A)-tailed mRNAs as a decay factor beyond its established role in NMD.

Despite extensive efforts to understand NMD mechanisms, the rules governing transcript recognition by Upf1 and the NMD machinery remain unclear. This is particularly true for mRNAs that do not contain a PTC but are associated with Upf1. To address this point, we used a combination of high-throughput RNA and RIP sequencing in *Saccharomyces cerevisiæ*, to show that Upf1 associates not only with PTC-containing NMD substrates with long poly(A) tails but also with a substantial fraction of transcripts lacking a PTC and carrying short poly(A) tails. This RNA subpopulation is stabilized in the absence of a functional NMD machinery. Our results show that this subfraction of short poly(A)-tailed mRNAs, originating from stable and highly translated mRNAs, undergoes NMD-dependent degradation, revealing a novel role for the NMD machinery in the degradation of mRNAs beyond PTC-containing transcripts.

## Results

### A subpopulation of mRNAs lacking PTCs is associated with Upf1

**Fig. 1:**
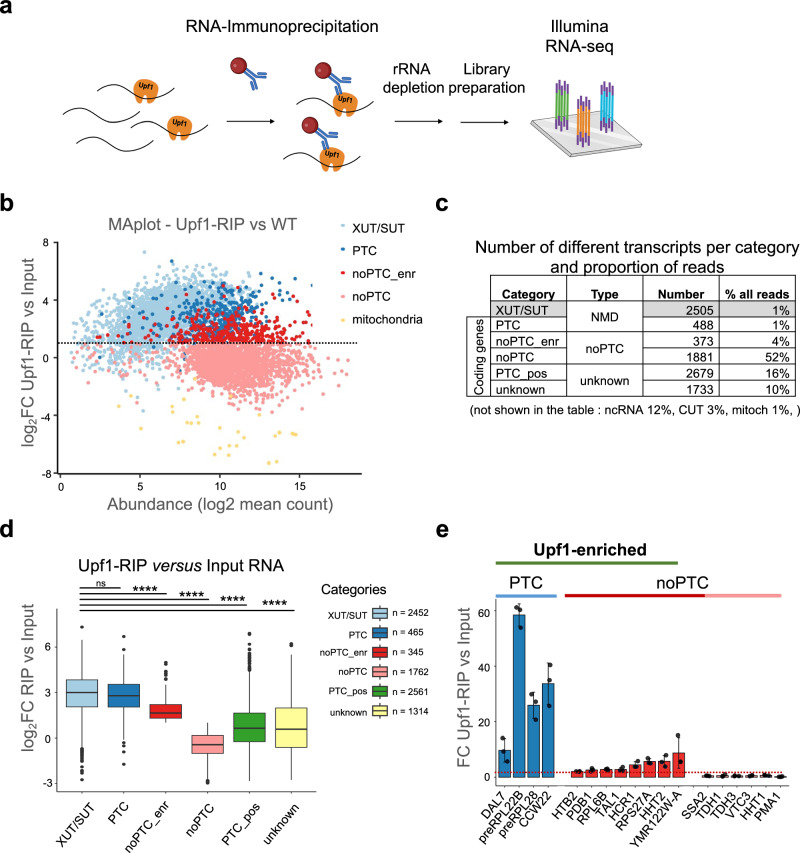
A subpopulation of mRNAs lacking PTCs is associated with Upf1. **a** Schematic representation of RNA-Immunoprecipitation (RIP) followed by Illumina sequencing. Created in BioRender. Le Hir, H. (2026) https://BioRender.com/k6q67wg^63^. **b** MAplot rep resenting the log2 of fold change (log2FC) between Upf1-RIP and WT (log2FC=log2(Upf1-RIP)-log2(WT)) as a function of transcript abundance (A = log2 ((Upf1-RIP mean count + WT mean count)/2) from Illumina sequencing. Each transcript type is represented as follow: XUT/SUT (light blue), PTC (dark blue), noPTC-Upf1enr (red), noPTC (pink), and mitochondrial transcripts as a control (yellow). Dotted lines indicate a two-fold threshold (log2FC = 1). NoPTC_enr were defined as noPTC transcripts enriched more than two-fold in Upf1 RIP (log2FC Upf1 RIP vs input>1). Three biological replicates of each condition were used for all the experiments of this study. **c** Number of transcripts of each type in the different categories. Grey-shaded boxes represent XUT/SUT transcripts, and white boxes detail protein-coding gene sub-categories (PTC, noPTC, PTC_pos, and unknown). **d** Boxplots comparing Upf1-RIP vs Input log2FC for the mentioned categories of transcripts (from three biological replicates). The cartoon describes categories (as in (**a**) and corresponding number of detected transcripts. Pvalues (Tukey HSD test): ns = *p* > 0.05, * = *p* < 0.05, ** = *p* < 0.01, *** = *p* < 0.001 and **** = *p* < 0.0001. See also Supplementary Table 2 for detailed p-values and Supplementary Data 2 for normalized counts. **e** Barplot comparing Upf1-RIP versus Input fold change (FC) for PTC and noPTC examples. Data are presented as mean values +/− SD from three biological replicates. See also Supplementary Fig. 1, and Supplementary Data 2 for normalized counts.

Because transcript binding to Upf1 is the first step in NMD activation, Upf1-TAP-associated RNAs were purified and sequenced by Illumina (RIPseq) (Fig. 1a). RIPseq was performed under conditions that have been previously used to describe the architecture of yeast NMD complexes^1^. We found a robust association of known NMD-targeted transcripts (**XUT/SUT** and **PTC**-containing mRNAs) with Upf1 (light and dark blue dots, Fig. 1b). We distinguished non-PTC-containing transcripts according to their enrichment in Upf1 immunoprecipitates (**no_PTC_enr** in red and **noPTC**, pink). Abundant mitochondrial-encoded mRNAs (yellow) were hardly detected, indicating a low immunoprecipitation background in these experiments.

As a first step in understanding the rules that govern transcript association with Upf1, we classified annotated mRNAs into categories based on previously obtained results (Fig. 1c). **XUT/SUT** RNAs are typical PTC-containing NMD targets, as they have random short open reading frames followed by long untranslated regions^18^. The NMD targets group also comprises genuine **PTC**-containing mRNAs^19,32^. Some studies in yeast define NMD targets as mRNAs stabilized more than two-fold upon Upf1 deletion^18,21,32–34^. This parameter was thus also considered to define the PTC category (Supplementary Fig. 1a). Genes that express mRNAs without an obvious PTC were annotated **noPTC**, provided they express less than 10% of NMD-sensitive 5′ transcript isoforms and their major mRNA isoform is not increased upon Upf1 deletion. Genes expressing more than 10% of NMD-sensitive 5′ transcript isoforms were annotated as generating possible PTC-containing mRNA (**PTC_pos**). The remaining transcripts were considered to have an “**unknown**” NMD status. Genes corresponding to each category are individually listed in Supplementary Data 1.

The RIPseq data were classified according to the categories defined in Fig. 1c. A total of 2452 detected transcripts corresponded to **XUT/SUT**, and 465 to **PTC**. A reasonable assumption is that the mitochondrial signal reflects the background level of mRNA detection by Upf1. In these immunoprecipitations, almost all the transcripts identified in the Upf1 RIP experiment are above the mitochondrial background. We can thus conclude that Upf1 is found associated with many abundant mRNAs that are not NMD sensitive (**noPTC** categories, red and pink dots in Fig. 1b). We distinguished 345 mRNAs from this category that are particularly enriched with Upf1 (log2FC RIP versus Input >1, **noPTC_enr**), whereas 1762 were not (**noPTC** – Fig. 1d). In addition, the  noPTC_enr transcripts, were not stabilized upon Upf1 deletion, similar to other noPTC mRNAs (Supplementary Fig. 1b). Considering the abundance of the noPTC transcripts, we counted that the non NMD-sensitive categories, composed of noPTC transcripts enriched or not in the RIP with Upf1, represented 56% (52% + 4%) of all the reads in the Illumina sequencing dataset, while NMD-sensitive transcripts, composed of PTC and XUT/SUT represented only 2% (Fig. 1c), although they were strongly enriched in Upf1 immunoprecipitates in comparison with their relative levels in the total extracts, as illustrated in Fig. 1e. Among the noPTC representative examples, some, such as *HHT2* or *RPS27A* were enriched, though to lower levels than the PTC ones.

The stability of mRNAs depends on their poly(A) and translation status^35^. The noPTC categories, whether enriched or not with Upf1, displayed relatively longer half-lives^36^, when compared with mRNAs of the PTC, PTC_pos and unknown categories (Supplementary Fig. 1c).

Thus, in addition to PTC-NMD targets, Upf1 is also bound to mRNAs that do not contain a PTC (noPTC) and for which no link with NMD has been previously established.

We further analysed the features of noPTC and noPTC_enr mRNAs. Notably, despite no stabilization upon *UPF1* deletion, noPTC_enr mRNAs harboured shorter coding sequences and slightly longer 3′UTRs (Supplementary Fig. 1d, e). Since codon usage correlates with mRNA abundance and stability^37^, we examined the features of PTC, no_PTC_enr and no_PTC coding sequences. Relative codon usage, in comparison with all coding sequences from yeast, showed specific patterns (Supplementary Fig. 2a). Consistently, coding sequences for PTC containing transcripts were enriched in destabilizing codons, as estimated by the negative correlation between relative codon usage and the codon stability coefficient (Supplementary Fig. 2b). On the contrary, the noPTC population consisted mostly of relatively abundant mRNAs with a codon usage that was biased accordingly (Supplementary Fig. 2c). Finally, the noPTC_enr population showed a positive correlation between its codon usage and codon stability coefficients, with the notable exception of the GGG and GGC glycine codons, which were over-represented (Supplementary Fig. 2d). Altogether, codon usage for the no_PTC_enr mRNA population was globally similar with the codon usage of no_PTC mRNAs that were not obviously enriched in the Upf1 bound fraction, and differed from the codon usage of PTC mRNAs.

### Upf1 binds to the 3′UTR of noPTC_enr transcripts, but also to noPTC mRNAs

**Fig. 2:**
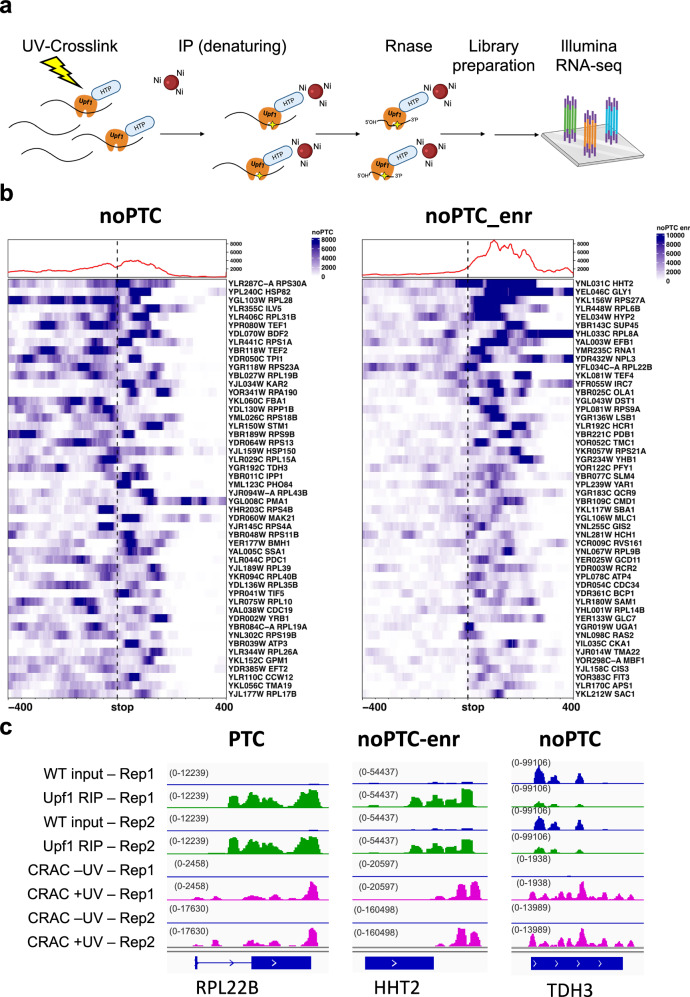
Cross-linking Analysis of cDNA sequencing (CRAC) with Upf1. **a** Schematic representation of Cross-linking Analysis of cDNA sequencing (CRAC) with Upf1. Created in BioRender. Le Hir, H. (2026) https://BioRender.com/r5e7t90^64^. **b** Heatmap representation of the CRAC signal centred on the Stop codon of the strongest signal, top 50 hits of the noPTC (left) and noPTC_enr (right) categories. **c** IGV profiles of RNAseq Illumina from WT (blue) and Upf1 RIP (green) experiments and CRAC signal (WT in blue, CRAC in pink) for three representative transcripts: *RPL22B* for PTC category, *HHT2* for noPTC_enr, and *TDH3* for noPTC category. CRAC – UV represents the signal in the controls without UV crosslinking. See also Supplementary Fig. 2.

To better understand how the signal obtained by co-purification of RNA with Upf1 correlates with the protein binding to specific sites on transcripts, we performed a cross-linking experiment, also known as CRAC (ref. ^38^, schematics in Fig. 2a). The distribution of signal across transcripts was different for the noPTC and noPTC_enr categories, as illustrated by the heatmaps presented in Fig. 2b. Top 50 transcripts (in terms of number of reads) were selected for each category, and the signal upstream and downstream the stop codon was visualized. Upf1 binding was concentrated in the 3′ UTR region of noPTC_enr transcripts and distributed more evenly on both sides of the stop codon region for noPTC transcripts (Fig. 2b). Examples of signal distribution illustrating the same trend for individual transcripts representative of PTC (*RPL22B* pre-mRNA), noPTC_enr (*HHT2*) and no_PTC (*TDH3*) categories and for two independent biological replicates are shown in Fig. 2c. The presented examples also illustrate the specificity of the CRAC data by showing that, in the absence of UV crosslinking, the signal was practically absent.

Similar to the PTC-containing *RPL22B* pre-mRNA, the Upf1 signal was present predominantly in the 3′UTR region of the noPTC_enr *HHT2*, but was distributed along the whole transcript region for the noPTC *TDH3*. However, compared with the control experiments that omit the UV crosslinking step, we observed an obvious signal of Upf1 association within the body of *TDH3*.

Thus, unexpectedly, CRAC experiments revealed that Upf1 associates with specific transcripts lacking PTCs, whether or not they were well-enriched in the RIP-seq data. However, the Upf1 strong signal, specifically in the 3′ UTR of the noPTC_enr transcript, reveals an additional shade of binding specificity. This observation is consistent with previous studies in mammalian cells, where Upf1 was shown to bind broadly to mRNAs, with a marked enrichment in 3′ UTRs presumably due to translation-dependent displacement from coding sequences by elongating ribosomes^23,24^. Since enrichment is defined on the basis of the relative comparison between the mRNA population in the Upf1-bound fraction and total mRNA, a possible explanation of these results is that Upf1 might bind only to a specific fraction of the mRNA for a given gene. Even if a fraction of a transcript is tightly bound to Upf1, it cannot appear enriched if this fraction is minor.

### Nanopore Direct RNA sequencing redefines Upf1-bound mRNAs landscape

**Fig. 3:**
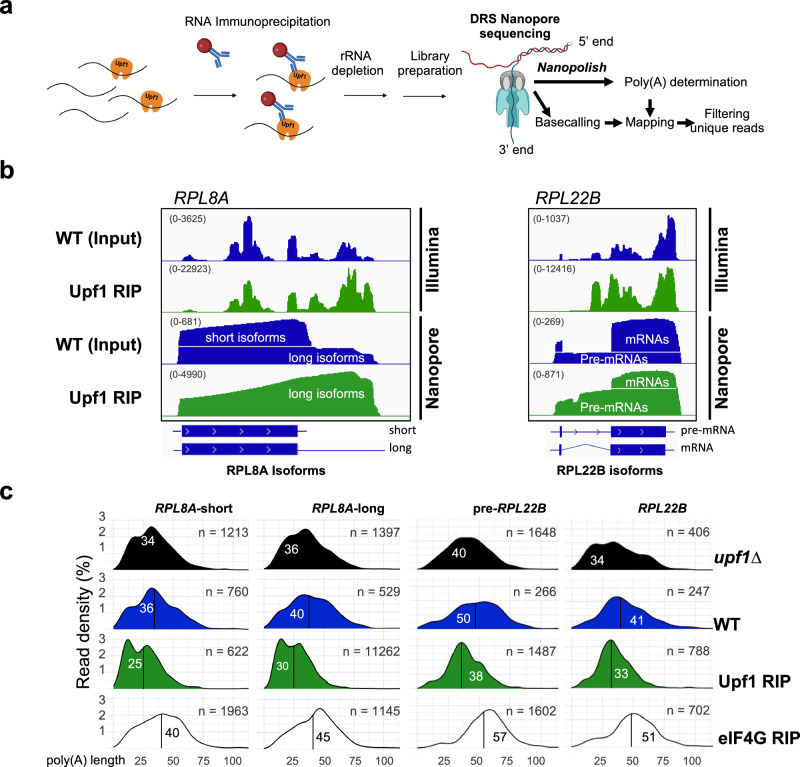
Nanopore Direct RNA sequencing redefines Upf1-bound mRNAs landscape. **a** Schematic representation of RNA-Immunoprecipitation (RIP) followed by Nanopore sequencing. Created in BioRender. Le Hir, H. (2026) https://BioRender.com/xx8gqqp^65^. **b** Signal intensity of RNA associated with Upf1-TAP (green) compared to total RNA from WT (blue) for Illumina or Nanopore sequencing (with Autoscale option in the Integrated Genome Viewer, IGV). Two examples are shown: *RPL8A*, which generates short and long 3′ UTR isoforms, and *RPL22B*, which can exist as pre-mRNA (pre-*RPL22B*) or mature mRNA (*RPL22B*). The scale is indicated in brackets. The bottom cartoon represents transcript structures: UTRs (plain line), coding sequence (Box with arrow), intronic region (line with arrows). **c** Poly(A) tail length profiles of RNA associated with Upf1-TAP (green) compared to total RNA from WT (blue) for *RPL8A* and *RPL22B* isoforms. Poly(A) tails were estimated using Nanopolish. The median poly(A) tail length is indicated. See also Supplementary Fig. 3.

We identified a stable and abundant population of transcripts that lack PTCs and are robustly associated with Upf1. Notably, their levels remained unchanged upon Upf1 deletion (Supplementary Fig. 1a, b). We hypothesized that the population of Upf1-associated transcripts might represent a specific subfraction of mRNAs for each of the hundreds of corresponding genes. To identify this fraction we used direct RNA sequencing (DRS) using Oxford Nanopore Technology, as it enables the sequencing of individual mRNA molecules from their poly(A) tails to their 5′ ends^39^, providing poly(A) tail length information for each transcript without introducing biases associated with PCR amplification (Fig. 3a). We thus applied DRS to the transcripts enriched in the Upf1-purified fraction compared to the WT input and evaluated their poly(A) tail lengths. Unlike Illumina sequencing, DRS also distinguishes specific RNA isoforms that differ by their 5′ end, 3′ end or splicing pattern.

For instance, *RPL8A* mRNA exists in long and short isoforms that Illumina sequencing cannot readily distinguish but were clearly resolved with Nanopore data. DRS revealed a stronger association of the long isoform with Upf1, despite being a minority in the WT Input sample (264 vs. 683 reads, Fig. 3b, left panel). Quantitative analysis showed a 21.3-fold relative enrichment of *RPL8A-long* in Upf1-purified samples (11262 vs. 529 reads), whereas *RPL8A-short* was not enriched (0.82-fold, 622 vs. 760 reads), suggesting a preference of Upf1 for binding the long isoform. Similarly, DRS distinguished *RPL22B* pre-mRNA and mature *RPL22B. Pre-RPL22B* is a known target of NMD^40^. Consistently*, pre-RPL22B* was more enriched with Upf1 than the mature mRNA (5.59-fold vs 3.19-fold). In WT input, the median poly(A) tail lengths were respectively 50 for *pre-RPL22B* and 41 for *RPL22B*, with smaller values in the Upf1-purified fraction (38 and 33 As, respectively). The PTC-containing *pre-RPL22B* retained, however, longer poly(A) tails than mature *RPL22B* (Fig. 3c, right panels).

A similar distribution of poly(A) tail length was observed for full-length RNA molecules, defined by the presence of the signal all along the transcript region. Considering only these molecules for further analysis would strongly reduce the depth and the number of analyzed genes.

To compare Illumina and Nanopore quantifications, we plotted the log2 of fold changes (log2FC) from DESeq2-normalized counts (Supplementary Fig. 3a). Despite biases inherent in both techniques, the global trend showed a moderate positive correlation (Pearson correlation coefficient r = 0.48). To further understand these results, we compared transcript ranks obtained from Nanopore and Illumina sequencing (rank 1 being the most abundant and 12296 the least abundant – Supplementary Fig. 3b). We noticed a strong positive correlation in the three scatterplots (with a Spearman correlation coefficient >0.8), indicating that despite variations inherent to Illumina and Nanopore approaches, the relative estimated order of abundance of transcripts is comparable and consistent between the two methods (Supplementary Fig. 3b). In addition, comparison of Upf1RIP *versus* Input fold-changes are very similar between both sequencing methods (Supplementary Fig. 4a compared to Fig. 1b). In the wake of this observation, enrichments of individual transcripts in Nanopore RIP-seq experiments are consistent with those measured with Illumina (Supplementary Fig. 4b – WT, compared to Fig. 1e).

However, the difference of rank showed a wider range in Upf1-RIP than in *upf1∆* experiments (plot Rank WT – Rank Upf1 RIP compared to Rank WT – Rank *upf1∆* Supplementary Fig. 3c, d), that look similar to foldchange comparisons shown in Supplementary Fig. 3a. Finally, fewer transcripts were classified as enriched (log2FC > 1) in Nanopore sequencing for PTC categories (PTC, XUT/SUT) and noPTC_Upf1-enriched (noPTC_enr). The noPTC category, not enriched in Upf1-RIP in Illumina, showed more transcripts enriched in Nanopore (Supplementary Fig. 3a).

We conclude that Nanopore sequencing provides a robust quantification of Upf1-bound RNAs, comparable to the one obtained by short-read sequencing. The differences inherent to each method (ribodepletion, fragmentation and PCR in Illumina, dT10 priming in Nanopore) and the variable efficiency of purification presumably explain the observed discrepancies. However, since DRS Nanopore technology results in one read per mRNA, and isoform complexity is also defined, this method provided an accurate representation of Upf1-associated mRNA. It also allowed us to investigate enrichment and poly(A) status simultaneously, which is crucial for the next step of our study.

### Most Upf1-associated mRNAs lack a PTC and have a short poly(A) tail

**Fig. 4:**
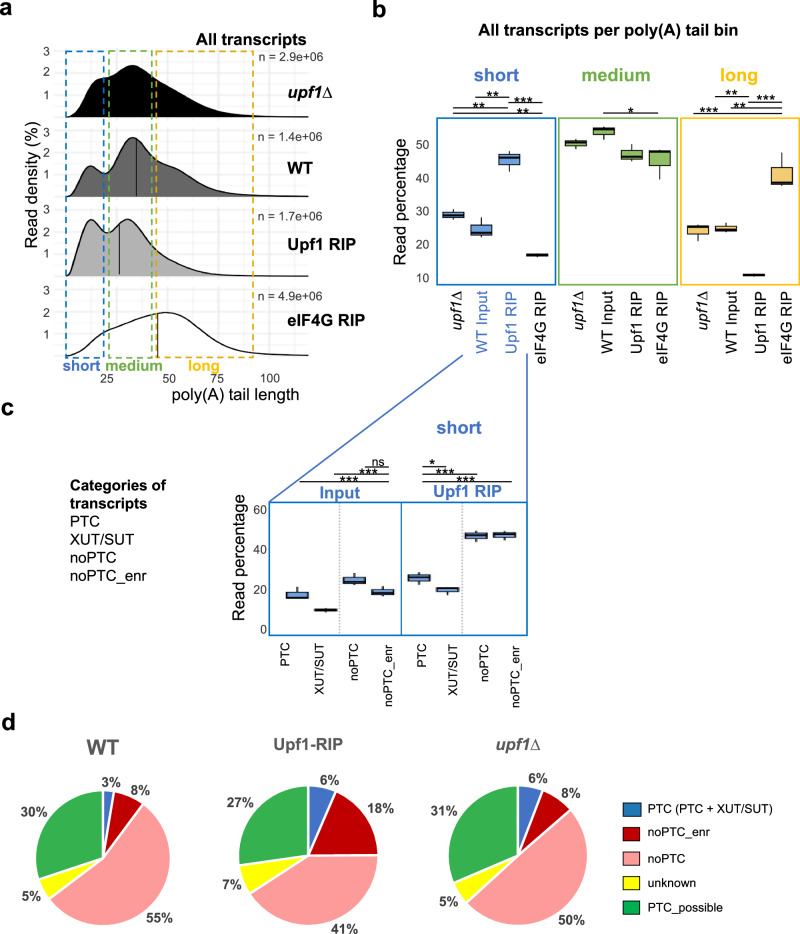
Most Upf1-associated mRNAs lack a PTC and have a short poly(A) tail. **a** Ridge plot showing the global poly(A) tail length distribution across all transcripts from WT (Input) and *upf1∆* total RNA, as well as Upf1-RIP and eIF4G2-RIP samples. The coloured dotted sections indicate: short poly(A) tail (<25 A, blue), medium poly(A) tail (>26 A, <50 A, green), long poly(A) tail (>51 A, yellow). **b** and **c** Boxplots comparing the proportion of poly(A) tail length in short, medium, and long bins across WT (Input) and Upf1-RIP samples transcriptome-wide (B) or comparing subcategories (PTC, SUT/XUT, noPTC and noPTC_enr) between Upf1-RIP and WT input RNAs **c**. Pvalues (Tukey HSD test): ns = *p* > 0.05, * = *p* < 0.05, ** = *p* < 0.01, *** = *p* < 0.001 and **** = *p* < 0.0001. See also Supplementary Table 2 for detailed p-values and Source Data file for numerical values. **d** Pie chart representing the proportion of each transcript category across the indicated samples: PTC containing NMD targets (PTC and XUT_SUT, blue); noPTC-Upf1enr (red), noPTC, (pink); unknown (yellow) and possible NMD (green). All reads are included within each category (comprising short-, medium- and long-tailed reads). See also Supplementary Data 1 and Supplementary Figs. 4–6.

Two families of Upf1-associated transcripts (PTC and noPTC_enr) were identified from short read Illumina sequencing, each characterized by distinct properties such as codon optimality and half-lives (Supplementary Figs. 1c–e, 2a–d). As Upf1 has been found on the same mRNA as the Lsm1-7/Pat1 complex, a complex involved in the degradation of transcripts with short poly(A) tails^41^, it is possible that the newly identified Upf1-targets are bound to Lsm1-7/Pat1 and harbour short poly(A) tails. We assessed the poly(A) tail status from our Direct RNA sequencing (DRS) results combined to RNA immunoprecipitation (Fig. 3a). The poly(A) tail distribution of immunoprecipitated RNAs obtained from Nanopore DRS using either Upf1 (Upf1-RIP) or eIF4G2 (eIF4G-RIP, as a control) were compared to distributions in a wild-type input (WT) or an *upf1∆* RNA pool (Fig. 4a). A tagged version of eIF4G2 - a subunit of the cytoplasmic cap-binding complex eIF4G^42^, which binds capped mRNAs^43^, was used as a qualitative control for purification.

Interestingly, transcripts in the WT input sample exhibited a tri-modal distribution, with peaks at approximately 10 As, 30 As, and 55 As. In contrast, transcripts in the Upf1-RIP sample displayed only two major peaks (10 As and 30 As), while the eIF4G-RIP control primarily contained transcripts with longer poly(A) tails, showing a predominant peak at ∼55 As (Fig. 4a). The poly(A) tail length distribution in the WT input reflects the steady-state status of mRNAs, encompassing both intact mRNA molecules and RNA fragments generated from the 5′ end, as sequencing is anchored at the poly(A) tail. Since sequencing of eIF4G-bound mRNAs captured intact (capped) transcripts from all categories (Fig. 4 and Supplementary Fig. 5), the longer poly(A) tails observed in the purified sample in comparison with total RNA suggest that a fraction of the measured poly(A) tails in the input sample transcripts correspond to decapped degradation intermediates.

To facilitate the visualization and interpretation of the results, reads were classified into three poly(A) tail length bins: short (0–25 A), medium (26–50 A), and long (>51 A) (Fig. 4a, b). Strikingly, the proportion of transcripts with short poly(A) tails was significantly higher in the Upf1-RIP sample (44%) compared to the WT total input (24%) (Fig. 4b). This enrichment of short poly(A)-tailed transcripts was manifest regardless of the level of mRNA enrichment in the Upf1-RIP (noPTC_enr and noPTC categories, Fig. 4c), mirroring the overall profile illustrated in Fig. 4b. Furthermore, this increase in short poly(A)-tailed transcripts in the immunoprecipitated fraction was not due to a technical artefact, as RNAs obtained under similar conditions from eIF4G-RIP experiments displayed extended poly(A) tails. In this sample, medium and long poly(A) transcripts represented 44% and 40% of the total, respectively, confirming a good RNA integrity in these experiments (eIF4G RIP; Fig. 4a, b).

Together, these results indicate that, contrary to expectation and in addition to PTC transcripts, Upf1 is predominantly associated with a short-poly(A)-tailed subpopulation of noPTC transcripts.

The enrichment of short poly(A)-tailed mRNAs within the Upf1-RIP sample was unexpected, given that NMD is considered to be deadenylation-independent^4–6^. Furthermore, we recently demonstrated that NMD targets are inherently unstable and undergo a rapid degradation, which results in longer average poly(A) tails in steady-state^27^. Consistent with this prediction, we observed that PTC-containing NMD targets exhibited longer poly(A) tails compared to other transcript categories, both in the WT input (long tails) and in the Upf1-RIP sample (long and medium tails, Supplementary Fig. 5c). Representative PTC-containing NMD targets such as XUT/SUT or *RPL22B* pre-mRNA corroborated this trend (Supplementary Fig. 5a). However, Upf1-associated PTC-NMD targets exhibited overall slightly shorter poly(A) tails compared to the WT input (Supplementary Fig. 5b). A deeper analysis of short poly(A)-tail proportion variations in the RIP with Upf1 and eIF4G, compared to the short poly(A)-tail proportion in the input showed that while eIF4G captured capped mRNA with extended poly(A) tails compared to the input, no matter the category of transcripts, Upf1 captured on average 2.5-fold more short-poly(A)-tailed mRNA than present in the input. For mRNA with a low level of short poly(A) tails in the input, we observed increases up to 10 times in the Upf1 purified samples, while eIF4G captured overall fewer short-tailed mRNAs (Supplementary Fig. 6).

While the targeting of NMD transcripts occurs independently of deadenylation, this observation suggests that a moderate deadenylation may take place before the resolution of NMD and Upf1 dissociation from RNA. In *upf1∆* cells, NMD targets (XUT/SUT and PTC) displayed shortened poly(A) tails, similar to other categories of mRNA, and consistent with their stabilization in the absence of functional NMD (Supplementary Fig. 5b*upf1∆ vs* WT).

Given that DRS Nanopore technology directly sequences mRNAs, with each read corresponding to a unique initial RNA molecule, multiple assignments were filtered out to keep the most relevant annotation for each read (see Methods for details). We estimated the relative abundance of each transcript within different categories (e.g., PTC, noPTC etc.). The noPTC, noPTC-enr, PTC_pos and unknown categories collectively accounted for 94% of the total reads in the Upf1-RIP sample (Fig. 4d), among which 44% are short-tailed, closely mirroring the overall distribution (Fig. 4b, d). Short-tailed mRNAs thus represent more than 41% of all reads in the Upf1-RIP fraction, while they constitute only 23% of all reads in the WT input. As anticipated, PTC-containing NMD targets constituted a small fraction in the WT input (3%), consistent with their rapid degradation by the NMD machinery. Interestingly, PTC-NMD targets accounted for only 6% in the Upf1-RIP and in the *upf1∆* samples (Fig. 4d). Importantly, we demonstrated that the presence of noPTC mRNAs in the Upf1-RIP fraction was not merely due to background noise, as the selected mRNA population during immunoprecipitation was clearly distinct from that in the input, when considering the poly(A) tail size distribution (Fig. 4a, b and noPTC representative *HHT2* and *TDH3*, Supplementary Fig. 5a). These findings support the conclusion that noPTC short poly(A)-tailed transcripts are a major and specific fraction of the mRNA targeted by Upf1.

### Upf1 targets short poly(A)-tailed mRNAs for degradation

**Fig. 5:**
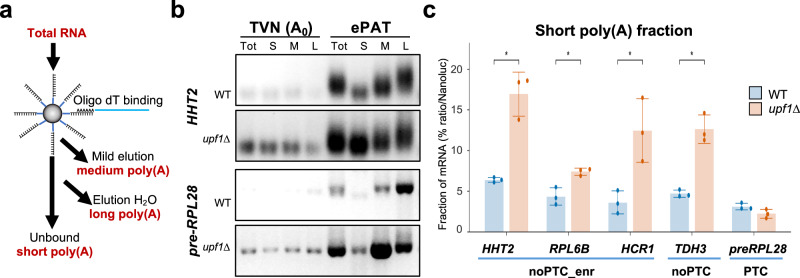
Upf1 targets short poly(A)-tailed mRNAs for degradation. **a** Experimental workflow for poly(A) tail length fractionation using oligodT beads (see also Methods). **b** Ethidium bromide-stained agarose gel showing ePAT and TVN-PAT (A0) results for *HHT2* and pre-*RPL28* across Total RNA (Tot), short (S), medium (M), and long (L) fractions in wild-type and *upf1∆* strains. The results shown are representative of three biological replicates (see Source Data file). **c** Barplot showing the relative proportion of transcripts in the short poly(A) tail fraction for *HHT2, RPL6B and HCR1* (noPTC_enr) *TDH3* (noPTC) transcripts, and *preRPL28* (PTC control), in wild-type (solid-bar) and *upf1∆* strains (outlined-bar). Data represent the mean of three biological replicates; black dots indicate individual replicate values, and error bars represent the standard deviation (SD). Asterix* indicates significant *p*-values (t-test): *HHT2* p = 0.0212; TDH3 *p* = 0.0116). See also Supplementary Fig.7.

Since the population of RNA associated with Upf1 was enriched for transcripts with short poly(A) tails, we decided to further investigate this intriguing observation and test if Upf1 could affect the levels of this specific subfraction. To separate RNAs based on their poly(A) tail length, we adapted a poly(A) fractionation protocol^44^ using oligo dT magnetic beads (NEB). This approach enables the recovery of RNAs lacking or with a short (S), medium (M) and long (L) poly(A) tails (Fig. 5a and see Methods). To assess the poly(A) tail length of individual transcripts, we employed the extended Poly(A) Test (ePAT), a method allowing to visualize the diversity of poly(A) tail lengths^45^. We confirmed the efficient separation of mRNA species with different poly(A) tail lengths across the three fractions using ePAT on *HHT2* (Fig. 5b, WT). ePAT uses a primer anchored to the end of the poly(A) tail to amplify a specific transcript by a PCR over the poly(A) tail^45^. ePAT signal is compared to a TVN control signal, anchored at the beginning of the poly(A) tail with an oligoTVN primer marking the length of the amplicon without the poly(A) tail (A0). We confirmed the efficient separation of mRNA species with different poly(A) tail lengths across the three fractions using ePAT^43–45^ on *HHT2* (Fig. 5b, WT). A significant portion of the signal was detected in the medium fraction, consistent with the average stability of *HHT2* mRNA (half-life of 15.3 min according to Miller et al.^36^). We also tested the *RPL28* pre-mRNA, a well-known PTC-NMD substrate. Most of its signal was found in the L fraction. This result aligns with our DRS results and with the expected pattern of a predominantly long poly(A) tail for an unstable transcript (Fig. 5b). In the *upf1∆* samples, the *HHT2* ePAT signal in the S fraction was increased compared to WT, while the other fractions, including total RNA, remained unaffected. This suggests that the deadenylated forms of *HHT2* are stabilized in the absence of Upf1. In contrast, the *RPL28* pre-mRNA NMD control showed a strong increase of the M fraction in *upf1∆*, reflecting the expected stabilization of this NMD target, which leaves more time for deadenylation (Fig. 5b).

Since ePAT experiments are not quantitative, we next measured transcript levels in the fractions using RT-qPCR. To evaluate fractionation efficiency, we introduced a calibrated pool of in vitro transcribed Nanoluciferase mRNAs with varying poly(A) tail length (A0, A12, A30 and A50) prior to fractionation and estimated their distribution across the three poly(A) fractions by RT-qPCR (Supplementary Fig. 7a). As expected, A0 Nanoluciferase RNA was mostly found in the unbound (S) fraction, while A12 was mainly in the M, and A30 or A50 in the L fractions (Supplementary Fig. 7a).

We compared the relative amounts of representative transcripts in the different fractions: noPTC *HHT2*, *RPL6B*, and *HCR1* (enriched in Upf1-RIP) and *TDH3* (not enriched), PTC *RPL28* pre mRNA, and the *ScR1* ncRNA (used as a deadenylated control), in both WT and *upf1∆* strains. The *ScR1* non-coding control was essentially found in the S fractions, while the PTC-NMD control *RPL28* premRNA was predominantly present in the L fraction in the WT strain, shifting to the M fraction in the *upf1∆* strain, corroborating the ePAT results (Supplementary Fig. 7b). Interestingly, in the WT condition, for all *HHT2, RPL6B*, *HCR1*, and *TDH3* noPTC transcripts, the main signal was detected in the M fraction, suggesting that these transcripts naturally possess medium-length poly(A) tails at steady state. Moreover, a significant increase in the S fraction was measured for these mRNAs in the absence of Upf1 (Fig. 5c and Supplementary Fig. 7b).

Together, these results support a role of Upf1 in the clearance of the short poly(A)-tailed fraction of noPTC transcripts bound by Upf1, regardless of their degree of relative enrichment in Upf1 purification when measured without knowledge of the poly(A) tail size. Variations in the steady-state distribution of different poly(A) tail populations for a given transcript explain why Upf1 binding and relative enrichment can be uncorrelated.

### The NMD machinery stimulates the degradation of short poly(A) tailed mRNAs

**Fig. 6:**
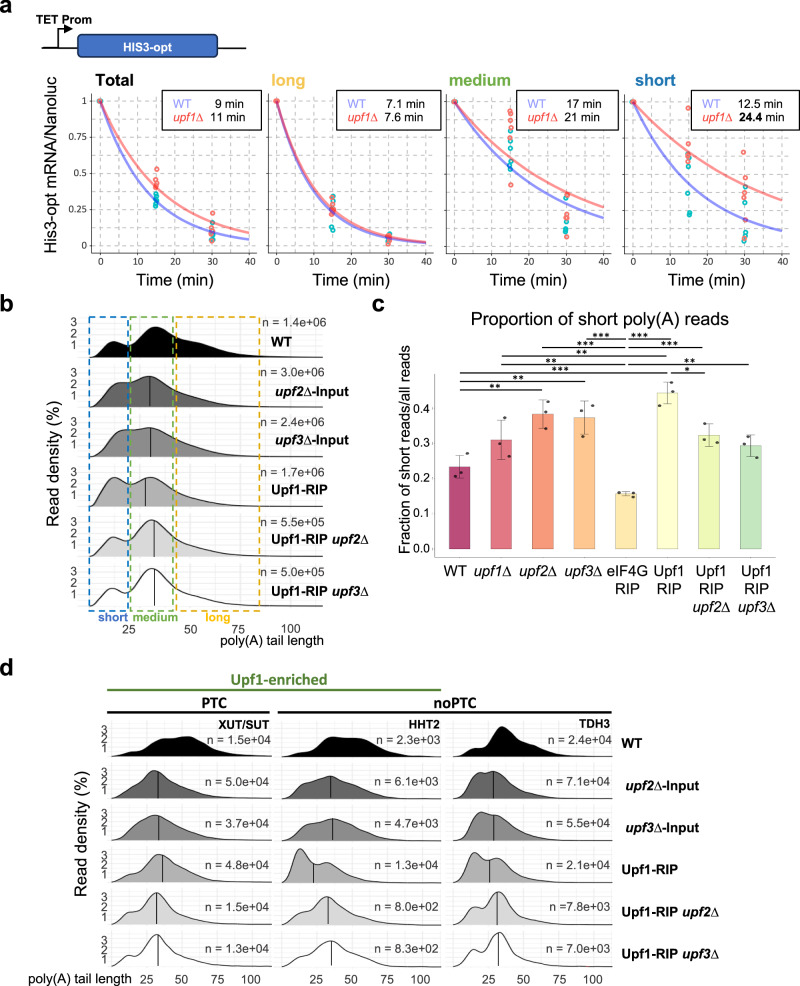
The NMD machinery stimulates the degradation of short poly(A)-tailed mRNAs. **a** Schematic representation of the *HIS3-opt* reporter. Kinetic of degradation of *HIS3-opt* reporter in wild-type (WT, blue) and *upf1∆* (red) strains. Following a poly(A) fractionation as in Fig. 4, the *HIS3-opt* reporter was quantified by RT-qPCR on RNA extracted from cells collected at t = 0, t = 15, and t = 30 min after transcription shutoff. Normalization was performed using Nanoluciferase RNA as an internal control (see Methods). The half-life of HIS3-opt was calculated by fitting an exponential decay model to the data using a nonlinear least squares approach. The degradation constant (**k**) was estimated, and half-lives were computed as t1/2= ln(2)/k, are reported in boxes. Fitted exponential decay curves based on these calculations are displayed. **b** Ridge plot showing the global poly(A) tail length distribution obtained by Nanopore sequencing across WT or *upf2∆* and *upf3∆* context, in input or Upf1 RIP samples. **c** Boxplot comparing the proportions of poly(A) tail length bins (short/medium/long) between Upf1-RIP and Input RNAs in WT, *upf2∆*, and *upf3∆* strains, alongside results from *upf1∆* (as shown in Fig. 4b). Data are presented as mean values +/− SD from three biological replicates. **d** Ridge plot depicting the poly(A) tail length distribution for transcripts in different categories: XUT/SUT (ncRNA), PTC-containing mRNAs, and specific examples such as *HHT2* (noPTC-Upf1enr) and TDH3 (noPTC). Distributions are shown for Upf1-RIP and Input RNAs in WT, *upf2∆*, and *upf3∆* strains. See also Supplementary Fig.8.

To further characterize the role of Upf1 on the stability of short poly(A)-tailed mRNAs, we used a Tet-repressible *HIS3* codon optimized gene (*HIS3-opt*, Fig. 6a) as a representative noPTC reporter in a reconstituted system. *HIS3-opt* gene is fully codon-optimized for translation, and its mRNA is relatively stable^27^. The impact of Upf1 on *HIS3-opt* mRNA stability was evaluated by comparing changes in mRNA levels after transcriptional shutoff with doxycycline in WT and *upf1∆* strains. Additionally, poly(A) RNA fractionation was performed to examine the distribution of *HIS3-opt* mRNA across total RNA samples and in the three sub-fractions (short, medium and long poly(A) tail – Fig. 6a and Methods). No significant differences in mRNA half-lives were observed between the WT and *upf1∆* strains in total RNA samples or in medium and long sub-fractions. However, the half-life of *HIS3-opt* mRNAs in the short poly(A) fraction was increased in the absence of Upf1 (24.4 min vs. 12.5 min in the WT). These findings confirmed the critical role of Upf1 in selectively targeting the short poly(A)-tailed sub-fraction of the *HIS3-opt* reporter for degradation.

Compared to the global kinetics of degradation measured as the half-life in the total RNA sample, the apparent half-lives of *HIS3-opt* in each sub-fraction result from two overlapping processes: deadenylation and degradation. Long-tailed *HIS3-opt* mRNAs have a shorter half-life than medium-tailed mRNAs. This difference is not due to a faster degradation but likely reflects rapid deadenylation, which transitions these mRNAs into the medium-tailed fraction. Similarly, stable medium-tailed mRNAs undergo further deadenylation and eventually shift into the short-tailed fraction. For the last short-tailed fraction, deadenylation no longer contributes to mRNA turnover, as these transcripts cannot be further deadenylated. Therefore, the half-life measured in this fraction primarily reflects the effects of mRNA decay. These findings suggest that the stability of short poly(A)-tailed mRNAs critically depends on the presence of Upf1 in this reporter system (Fig. 6a and see also Discussion).

Given that NMD can potentially target mRNAs as a result of individual ribosome frameshifting^19^, we investigated the affinity of Upf1 for modified versions of the *HIS3-opt* reporter to address this possibility. These constructs included versions where possible stop codons in frame +1 and +2 were either removed (no stop) or retained a possible premature termination codon in frame +1 and in frame +2 (Stop +1 + 2 – see Supplementary Fig. 8a). To assess Upf1’s association with *HIS3-opt* mRNA we performed RT-qPCR on Upf1-RIP samples and WT input, normalizing the results to *TDH3*, a non-enriched but abundant endogenous mRNA (Supplementary Fig. 8b). The *HIS3-opt* mRNA was enriched 2.28-fold in Upf1-RIP compared to input, which is in the same range as the enrichment observed for *HHT2* (5.7-fold; Fig. 1e). This enrichment indicates that Upf1 is associated with the *HIS3-opt* transcript in a comparable manner to that of other noPTC_enr mRNAs. We evaluated the affinity of the no-stop reporter (with no possibility to find a stop in a frameshifted phase), and the Stop+1 + 2 reporter (possibly frameshifting and running into a Stop codon in other phases). The analysis of *HIS3-opt* reporter mRNA levels in the RIP fraction relative to total RNA input revealed similar enrichment across the *HIS3-opt*, no-Stop, and Stop+1 + 2 constructs (2.28, 2.66, and 2.58, respectively; Supplementary Fig. 8b). Therefore, the contribution of a frameshift effect for the tested transcript is negligible.

Having established that Upf1 is involved in the clearance of the short poly(A)-tailed population of noPTC mRNAs, we further questioned whether this effect depends on other NMD factors, since Upf1-mediated decay pathways independent of other NMD factor have been described^46^. To assess the impact of NMD factors on Upf1’s role in the clearance of short poly(A)-tailed noPTC mRNAs, we conducted Nanopore DRS on Upf1-RIP fractions in *upf2∆* or *upf3∆* strains and compared the results to their corresponding total RNA inputs (Fig. 6b–d). As observed in the *upf1∆* strain (Fig. 4), the poly(A) profiles of inputs were largely comparable across WT, *upf2∆*, and *upf3∆* strains, but with a notable increase in the proportion of short poly(A)-tailed mRNAs in *upf2∆* and *upf3∆* inputs. Strikingly, in the Upf1-RIP samples, the proportion of short poly(A)-tailed mRNAs was reduced in the *upf2∆* and *upf3∆* compared to the WT context (Fig. 6c). In addition, the enrichment of PTC and noPTC_enr targets observed with Upf1-purified fraction in a WT context was reduced to 1 (no change) when Ufp2 or Upf3 was absent (Supplementary Fig. 4a, b). These results strongly suggest that Upf1’s ability to bind short poly(A)-tailed noPTC transcripts could be altered in the absence of Upf2 or Upf3. As expected, the proportion of short-, medium- and long-tail species by categories in *upf2∆* and *upf3∆* strains were very similar to the ones observed in *upf1∆*, but the proportions of mRNA immunoprecipitated with Upf1 in these conditions were importantly decreased (Supplementary Figs. 4, 5c, 8c).

Taken together, these findings highlight the critical role of Upf1 and the NMD machinery in the degradation of short poly(A)-tailed noPTC mRNAs. The use of a reporter system further validated Upf1’s functional role in clearing the short poly(A)-tailed fraction of stable noPTC mRNAs.

### Upf1 and cofactors Upf2 and Upf3 trigger the decapping of noPTC mRNAs

**Fig. 7:**
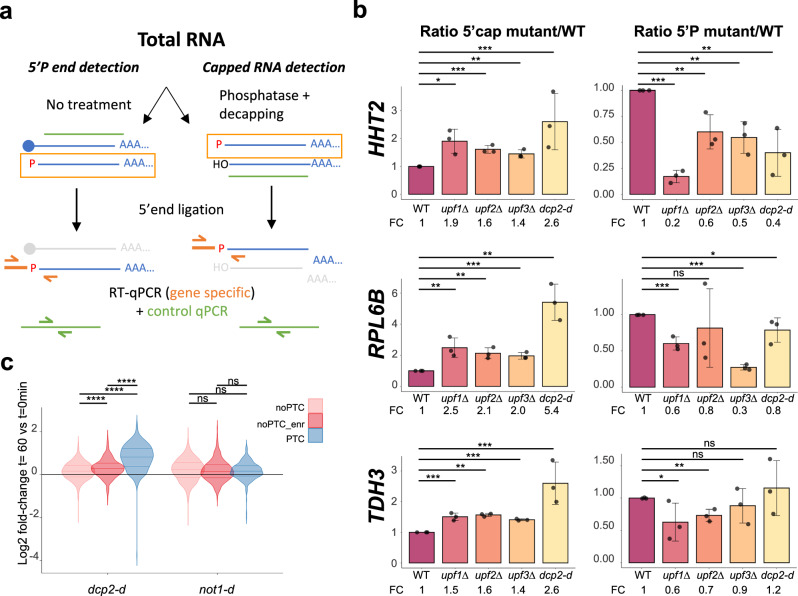
Upf1 and cofactors Upf2 and Upf3 trigger the decapping of noPTC mRNAs. **a** Schematic representation of the test to quantify 5′P and 5′cap transcript populations. **b** Ratio of 5′cap (left) or 5’P(right) ends of specific transcripts (*HHT2*, *RPL6B*, and *TDH3*) in *upf1∆ upf2∆, upf3∆* and *dcp2-degron* mutants strains compared to WT (mutant/WT). All the values are normalized on endogenous ScR1 as a control. Average fold change (FC) compared to WT is indicated below each barplot. Pvalues (T.test): ns = *p* > 0.05, * = *p* < 0.05, ** = *p* < 0.01, *** = p < 0.001. Data are presented as mean values +/− SD from three biological replicates. **c** Violin plot of the transcript level Log2 fold-change of RNA levels at t = 60 min versus *t* = 0 min, per categories of transcript (data from Chappleboim et al., 2022). Pvalues (T.test): ns = *p* > 0.05, * = *p* < 0.05, ** = *p* < 0.01, *** = *p* < 0.001, **** = p < 0.0001.

In order to evaluate if Upf1 and its cofactors Upf2 and Upf3 affect the decapping of noPTC targets, similarly to their role in NMD, we developed a qPCR-based test to quantify either the relative 5′P or 5′capped fractions of a transcript in the same sample (Fig. 7a). Ligation of an oligonucleotide to the accessible and phosphorylated 5′ end of RNA molecules was performed either without any particular treatment or following a dephosphorylation and decapping step. In one case, we quantified all the decapped species encompassing the region amplified by the qPCR reaction, while in the other, only capped mRNA was quantified. The 5′cap levels ratio compared to wild type for the noPTC_enr (*HHT2* and *RPL6B*) and noPTC (*TDH3*) representative mRNAs increased significantly in *upf1∆, upf2∆ and upf3∆* strains (1.4 to 2.5-fold), though not to the same extent as in a *dcp2*-degron strain following depletion of Dcp2 (2.6 to 5.4-fold). Conversely, the relative amounts of available 5′P ends decreased, particularly for the noPTC_enr mRNAs (Fig. 7b). While our data suggest that the decapping defects demonstrated here are the direct consequence of Upf1 binding to short poly(A) mRNA, we cannot fully exclude the hypothesis that stabilized NMD substrates accumulating in the absence of Upf1 could, somehow, decrease decapping efficiency for other mRNAs.

To evaluate the sensitivity of the noPTC (enriched or not in Upf1-RIP) and PTC transcript categories to decapping or deadenylation, we assessed changes in transcript levels 60 min after Dcp2 or Not1 inactivation using a dataset from Chappleboim et al.^47^ We observed that transcripts from the PTC and noPTC_enriched categories increased the most upon decapping inactivation (dcp2), while remaining unaffected by the deadenylase complex inactivation (not1 - Fig. 7c).

Overall, our findings support the involvement of Upf1, Upf2 and Upf3 in promoting the degradation of noPTC mRNAs, and especially on noPTC_enr mRNAs, via stimulation of their decapping. These observations, combined with the observed loss of Upf1’s ability to recognize both short poly(A) and canonical PTC-NMD targets in *upf2*∆ or *upf3*∆ strains, strongly suggest that short poly(A)-tailed RNA degradation is mediated through a mechanism involving the full NMD machinery.

## Discussion

Four decades of studies on Upf1 have highlighted its crucial role in shaping the transcriptome, primarily by targeting mRNAs with premature termination codons through NMD^5,48,49^. While the precise mechanism by which Upf1 detects these mRNAs remains an open question, PTC-containing reporters showed that Upf1 triggers mRNA degradation independent of deadenylation^4–6^. In this study, we comprehensively characterized Upf1 targets and found evidence that the NMD machinery also specifically targets short poly(A)-tailed mRNAs to facilitate their degradation via accelerated decapping, revealing a repertoire of NMD-regulated transcripts much larger than previously recognized.

**Fig. 8:**
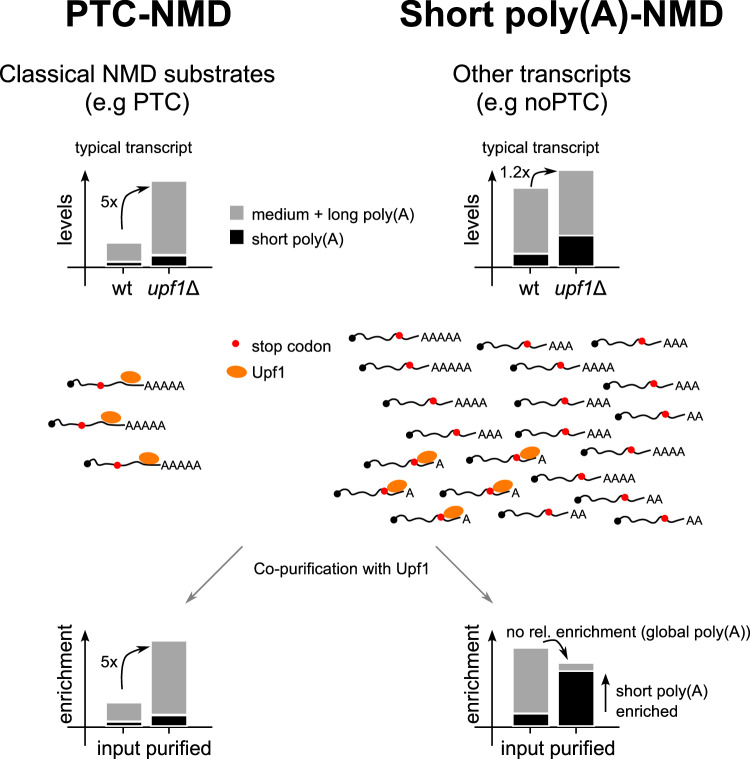
Schematic representation of expected results for a typical **PTC-containing NMD substrate** and for a **short poly(A)-NMD substrate** when tested in the absence of Upf1 or when evaluated in the fraction associated with Upf1. Obvious changes in mRNA levels escaped previous investigations because the poly(A) status is crucial for the evaluation of real changes in the total RNA and in the Ufp1-associated fraction. Created in Inkscape^66^.

First, we confirmed transcriptome-wide that Upf1 triggers the degradation of PTC-containing mRNA independently of deadenylation, as indicated by the presence of mRNA with relatively long poly(A) tails in the Upf1-associated fraction (Fig. 4). PTC-containing mRNAs are uniformly recognized by the NMD machinery. In contrast, short poly(A)-tailed mRNAs form a newly identified subset specifically bound by Upf1, which were previously undetected due to their low abundance within the heterogeneous “non-NMD-sensitive” mRNA population. Although both are targeted by the NMD machinery, PTC and short poly(A)-tailed noPTC transcripts behave differently (Fig. 8). PTC transcripts are homogeneous, unstable, and likely associated with one or two Pab1 molecules (mainly medium- and long-tailed in WT–Fig. 4), and were previously found enriched in the monosome fraction^32^. In contrast, noPTC mRNAs are heterogeneous, stable, and efficiently translated, with only the short poly(A)-tailed subpopulation targeted by the NMD machinery, as revealed by Nanopore sequencing, which highlighted Upf1’s specific affinity for this subset.

Our results imply that enrichment values for a majority of transcripts in the Upf1 purified fraction based on Illumina sequencing are not truly representative, as they compare short poly(A)-tailed mRNAs with all poly(A) length isoforms for mRNAs originating from a given gene (see also Fig. 8). Despite the relative abundance of short poly(A)-tailed mRNAs in association with Upf1, largely surpassing the amounts of PTC-containing mRNA (Fig. 4), these RNAs have not been considered until now as targets of the NMD machinery. The proportion of short poly(A)-tailed mRNA was likely underestimated in our study, as DRS does not detect fully deadenylated transcripts. Additionally, the variable rates of 3′ to 5′ degradation across different mRNAs may contribute to the observed heterogeneity of the degradation profiles for any given RNA, although this aspect could not be assessed with DRS either.

Fractionation experiments allowed us to reveal a specific effect of Upf1 on short poly(A)-tailed mRNA levels (Fig. 5). Using a reconstituted noPTC *HIS3-opt* reporter, we identified variations in the intrinsic stabilities of short-, medium- and long-poly(A) tailed mRNA subpopulations, which would otherwise be indistinguishable in total RNA samples (Fig. 6). Furthermore, we demonstrated that Upf1 and the NMD pathway significantly affect the levels (endogenous *HHT2*, *RPL6B*, *HCR1* and *TDH3)* and stability (*HIS3-opt*) of noPTC short poly(A)-tailed mRNAs (Figs. 5 and 6).

These findings questioned how Upf1 is recruited to this specific short poly(A)-tailed subfraction. Considering that a single Pab1 molecule binds approximately 25–30 adenines^50^, we estimated the number of Pab1 molecules associated with each bin of poly(A) tail length from Nanopore data: short tails (<25 adenines) likely lack Pab1, medium tails (25–54 adenines) are bound by one Pab1 molecule, and long tails (>55 adenines) are associated with two or more Pab1 molecules. This also suggests that shortened poly(A) tails could be accompanied by a loss of Pab1 binding. Consistent with our findings and previously published results^9,49^, and by analogy with the “Faux 3′ UTR” model (i.e., PTC-NMD), a lack of Pab1 in proximity of the stop codon would lead to a translation termination defect that stimulates the recruitment of NMD factors, in particular Upf1. Similarly to the PTC-NMD, where the long distance between Pab1 and the stop codon dictates mRNA recognition by the NMD machinery, we speculate that the absence of Pab1 could also trigger NMD in a way that we propose to name “short poly(A)-NMD”. In both scenarios, translation termination could be impaired due to the absence of Pab1’s stimulating effect on the translation termination factor eRF3^51^ and Upf1, Upf2 and Upf3 could be required to solve the same translation termination defect, both by stimulating ribosome recycling and by recruiting the decapping complex for RNA degradation.

We have previously shown that the population of *HHT2* mRNAs in the WT total RNA sample harbour short poly(A) tails and is most likely poorly associated with Pab1, as the Pab1-associated *HHT2* poly(A) tails are much longer (see Figure EV2 from Audebert et al.^27^). Strikingly, Upf1-associated *HHT2* harbours shorter tails than the total RNA. These results suggest that Pab1 could be absent on the *HHT2* mRNAs associated with Upf1 and Lsm1. This is consistent with previous work demonstrating that the NMD machinery can be active even in cells lacking Pab1 and for reporters devoid of a poly(A) tail^52^. Although we could not measure the effect of tethering Pab1 nor eRF3 on noPTC_enr mRNAs (not shown), we could evaluate the stimulating effect of NMD factors on the decapping of noPTC and noPTC_enr mRNAs and found a greater sensitivity of the most affected categories (PTC and noPTC_enr transcripts) to decapping (Fig. 7).

Altogether, our findings converge towards a unified model in which short poly(A) tailed mRNAs are degraded via a specialized pathway that requires the NMD machinery. We recapitulated our findings in a data-based summary illustration of typical behaviour observed for PTC-containing NMD substrates and for short poly(A)-NMD substrates (Fig. 8).

How could the short poly(A)-NMD pathway be integrated into the current models of mRNA degradation that explain the degradation of deadenylated RNA? Oligoadenylated mRNAs are recognized by the Lsm1-7/Pat1 complex, which stimulates decapping and subsequent mRNA degradation^53–55^. We previously identified the presence of both Upf1 and Lsm1-7/Pat1 complex in the same mRNPs^1^. This initially seemed paradoxical as NMD is known to be deadenylation-independent and consequently occurs on mRNAs with long poly(A) tails^4–6^ (also shown in this study Supplementary Fig. 5b, c). We conciliated this observation in light of our current findings: as Upf1 targets short poly(A)-tailed noPTC mRNAs intended for general decay, it is compatible with a detection by the Lsm1-7/Pat1 complex.

The short poly(A)-NMD could be an important cellular process to post-transcriptionally regulate histone transcript levels in yeast. Cell-cycle-dependent histone mRNA decay is not well understood in yeast and whether Upf1 plays a role is unknown. Mammalian histone transcripts are deadenylated^56^, and display a specific post-transcriptional regulation by Upf1 independently of Upf2 and Upf3, and mediated by an interaction with the cofactor SLPB that recruits Upf1 on a stem loop on these mRNAs^57,58^. Contrary to human, yeast histone transcripts are polyadenylated and SLBP is not present, whether Upf1 plays a role in their regulation is unknown. Histone post-transcriptional regulation seems also driven by *LSM1* in yeast as histone transcripts strongly accumulate in a *lsm1∆* mutant following hydroxyurea treatment^59^.

In our study, we found that Upf1 has an intrinsic affinity for the noPTC_enr transcripts (Figs. 1, 2) that are more susceptible to being affected by the short poly(A)-NMD route. These mRNAs include half of the histone transcripts (*HTA2*, *HTB2*, *HHF2*, *HHT2*, and *HTZ1*). The others (*HTA1*, *HTB1*, *HHF1*, and *HHT1*) belong to noPTC and *HHO1* to PTC_pos categories either because Upf1 has a lower affinity for them or the distribution of oligoadenylated versus longer poly(A) forms is different. Additionally, we identified a role of Upf1/2/3 in the decapping of *HHT2* (Fig. 7). Altogether, our observations suggest that both Lsm1 and Upf1 could coexist on the same mRNP to trigger the decapping and targeted degradation of histone transcripts in yeast by short poly(A)-NMD. It is possible that the evolution has conducted Upf1 to post-transcriptionally regulate Histone transcripts in yeast by a different route than those in mammals.

Finally, it would be valuable to determine the proportion of mRNAs that are decapped and “normally” degraded before the last Pab1 is removed, in comparison to those addressed to short poly(A)-NMD. This comparison would provide a more comprehensive estimate of the overall contribution of short poly(A)-NMD to general mRNA decay and its impact on the transcriptome. Future research will be essential to deepen our understanding of the significance of poly(A) tail length as a regulatory feature in mRNA degradation. This could further reveal how the NMD machinery shapes the transcriptome in eukaryotic systems beyond its original characterization in the degradation of PTC-containing transcripts.

## Methods

### Yeast and Bacterial strain

All *Saccharomyces cerevisiæ* strains were derived from the BY4741 strain (leu2Δ0, his3Δ1, ura3Δ0, met15Δ0, MATa), obtained from the Euroscarf deletion collection (http://www.euroscarf.de) or from the TAP-fusion library^60^. New yeast strains were generated by lithium acetate-based transformation of the BY4741 strain with a PCR product containing a selection marker cassette flanked by 50 bp arms located upstream and downstream of the targeted ORF.

Upf1(Ymr080w)-TAP strain LMA2194 (MATa, ura3∆0, his3∆1, leu2∆0, met15∆0, YMR080W-TAP:HIS3MX6) and eIF4G2(Ygl049c)-TAP strain LMA3314 (MATa, ura3∆0, his3∆1, leu2∆0, met15∆0, YGL049C-TAP:HIS3MX6) were obtained from the *S. cerevisiæ* TAP fusion library^60^.

The Ymr080w-HTP strain LMA3730 used for the Illumina RIPseq experiment was obtained by transformation of the Ymr080w-TAP LMA2194 strain with the HpaI linearized plasmid pGIM1287 TAP2CRAC.

*Escherichia coli* strain NEB 10-beta (NEB Cat#C3019) was used for plasmid construction and amplification. All the plasmid inserts were verified by sequencing.

C-terminal TAP-tagged strains originated from the Ghaemmaghami et al.^60^ collection. Deletions were tested by PCR amplification of the modified locus.

### Growth conditions and media

#### Yeast

Yeast strains were grown at 30 °C in YPD (20 g/L glucose, 10 g/L yeast extract, 20 g/L bacto-peptone, and 20 g/L bacto-agar for plates only) or in synthetic media without uracil to select transformants and maintain plasmids with the URA3 marker. Cultures were grown in the exponential phase at OD_600_ of 0.6 – 0.8.

#### Bacteria

*Escherichia coli* strains were grown in LB medium at 30 °C to prevent TetO7 promoter pop-out. The medium was supplemented with Ampicillin (50 µg/ml) for plasmid selection.

### Plasmids construction

pCM189-HIS3opt plasmid (pCM189-NFLAG-HIS3-100 - TETO7-NFLAG-HIS3-100 #GIM1640), originally described by ref. ^27^ was modified by replacing the original HIS3-opt coding sequence by a Bam HI (NEB Cat# R0136S]) and PstI (NEB Cat# R0140S) digested fragment with all internal out-of-frame stop codons removed (No-Stop) or with a unique stop codon in frame +1 and +2 (Stop +1 + 2). The modified HIS3 sequences were cloned into pCM189 by ligation with T4 DNA ligase (NEB Cat#M0202T), amplified in Escherichia coli and sequenced (Eurofins genomic) generating plasmid pCM189-HIS3nostop (#GIM1803) and pCM189-HIS3stop12(#GIM1804).

### RNA immunoprecipitation

TAP-tagged Upf1-containing yeasts were grown at 30 °C in 2 L YPD in the exponential phase (OD_600_ of 0.6–0.8). Cells were centrifuged, rinsed with cold water and immediately frozen at −80 °C until lysis. Yeast cells were lysed under FastPrep mixing in lysis buffer (20 mM Hepes-K pH 7.4, 100 mM KOAc, 10 mM MgCl2, RNasin 1/1000 (Promega Cat#N2511), Protease inhibitor (Roche Cat#11836170001) using acid-washed glass beads (program yeast, *Saccharomyces cerevisiæ*, 6.0 m/sec/Quickprep/40 sec 2 times). Lysates were centrifuged 10 min at 1500 x *g* at 4 °C, and the supernatant was collected. Upf1-TAP-associated RNA was immunoprecipitated using Dynabeads M-270 epoxy (ThermoFisher Cat#14302D) coated with Rabbit IgG (Sigma-Aldrich Cat# I5006) in lysis buffer (as described in Namane et al.^61^) supplemented with 20% Triton X-100. The lysate was incubated for 1.5 h at 4 °C with gentle agitation. After incubation, the supernatant was discarded, and stringent washing steps were performed to ensure specific binding using lysis buffer without protease inhibitors. Elution was carried out in TES buffer (2% SDS, 1X TE, 30 mM EDTA) at 65 °C for 15 min. RNAs were extracted from the supernatant containing the Upf1-immunoprecipitated RNAs using phenol/chloroform and precipitated with ammonium acetate.

### Criteria of category status assignment

mRNAs were first sorted based on their propensity to generate NMD-sensitive RNA isoforms with alternative 5′ends according to Malabat et al.^18^ mRNAs homogeneous in 5′ (**noPTC**) were defined arbitrarily as generating less than 10% of NMD-sensitive 5′ end isoforms, whereas those producing more than 10% of NMD-sensitive 5′ end isoforms were considered **possible-PTC**. In order to refine this assignment, mRNAs were reassigned **PTC** if they were either stabilized more than two-fold upon *upf1∆ (*visualized as a MAplot Supplementary Fig. 1A) or assigned direct NMD target (classes A and B in Heyer and Moore, 2016). The **unknown** category also corresponded to mRNA not present in other categories or for which we did not have enough information from our or previous datasets.

### Reverse transcription and quantitative PCR

RNAs were extracted from yeast after three phenol extractions (Total RNAs) or one (RIP). Contaminating genomic DNA was removed from 50 µg of total RNA by DNase treatment using 10 units of Turbo DNase (Invitrogen Cat# AM2239) at 37 °C for 20 min and DNAse was eliminated by incubating 5 min with the Inactivation Reagent following the manufacturer’s instructions. Priming was performed using random hexamers, denatured at 65 °C for 5 min before being placed in ice. Reverse transcription was carried out using SuperScript II Reverse Transcriptase (Invitrogen Cat# 18064022) in a mix containing 5X First-Strand Buffer, 0.1 M DTT, and 10 mM dNTPs. The reaction was incubated at 42 °C for 45 min and 70 °C 10 min for heat inactivation. Targeted transcripts were amplified using specific primers (at 5 µM final concentration, see Primer Table for description) in a reaction mixture containing SYBR Green Mix (Bio-Rad Cat# 1725272) and incubated 95 °C 10 min; 40 cycles (95 °C 10 sec; 60 °C for 30 sec).

### RNA fractionation by poly(A) tail length

To fractionate RNA by poly(A) length, we adapted the Meijer et al.^44^ protocol, using oligo d(T)_25_ magnetic beads (NEB) and sequential elution steps. 50 to 100 µg of total RNA were incubated in 100 µL of 2X Binding Buffer (40 mM Tris-HCl pH 7.5, 1 M LiCl, 2 mM EDTA) at 65 °C for 2 min, then 100 µL of prepared oligo d(T)_25_ magnetic beads were added to the mixture and incubated for 15 min at RT, placed on a magnet, and the supernatant, containing unbound RNA (short or A- tails), was collected and precipitated using ammonium acetate and ethanol. The beads were washed twice with Washing Buffer (20 mM Tris-HCl pH 7.5, 0.5 M LiCl, 1 mM EDTA); once with Low Salt Washing Buffer (20 mM Tris-HCl pH 7.5, 0.2 M LiCl, 1 mM EDTA). Two successive elutions were performed with 1) 20 µL of 0.075X SSC buffer for 10 min at RT (medium poly(A) tails) and 2) 20 µL of nuclease-free water for 10 min at RT (long poly(A) tails).

### Extension poly(A) Test (ePAT)

Extension Poly(A) Test (ePAT) was performed by adapting the Jänicke et al.^45^ protocol. 1 µg of total RNA was denaturated with CS1400 5 min at 80 °C and brought to 37 °C 1H with 1 µL of Klenow exo- polymerase (NEB Cat# M0212S) for 3′ extension in 1X First strand buffer, 5 mM DTT, 500 µM dNTP, and RNAsin(Promega Cat#N2511). Reaction mix was denatured 5 min at 80 °C to inactivate Klenow, and brought to 55 °C prior to adding 1 µL of SuperScript III Reverse Transcriptase (Invitrogen Cat# 18080093), incubated 1H at 55 °C. The resulting cDNA was amplified using the universal reverse primer (CS1402) and a gene-specific forward primer with Phusion polymerase (ThermoFisher Cat#F530L) according to the manufacturer’s instructions. In parallel, a TVN-PAT reaction was performed using CS1401, a TVN-anchored primer to visualize the amplicon length without poly(A) (A0). Products were loaded on a 2.5% agarose gel in TBE 1X.

### Degradation kinetics and RNA half-life measurement

Wild-type yeasts and *upf1∆* strains were transformed with the pCM189-HIS3opt plasmid, and grown as described in Audebert et al.^27^. Degradation kinetics were performed by the addition of doxycycline at 10 µg/ml (Sigma-Aldrich Cat#D3447) and cell collection at multiple time points (t_0_, t_15_, and t_30_ minutes). Total RNA was extracted by hot phenol/chloroform extraction and precipitated with ammonium acetate and ethanol.

RNAs were fractionated based on their poly(A) tail length as described in the ***RNA Fractionation by Poly(A) tail length*** section, and RNA half-life measurement was performed by RT-qPCR using MFR918 and LA076 for HIS3-opt, GB1659 and GB1733 for Nanoluciferase standard. Half-life from RT-qPCR results was calculated using a nonlinear regression and the exponential decay function e^-(t-l)*k^, as described in Audebert et al.^27^

### Illumina sequencing

TruSeq mRNA stranded Library Preparation Kit (Cat#20020594) from Illumina was used according to the manufacturer’s instructions. Single read 75 sequencing was performed on a NextSeq500 flowcell (Illumina Cat#20024906) at the Pasteur Biomics facility.

### Nanopore direct RNA sequencing

For library preparation (SQK-RNA002 kit from Oxford Nanopore Technologies), we used 100–500 ng of total RNA or 50 ng of immunoprecipitated RNA (RIP), previously ribodepleted with Oligo d(T)_25_ magnetic beads selection (NEB Cat#S1419S), following the manufacturer’s instructions. Sequencing was performed on a MinION or GridION device, using FLO-MIN106D flow cells (R9.4.1) for 72 h.

### 5′P/5′cap quantification

10 µg of WT or *upf1∆* DNAse-treated total RNA was directly ligated to 100 pmol of GB1476 primer with 10 U of T4 RNA ligase HC (NEB) overnight at 16 degrees (5′P samples) or first dephosphorylated with 10U of Antarctic phosphatase (NEB) and decapped with 5U of decapping enzyme (NEB) (5′cap samples). RNAs were purified using AMPure RNA XP beads after each of these steps. Reverse transcriptions were performed on the resulting 5′P and 5′ cap samples using N6 random primer and Induro reverse transcriptase (NEB) according to the manufacturer’s instructions. 10-, 20-, 40- and 80-fold dilutions of RT samples were used to quantify *HHT2*, *RPL6B* and *TDH3* transcripts. These transcripts were amplified using respectively reverse primers GB1694, GB1777 and GB799, and common primer GB1773 (at 5 µM final concentration each), and *ScR1* was used as a control for normalization and amplified using GB987 and GB798 at the same concentration (see also Table primer in Supplementary information) in a reaction mixture containing SYBR Green Mix (Bio-Rad Cat# 1725272) and incubated 95 °C 10 min; 40 cycles (95 °C 10 sec; 60 °C for 30 sec).

### Quantification and statistical analyses

#### Statistics and Reproducibility

All the experiments presented in this study were made in triplicate from three biological replicates. Box plots elements, boundaries and whiskers correspond to standard R conventions. Whiskers extend to the most extreme data point that is still at a distance of less than 1.5 times the interquartile range. Individual points outside that value are represented as outliers. See also Source Data File for more information.

#### Illumina and CRAC sequencing analysis

Raw sequencing data were demultiplexed using bcl2fastq and assessed for quality with FastQC. Adapter sequences were removed using cutadapt. Reads were aligned to the SC288 reference genome (Saccer3) with the STAR aligner. Exon annotation was carried out using the GTF file for *Saccharomyces cerevisiæ* (R64-1-1.104) from ENSEMBL. Read quantification was performed with featureCounts, a function from the Subread package. Subsequent data processing and analysis were conducted using R, and statistical analysis were conducted using the SARTools package with the “shorth” option for Deseq2 normalization^62^.

Supplementary Data 2, related to Fig. 1, shows the normalized count of Illumina sequencing data.

#### Nanopore direct RNA sequencing analysis

Raw data were basecalled with Guppy basecaller. Reads were mapped using minimap2 and the option “-k14 -ax splice -uf -G 2000 -secondary=no” to the SC288 reference genome Saccer3.fa. Alignements were sorted and indexed using samtools 1.19. Poly(A) length was determined using Nanopolish v0.13.2^39^. Subsequently, data were filtered to retain only reads that passed the quality filter “PASS” and reads assigned in doubloons were filtered using a customed R script (10.5281/zenodo.14615891). Subsequent data processing and analysis were conducted using R, and statistical analysis was conducted using the SARTools package with the “shorth” option for Deseq2 normalization^62^.

Supplementary Data 3, related to Fig. 3, shows the normalized count of Nanopore sequencing data.

### Reporting summary

Further information on research design is available in the Nature Portfolio Reporting Summary linked to this article.

## Supplementary information

Supplementary Information

Description of Additional Supplementary Files

Supplementary Data 1

Supplementary Data 2

Supplementary Data 3

Reporting Summary

Transparent Peer Review file

## Source data

Source Data

## Data Availability

The data supporting the findings of this study are available from the corresponding authors upon request. Sequencing data reported in this paper have been deposited at GEO: GSE283053 (Illumina RIP-seq), GSE299782 (CRAC) and GSE284490 (Nanopore). The sequencing data’s count generated in this study is provided in the Supplementary Data 2 (RIP seq Illumina) and in Supplementary Data 3 (RIP seq Nanopore). Source data for the figures and Supplementary Figs. are provided as a Source Data file. Source data are provided with this paper.

## Code availability

All original code has been deposited at Zenodo at 10.5281/zenodo.14615891.
