# Supplementary material for: Nonsense-mediated mRNA decay factors target short poly(A)-tailed mRNAs lacking a premature termination codon
Source: Nat Commun. 2026 Apr 17;17:5333. doi: 10.1038/s41467-026-72132-1 (PMC13273070; doi:10.1038/s41467-026-72132-1)
Supplement: Supplementary file 1 — Supplementary Information [file 41467_2026_72132_MOESM1_ESM.pdf]

Nonsense-Mediated Decay mainly targets short poly(A)-tailed mRNAs lacking a premature termination codon

Supplementary Information List

Supplementary Fig. 1 –
Characteristics of transcripts per category, related to Figure 1

Supplementary Fig. 2 –
Relative codon usage in categories PTC, noPTC and noPTC-enriched, related to Figure 2

Supplementary Fig. 3 –
Characteristics of transcripts per category, related to Figure 3

Supplementary Fig. 4–
Fold-changes of the RIPseq experiments driven using Nanopore DRS, related to Figure 4 and Figure 6

Supplementary Fig. 5 –
Poly(A) tail characteristics per category of transcript, related to Figure 4

Supplementary Fig. 6–
Changes in the fraction of short poly(A) transcripts in RNA-Immunoprecipitations, related to Figure 4.

Supplementary Fig. 7 –
Quantification of poly(A) subfractions during oligodT fractionation, related to Figure 5

Supplementary Fig.8–
Effect of Upf2 or Upf3 depletion on Upf1-associated transcripts, related to Figure 6

Supplementary Table 1
Primer Table

Supplementary Table 2
Resource Table

Supplementary references

Supplementary Fig. 1

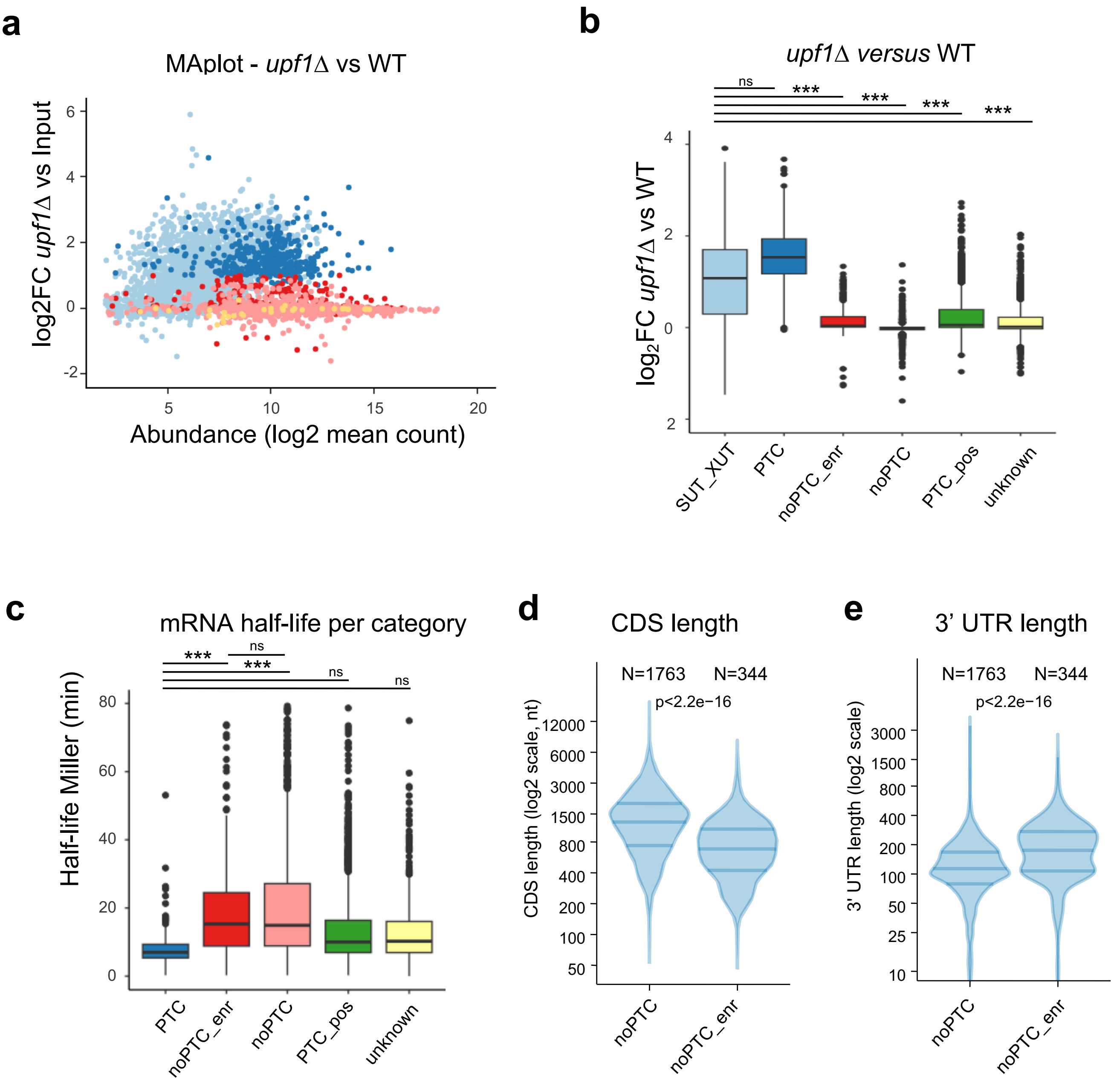

Supplementary Fig. 1. Characteristics of transcripts per category, related to Figure 1

a MAplot representing the log2FC between *upf1*Δ and WT as a function of abundance (A = log2 mean count in *upf1*Δ or in Upf1-RIP and WT respectively). Each transcript type is represented as follow: XUT/SUT (light blue), PTC (dark blue), noPTC-Upf1enr (red), noPTC (pink), and mitochondrial transcripts as a control (yellow). Dotted lines indicate a 2-fold threshold (log2FC = 1).

b Boxplots comparing *upf1*Δ vs WT log2FC for mentioned categories of transcripts. Pvalues (Tukey HSD test) : ns = p> 0.05, * = p<0,05, ** = p<0,01, *** = p<0,001 and **** = p<0,0001. Detailed pvalues of all Figures are listed Table S2.

c Boxplot representing the transcript half-lives (in minute, according to Miller *et al.*, 2011) per category.

d Violin plot reprensenting the coding sequence length accross the noPTC and noPTC_enr categories.

e Violin plot reprensenting the 3'UTR length accross the noPTC and noPTC_enr categories.

Supplementary Fig. 2

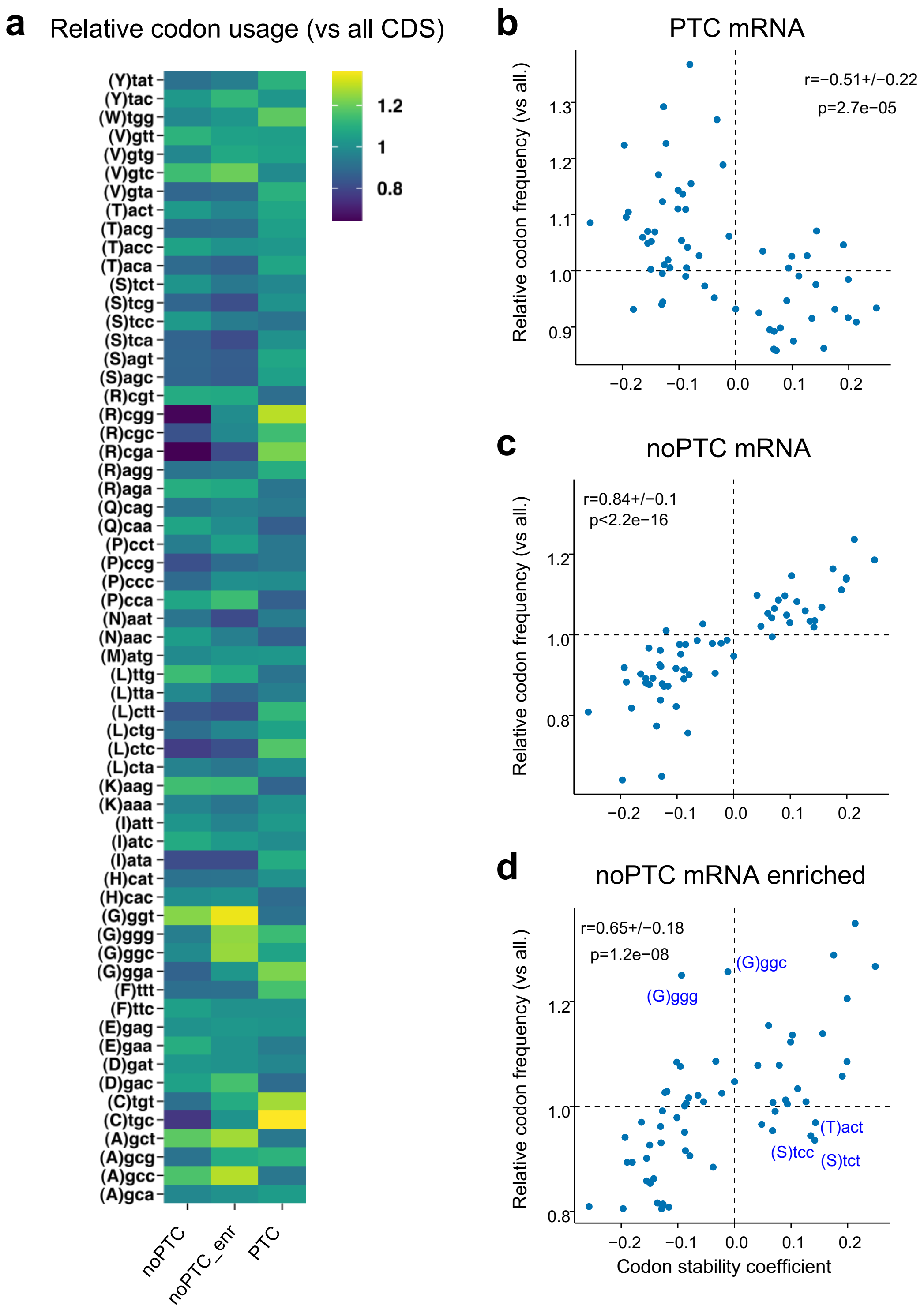

Supplementary Fig. 2. Relative codon usage in categories PTC, noPTC and noPTC-enriched, related to Figure 1 and 2

a Heatmap representing the relative codon usage of coding sequences in categories noPTC, noPTC_enr and PTC in comparison to all *S. cerevisiae* coding sequences.

b-d Scatterplots representing the relative codon frequency (ordinate) function of the codon stability coefficient (abscissa) across PTC (**b**) noPTC (**c**) and noPTC_enr (**d**) mRNAs. Pearson correlation coefficients were calculated with 95% confidence interval and the corresponding p values are indicated for each scatterplot.

a

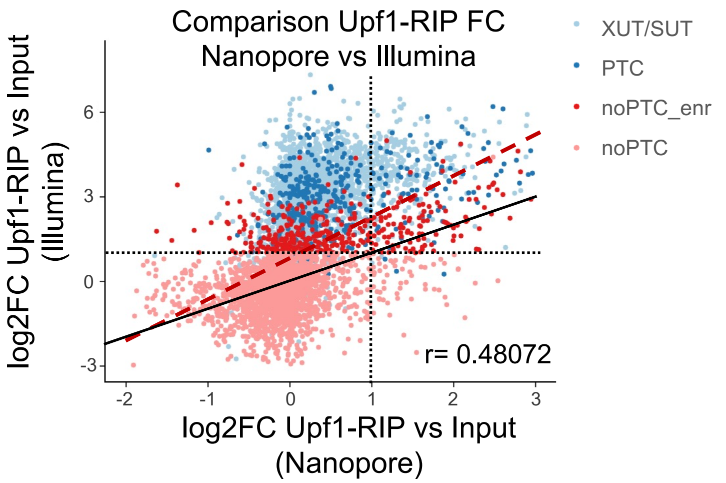

Category	log2FC>1 Illumina	log2FC>1 Nanopore
PTC	432	155
XUT/SUT	2219	697
noPTC_enr	345	161
noPTC	0	107

b

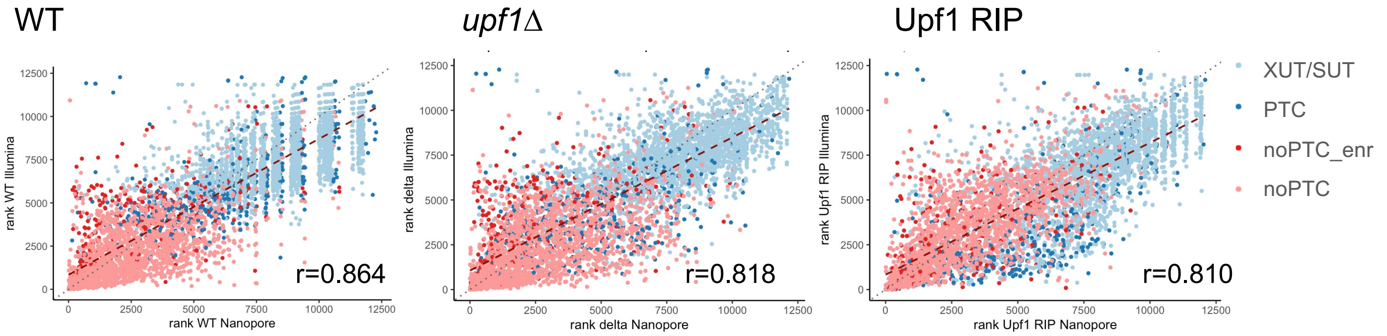

c

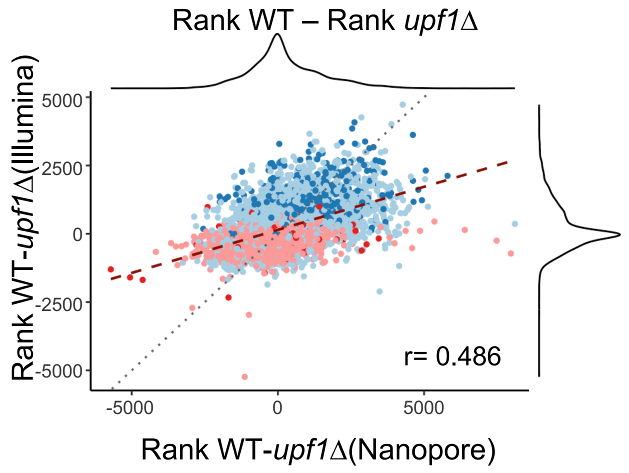

d

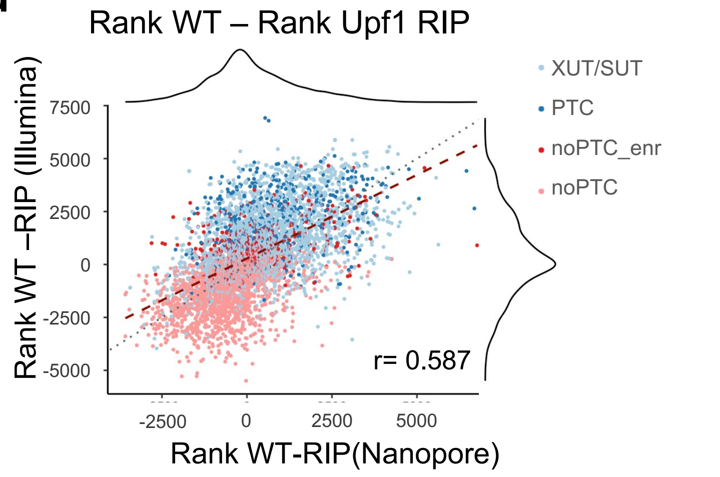

Supplementary Fig. 3 Characteristics of transcripts per category, related to Figure 3

a Scatterplot comparing log2FC between Upf1-RIP and WT in Nanopore (abscissa) and Illumina (ordinate), for transcripts from PTC: XUT/SUT and PTC, and noPTC categories: noPTC Upf1-enriched (noPTC-enr) and not enriched (noPTC). Dotted lines (black) indicate a two-fold threshold (log2FC = 1). The diagonal is indicated in black (plain line). The dashed red line represents the linear regression trend line (Pearson's correlation coefficient, r = 0.48072 p value < 2.2 10−16). The number of transcripts with a log2FC between Upf1-RIP and WT >1 are listed in the table (left panel).

b Rank of the read counts (from rank 1 - the most abundant mRNA to rank 12290 - the less abundant) in Illumina *vs* Nanopore datasets, in the WT input (left), upf1Δ (center) and Upf1 RIP experiment (right panel). The diagonal is indicated in black (dotted line). The dashed red line represents the linear regression trend line (Pearson's correlation coefficient, respectively r = 0.864 (WT), r = 0.818 (*upf1*Δ), r = 0.810 (Upf1 RIP), p values < 2.2 10^{-16}). Represented transcripts categories are : PTC(XUT/SUT and PTc, and noPTC (Upf1-enriched (noPTC-enr) and not enriched (noPTc).

c Comparison of transcript rank in WT – rank in *upf1*Δ, in Nanopore (abscissa) and Illumina (ordinate) experiments for transcripts from PTC: XUT/SUT and PTC, and noPTC categories. The dashed red line represents the linear regression trend line (Pearson's correlation coefficient, r = 0.486 p-value < 2.2 10^{-16}). See also Supplementary Data 3. The diagonal is indicated in black (dashed line).

d Simarily to (**c**) comparison of transcript rank in WT – rank in Upf1-RIP, in Nanopore (abscissa) and Illumina (ordinate) experiments. Pearson's correlation coefficient, r = 0.587 p value < 2.2 10−16. See also Supplementary Data 3. Resented transcripts categories are : PTC(XUT/SUT and PTC), and noPTC (Upf1-enriched (noPTC_enr) and not enriched (noPTC). The diagonal (black dotted line) and the linear regression trend line (dash red line - Pearson's correlation coefficient, r = 0.587 p-value < 2.2 10^{-16}) are indicated.

Supplementary Fig. 4

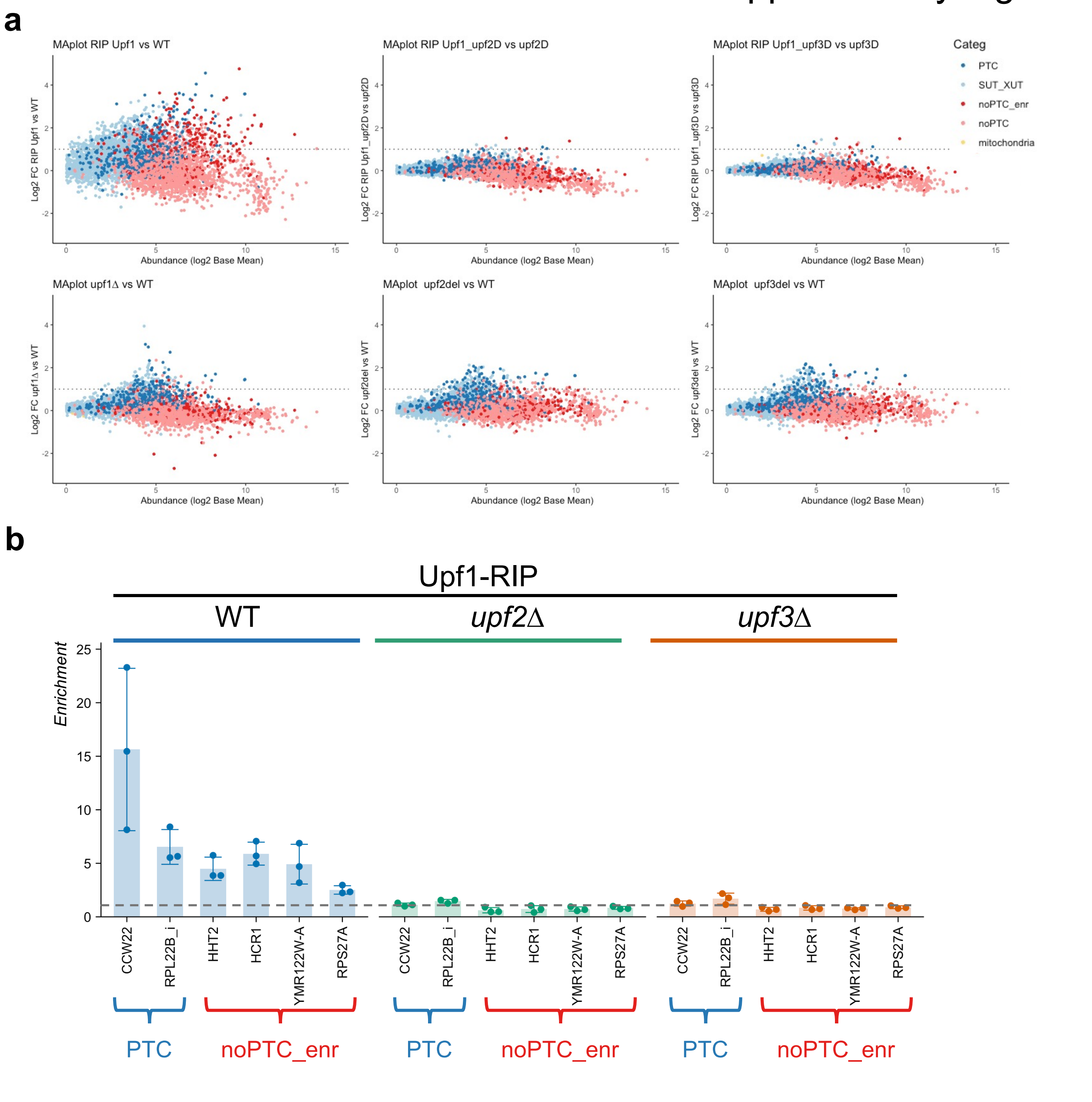

Supplementary Fig. 4. **Fold-changes of the RIPseq experiments driven using Nanopore DRS, related to Figure 4 and Figure 6**

a MAplots representing the log2FC as a function of abundance (A = log2 mean count in each pair of Nanopore sequencing experiment) between two-by-two datasets : Upf1 RIP and WT (upper left) or Upf1 RIP in *upf2*Δ and *upf2*Δ (upper center) *or upf3*Δ (upper right), *upf1*Δ and WT (lower left), *upf2*Δ and WT (lower center) and *upf3*Δ and WT (lower right). Each transcript type is represented as follow: XUT/SUT (light blue), PTC (dark blue), noPTC-Upf1enr (red), noPTC (pink), and mitochondrial transcripts as a control (yellow). Dotted lines indicate a 2-fold threshold (log2FC = 1). See also SupplementaryData3_GSE284490_Nanopore.xlsx.

b Enrichment (number of normalized reads from RIP / input) for examples from PTC (*CCW22 and RPL22B_i*) and noPTC_enr (*HHT2, HCR1, YMR122W-A, RPS27A*) categories of transcripts, in the WT (left), or in the *upf2*Δ (center) or *upf3*Δ (right) contexts. The dashed line represent the ratio RIP/input = 1, corresponding to no change between the reads in RIP and in input, what is expected in case of background. See also SupplementaryData3_GSE284490_Nanopore.xlsx.

Supplementary Fig. 5

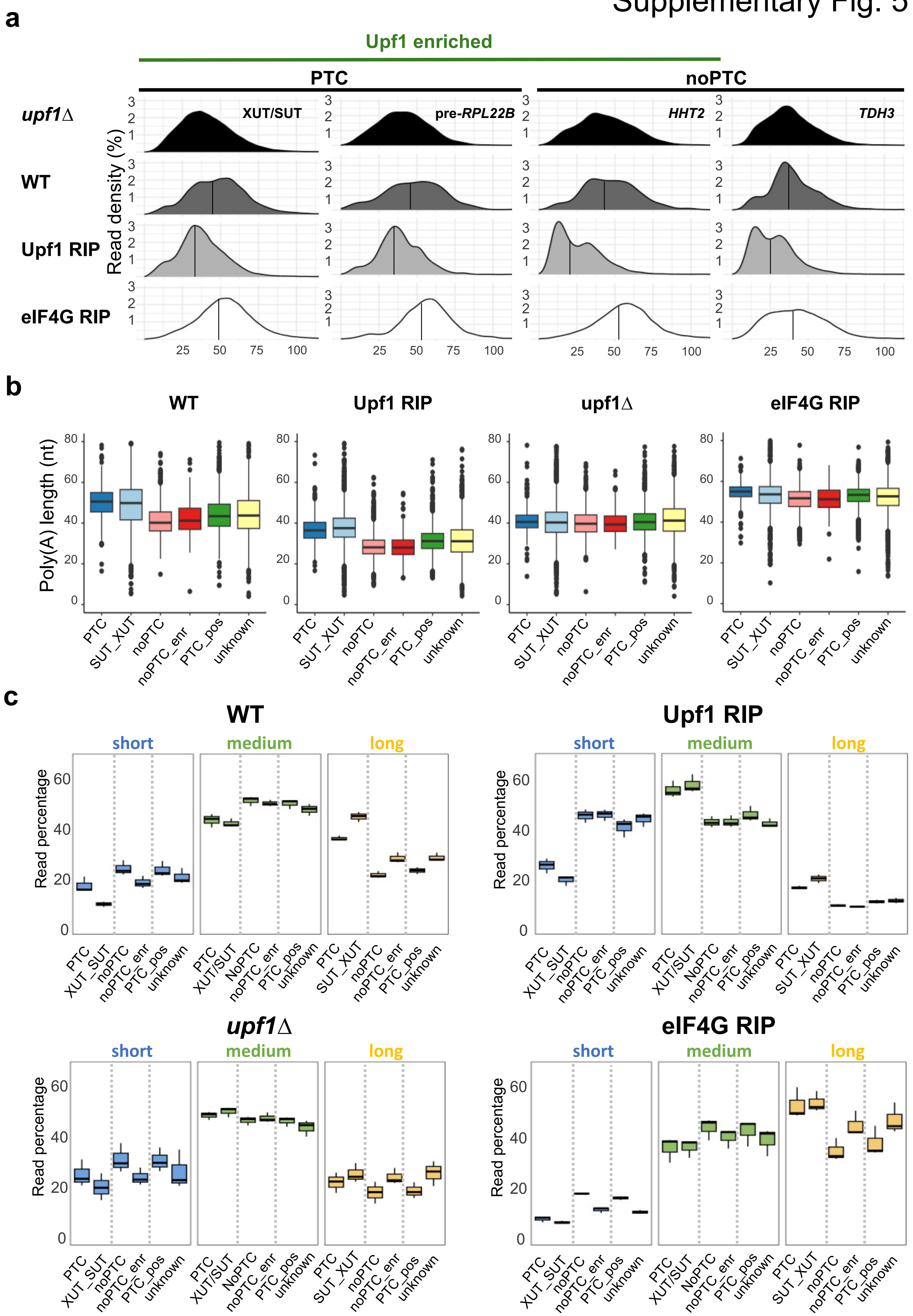

Supplementary Fig. 5. Characteristics of transcripts per category, related to Figure 4

a Ridge plot representing the poly(A) tail length distribution across representative examples in WT (Input) and *upf1*Δ total RNA, as well as Upf1-RIP and eIF4G2-RIP samples. XUT/SUT and *pre-RPL22B* are representative examples of **PTC**-containing NMD targets, HHT2 is a representative example of **noPTC-enr**, and TDH3 is a representative example of the **noPTC** category. Upf1-enriched target are mentioned (green line) while PTC/noPTC categories are indicated in black.
b Boxplots showing the proportion of poly(A) tail length in short (<25A), medium (>26, <50A) and long (>51A) bins across WT, Upf1 RIP, *upf1*Δ and eIF4G-RIP samples. The analysis shows the different sub-categories: PTC (dark blue), SUT/XUT (blue), noPTC (pink), noPTC-enr (red), PTC_poss (green), and unknown (yellow).
c Boxplots displaying the proportion of poly(A) tail length in bins (short, medium and long) across WT (Input) and *upf1*Δ total RNA, and Upf1-RIP and eIF4G2-RIP samples. Pvalues (Tukey HSD test): ns = p> 0.05, * = p<0,05, ** = p<0,01, *** = p<0,001 and **** = p<0,0001. See also Supplementary Table 2 for detailed pvalues.

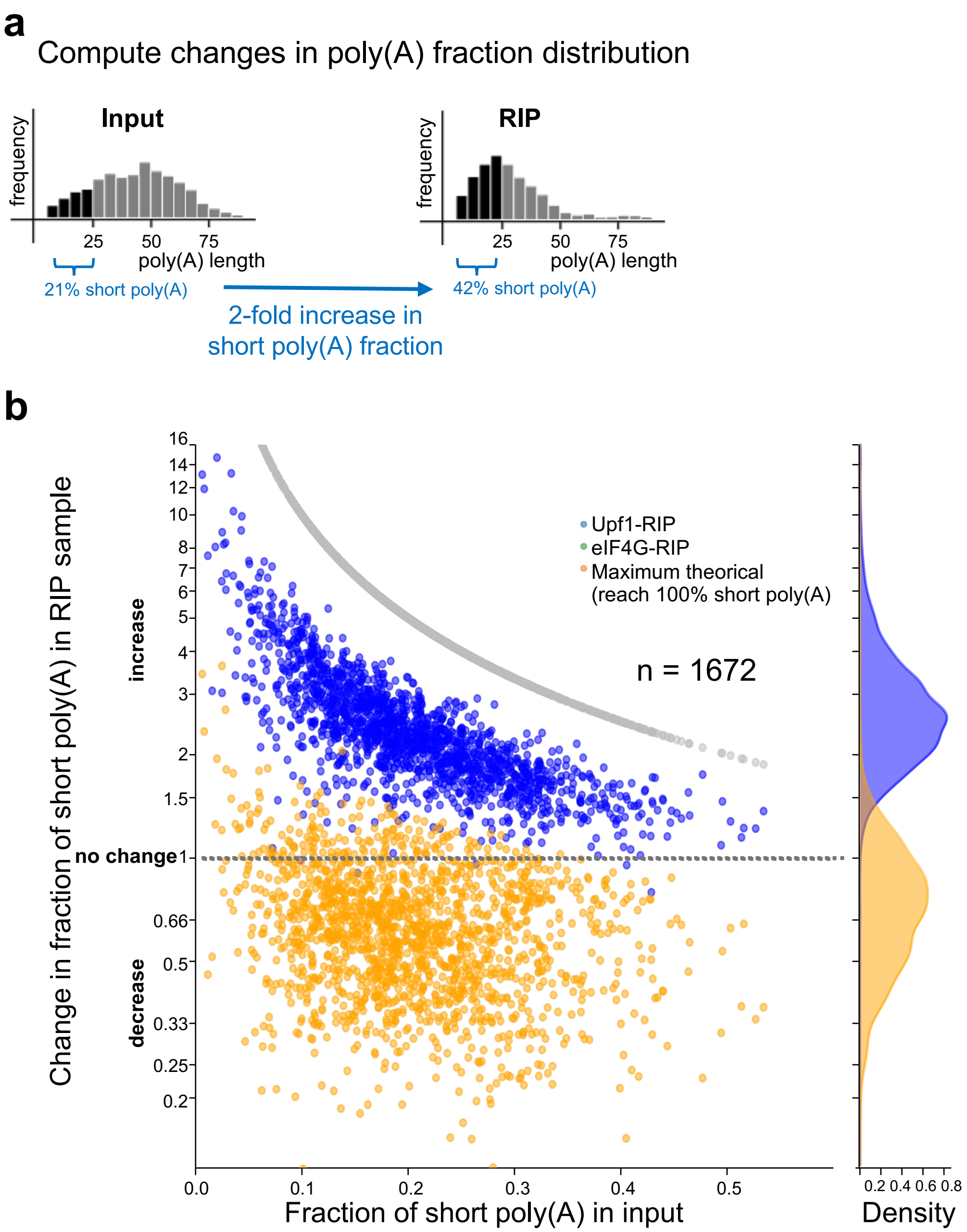

Supplementary Fig. 6. Changes in the fraction of short poly(A) transcripts in RNA-Immunoprecipitations.

a Compute changes in poly(A) fraction distribution. Theorical Example of a transcript that contains 21% of short poly(A) in the input, and 42% in the RNA-immunoprecipitated fractions. **b** Scatterplot representing the change in short poly(A) fraction in RIP compared to the fraction of short poly(A) in the input. The density is shown on the right panel. 1672 transcripts with a minimum of 100 reads are kept.

Supplementary Fig. 7

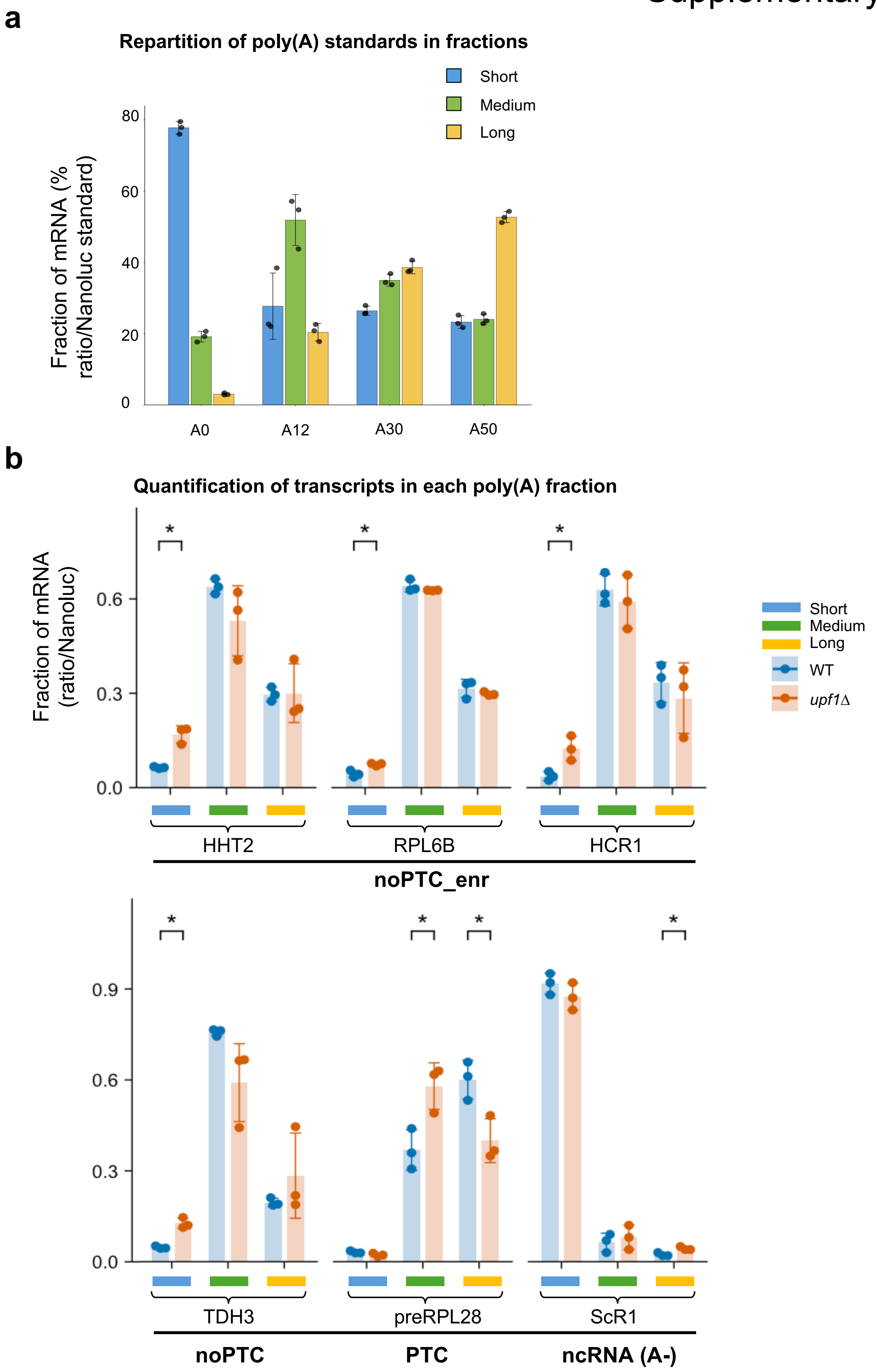

Supplementary Fig. 7. Relative quantification of poly(A) subfractions during oligodT fractionation, related to Figure 5

a Barplot reporting the relative proportion of each poly(A) tail length in short (S), medium (M), and long (L) fractions of Nanoluciferase mRNA standards with calibrated poly(A) tail lengths (A0, A12, A30 and A50 - see also STAR methods). Data were obtained by RT-qPCR, and show the mean of three biological replicates, black dots represent the value for each replicate, error bar represent the standard deviation (Sd.

b Barplot reporting the relative proportion of poly(A) tail length in each fractions **short** (blue), **medium** (green) and **long** (yellow) of **noPTC_enr** transcripts (*HHT2, RPL6B* and *HCR1),* **noPTC** *(TDH3),* **PTC** NMD contr*ol (preRPL28),* and a **deadenylated control** ncRNA *(ScR1),* in wild-type (blue) and *upf1*Δ strains (pink). Data show the mean of three biological replicates, black dots represent the value for each replicate, error bar represent the SD. * (significant P-values T.test.) : (*preRPL28* – Medium) p = 0.02401; (*preRPL28* – Long) p = 0.02279; (*HHT2* – Short) p = 0.02124; (*TDH3* – Short) p = 0.01165; (*RPL6B* – Short) p = 0.01197 ; (*HCR1* – Short) p = 0.02319

Supplementary Fig. 8

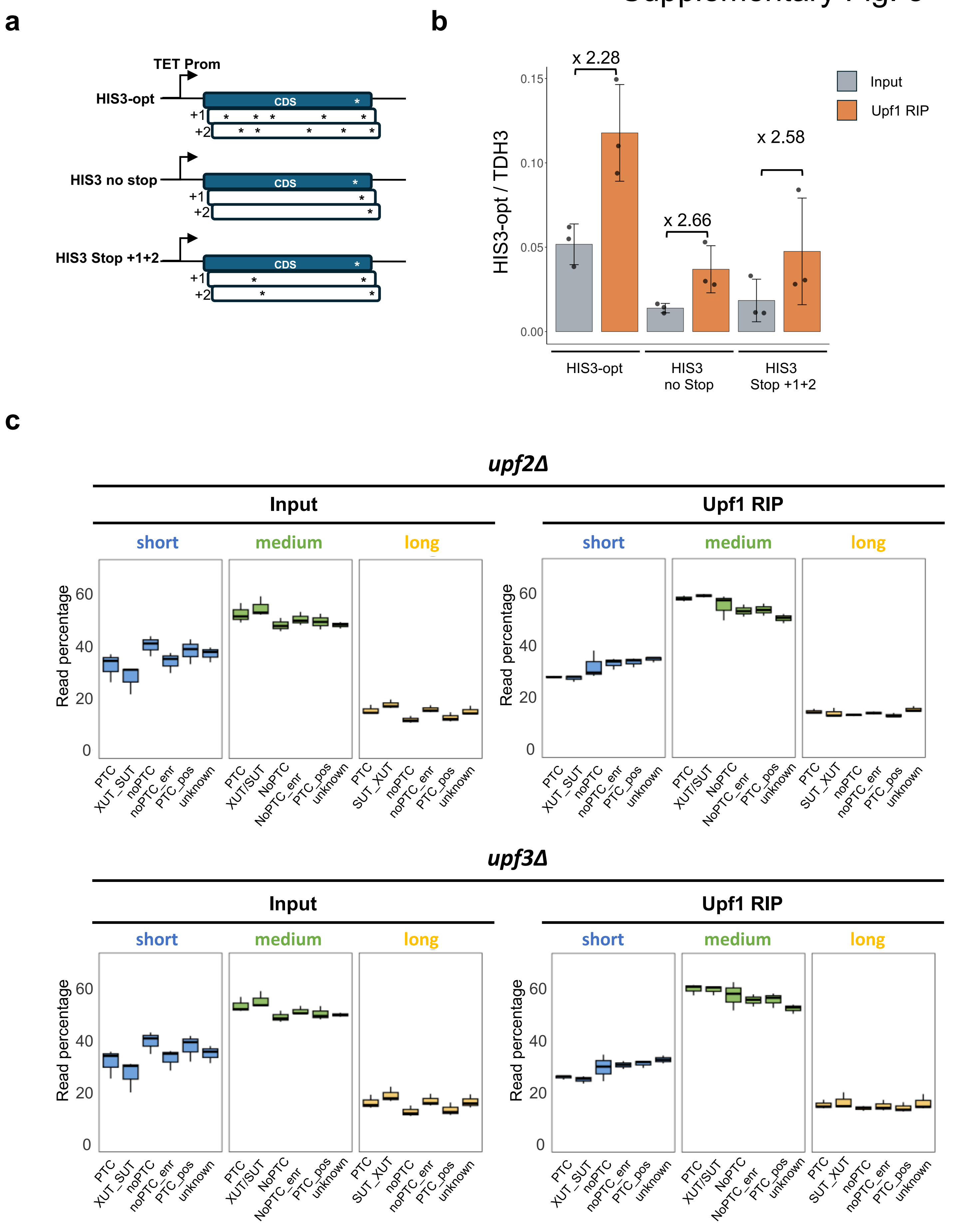

Supplementary Fig. 8 Effect of Upf2 or Upf3 depletion on Upf1-associated transcripts, related to Figure 6

a Schematic representation of the reporters: *HIS3-opt* ("normal"), *HIS3* no stop (no stop in frame +1 or +2), and *HIS3* Stop +1+2 (with a stop in frame +1 and +2). Stop codons are indicated by stars.

b Relative quantification of *HIS3-opt* in Upf1-RIP versus WT input, normalized by *TDH3* (non-enriched control). Data show the mean of three biological replicates, black dots represent the value of each replicate, error bar represent the SD.

c Boxplot displaying the proportion of poly(A) tail length in bins short, medium and long across Upf1-RIP and Input RNAs for *upf2*Δ or *upf3*Δ strains, comparing indicated transcript categories as in Figure 2b.

Supplementary Table 1
Primer Table

Oligonucleotide	Sequence	Description
LA83	CCGCTCTAACCGAAAAGGAAG	Cyc1 cloning and DNA probe - for HIS3opt qPCR
LA84	GGGACCTAGACTTCAGGTTG	Cyc1 probe - for HIS3opt qPCR
CS887	CCATCTCACTGTTGAGACGG	RPL28intron forward for qPCR
CS888	CTCAGTTTGCGATGGAAGAG	RPL28intron reverse for qPCR
CS1400	GCGAGCTCCGCGGCCGCGTTTTTTTTTTTT	ePAT primer for all
CS1401	GCGAGCTCCGCGGCCGCGTTTTTTTTTTTTV N	TVN-PAT for all (size control, 12A)
CS1402	GCGAGCTCCGCGGCCGCG	ePAT primer for all - step 2
CS1455	CGTTTACAGACATAATTGCGGG	HHT2 for ePAT
GB798	GGTAACATCATCCCATCCTCC	TDH3 forward for qPCR
GB799	CAAGACCTTACCGACAGCC	TDH3 reverse for qPCR and 5'P/5'cap qPCR
GB987	CAATTATCCGACTGATATGTGC	SCR1 forward for qPCR
GB998	GGTAGTTCTGGGTCCTTAG	SCR1 reverse for qPCR
GB1402	GCCAGGTACTGTTGCCTTGAG	HHT2 forward for qPCR
GB1403	GTCGGTCTTGAAATCTTGAGCG	HHT2 reverse for qPCR
GB1476	rCrUrUrUrCrCrCrUrArCrArCrGrArCrGrCrUrCrU rUrCrCrGrArUrCrUrNrNrNrNrNrNrN	RNA primer for 5' Ligation
GB1659	ATGGTTTTTACTTTAGAAGATTTTG	Nanoluc_all forward for qPCR
GB1694	TTTCTGGCAGCCTTGGAG	HHT2 reverse for 5'P/5'cap qPCR
GB1728	GTTGAAAACCTGTTTTACTAACG	RPL28 Intron for ePAT
GB1729	AAACTTGGTCTGACCGTGAT	Nanoluc forward index 1 for qPCR - A12
GB1730	AAACTTGGTCTGACACATCG	Nanoluc forward index 2 for qPCR - A30
GB1731	AAACTTGGTCTGACGCCTAA	Nanoluc forward index 3 for qPCR - A50
GB1732	AAACTTGGTCTGACTGGTCA	Nanoluc forward index 4 for qPCR - A0
GB1733	GAAGAAACACCACCTTGTTC	Nanoluc reverse qPCR - All and each index
GB1734	CGTCTCAGCCGGTAAAGGTC	RPL28 exon for ePAT
GB1761	CCCATTGGACGAAGCTTTGTC	HIS3noSTOP and HIS3+1+2 for qPCR
GB1762	CGACAACAGCGTATGGTCTG	HIS3noSTOP and HIS3+1+2 for qPCR
GB1773	CACGACGCTCTTCCGATC	Truseq Forward for 5'P/5'cap qPCR
GB1777	GGGCAGTCATTTCGTTGAAT	RPL6B reverse for 5'P/5'cap qPCR
GB1574	CACAATCTCCTACGTACAC	qPCR HCR1_rev
GB1407	GTCCTGAAATTGTGAATGTCTATATTCCC	qPCR HCR1_fwd
GB1779	GAGAATGCGTTGATCTGAAAATAC	qPCR RPL6B_fwd
GB1780	GATTACATATCCACTTACCTTTCCG	qPCR RPL6B_Rev

Supplementary Table 2
Statistical Comparisons and p-values

Figure Number	Category comparison	Sample comparison	Test Performed	p-value	Significance level
**1D**	All	**Upf1-RIP vs Input**	Anova	<2E-16	***
	PTC vs SUT_XUT		Tukey_HSD	7,1299E-01	ns
	unknown vs PTC_pos			7,6615E-01	ns
	All others			<10E-7	***
**4B**	All	**Short poly(A)**	Anova	6,8500E-05	***
	Upf1 RIP-Input		Tukey_HSD	5,1440E-04	**
	Upf1 RIP-Tif4632 RIP			5,1500E-05	***
	upf1_del-Tif4632 RIP			3,5811E-03	**
	upf1_del-Upf1 RIP			9,3503E-03	**
	All	**Medium poly(A)**	Anova	4,8600E-02	*
	Tif4632 RIP-Input		Tukey_HSD	4,3383E-02	*
	All	**Long poly(A)**	Anova	4,1100E-05	***
	Tif4632 RIP-Input		Tukey_HSD	2,0115E-03	**
	Upf1 RIP-Input			5,2971E-03	**
	Upf1 RIP-Tif4632 RIP			2,5700E-05	***
	upf1_del-Tif4632 RIP			5,0510E-04	***
	upf1_del-Upf1 RIP			2,9504E-02	*
**4C**	All	**WT**	Anova	<2E-16	***
	SUT_XUT-PTC		Tukey_HSD	9,5797E-01	ns
	unknown-PTC_pos			4,8817E-01	ns
	noPTC vs noPTC_enr			6,0101E-01	ns
	All others			<10E-7	***
	All	**UPF1 RIP**	Anova	<2E-16	***
	SUT_XUT-PTC		Tukey_HSD	2,2596E-02	*
	unknown-PTC_pos			4,4085E-01	ns
	noPTC vs noPTC_enr			9,9406E-01	ns
	All others			<10E-7	***
**5C**	HHT2	**WT vs *upf1*Δ**	**T-test**	2,1240E-02	*
	RPL6B			1,1970E-02	*
	HCR1			2,3190E-02	*
	TDH3			1,1650E-02	*
	RPL28premRNA			9,3880E-02	ns
**6C**	All	**Short poly(A)**	Anova	4,6300E-04	***
	Input_Δupf2-Input		Tukey_HSD	5,4273E-03	**
	Input_Δupf3-Input			1,0341E-02	*
	RIP_Upf1-Input			2,9760E-04	***
	RIP_Upf1_Δupf2-RIP_Upf1			3,2167E-02	*
	RIP_Upf1_Δupf3-RIP_Upf1			8,0369E-03	**
	upf1del-RIP_Upf1			1,7161E-02	*
	**All others**			**>0,05**	ns
	All	**Medium poly(A)**	Anova	6,6700E-03	**
	RIP_Upf1_Δupf2-RIP_Upf1		Tukey_HSD	3,2395E-02	*
	RIP_Upf1_Δupf3-RIP_Upf1			7,9720E-03	**
	All	**Long poly(A)**	Anova	9,3100E-06	***
	Input_Δupf2-Input		Tukey_HSD	1,3080E-04	***
	Input_Δupf3-Input			2,2590E-04	***
	RIP_Upf1-Input			1,2700E-05	***
	RIP_Upf1_Δupf2-Input			2,7490E-04	***
	RIP_Upf1_Δupf3-Input			5,4340E-04	***
	upf1del-Input_Δupf2			6,0493E-03	**

	upf1del-Input_Δupf3			1,1267E-02	*
	upf1del-RIP_Upf1			3,9010E-04	***
	upf1del-RIP_Upf1_Δupf2			1,4044E-02	*
	upf1del-RIP_Upf1_Δupf3			2,9678E-02	*
**7B**	HHT2 cap	***upf1*Δ/WT**	**T-test**	1,1310E-02	*
	HHT2 5P			8,8636E-06	***
	RPL6B cap			7,3094E-03	**
	RPL6B 5P			8,0698E-04	***
	TDH3 cap			9,5475E-04	***
	TDH3 5P			4,6421E-02	*
	HHT2 cap	**dcp2-d/WT**		1,9099E-04	***
	HHT2 5P			4,9929E-03	**
	RPL6B cap			1,4029E-03	**
	RPL6B 5P			4,6316E-02	*
	TDH3 cap			8,1005E-03	***
	TDH3 5P			2,8036E-01	ns
**7C**	noPTC-noPTC_enr	**dcp2-d T=60'/ T=0**	**Anova**	<2E-16	***
	noPTC-PTC			<2E-16	***
	noPTC_enr-PTC			<2E-16	***
	noPTC-noPTC_enr	**not1-d T=60'/ T=0**		2,4305E-01	ns
	noPTC-PTC			1,3115E-01	ns
	noPTC_enr-PTC			8,0977E-01	ns
**S1A**	All	***upf1*Δ vs Input**	Anova	<2E-16	***
	unknown vs No_PTC_enr		Tukey_HSD	9,8548E-01	ns
	PTC_pos vs No_PTC_enr			4,0005E-03	***
	unknown vs PTC_pos			1,9600E-05	***
	No_PTC vs No_PTC_enr			1,0000E-07	***
	All other			<10E-7	***
**S1B**	All	**WT**	Anova	<2E-16	***
	PTC_pos vs PTC		Tukey_HSD	9,4426E-01	ns
	unknown vs PTC			6,2081E-01	ns
	unknown vs PTC_pos			7,6799E-01	ns
	No_PTC vs No_PTC_enr			9,8860E-01	ns
	All other			<10E-7	***
**S1C**	All	**WT**	Anova	<2E-16	***
	PTC_pos vs PTC		Tukey_HSD	1,0000E+00	ns
	unknown vs PTC			9,4381E-01	ns
	unknown vs PTC_pos			7,4113E-01	ns
	No_PTC vs No_PTC_enr			3,2704E-01	ns
	All other			<10E-7	***
**S4B**	All	**WT**	Anova	<2E-16	***
	SUT_XUT-PTC		Tukey_HSD	9,5797E-01	ns
	unknown-PTC_pos			4,8817E-01	ns
	noPTC_enr-noPTC			6,0101E-01	ns
	All other			<10E-7	***
	All	**Upf1_RIP**	Anova	<2E-16	***
	SUT_XUT-PTC		Tukey_HSD	2,2596E-02	*
	unknown-PTC_pos			4,4085E-01	ns
	noPTC_enr-noPTC			9,9406E-01	ns
	All other			<10E-7	***
	All	**upf1Δ**	Anova	1,3600E-11	***
	noPTC-PTC		Tukey_HSD	2,1361E-02	*
	noPTC_enr-PTC			2,6528E-02	*
	noPTC-PTC_pos			6,2000E-05	***
	NoPTC_enr-PTC_pos			7,2717E-03	**
	unknown-PTC_pos			2,1510E-02	*

	SUT_XUT-noPTC			5,4339E-03	**
	unknown-noPTC			<10E-7	***
	SUT_XUT-noPTC_enr			4,3674E-02	*
	unknown-noPTC_enr			5,1000E-06	***
	unknown-SUT_XUT			1,5113E-03	**
	all others			>0,05	ns
	All	**Tif4632-RIP**	Anova	<2E-16	***
	SUT_XUT-PTC_pos		Tukey_HSD	9,9984E-01	ns
	unknown-PTC_pos			2,9274E-02	*
	noPTC_enr-noPTC			9,9820E-01	ns
	unknown-SUT_XUT			6,1827E-02	ns
	unknown-noPTC_enr			3,7000E-05	***
	All other			<10E-7	***

ns	p > 0,05
*	p < 0,05
**	p < 0,01
***	p < 0,001

Supplementary Table 3
List of reagents and resources

REAGENT or RESOURCE	SOURCE	IDENTIFIER
**Antibodies**		
Sheep anti-digoxigenin-POD (poly), Fab fragments	Roche	Cat#11633716001
Mouse anti-FLAG M2 - HRP	Sigma-Aldrich	Cat#A8592
Rabbit Peroxidase Anti-Peroxidase soluble complex	Sigma-Aldrich	Cat#P1291
**Bacterial strains**		
NEB 10-beta competent E. coli	New England Biolabs	Cat#C3019H
**Chemicals, peptides, and recombinant proteins**		
Clarity Western ECL substrate	Bio-Rad	Cat#1705061
SsoAdvanced Universal SYBR Green Supermix	Bio-Rad	Cat#1725271
TruSeq Stranded mRNA sequencing kit	Illumina	Cat#20020594
NextSeq500 flowcell	Illumina	Cat#20024906
*BamH*I	New England Biolabs	Cat#R0136S
PstI	New England Biolabs	Cat#R0140S
Oligo d(T)$_{25}$ magnetic beads	New England Biolabs	Cat# S1419S
Q5 high-fidelity DNA polymerase	New England Biolabs	Cat#M0491
DNA polymerase I, Large (Klenow) Fragment	New England Biolabs	Cat#M0212S
T4 DNA ligase	New England Biolabs	Cat#M0202T
Direct RNA Sequencing Kit	Oxford Nanopore	Cat#SQK-RNA002
RNasin ribonuclease inhibitor	Promega	Cat#N2511
cOmplete protease inhibitor mix (no EDTA)	Roche	Cat#11873580001
DIG RNA labelling kit (SP6/T7)	Roche	Cat#11175025910
Digoxigenin-11-dUTP	Roche	Cat#11093088910
Indole-3-acetic acid (IAA)	Sigma-Aldrich	Cat#I2886
Beta-estradiol	Sigma-Aldrich	Cat#E2758
Doxycycline	Sigma-Aldrich	Cat#D3447

Rabbit IgG	Sigma-Aldrich	Cat#I5006
DNase TURBO	ThermoFisher	Cat#AM2238
Phusion high-fidelity DNA polymerase	ThermoFisher	Cat#F530L
Superscript II reverse transcriptase	ThermoFisher	Cat#18064022
Superscript III reverse transcriptase	ThermoFisher	Cat#18080044
Dynabeads M-270 epoxy	ThermoFisher	Cat#14302D
**Deposited data**		
UPF1_RIP, WT and *upf1Δ* Truseq Illumina sequencing	GEO	GSE283053
Upf1 CRAC (Illumina sequencing)	GEO	GSE299782
UPF1-RIP, WT input, *upf1Δ* eIF4G2-RIP, UPF1-RIP in *upf2Δ* (input and RIP) and UPF1-RIP in *upf3Δ* (input and RIP) Nanopore Direct RNA sequencing	GEO	GSE284490
R scripts for filtering Nanopore DRS reads and generating R figures	Zenodo	10.5281/zenodo.14615891
**Experimental models: Organisms/strains**		
LMA1667 - BY4741 *nam7(upf1)Δ*::KANMX6	[1]	N/A
LMA1669 - BY4741 *upf2Δ*::KANMX6	[1]	N/A
LMA1671 - BY4741 *upf3Δ*::KANMX6	[1]	N/A
LMA2154 (BY4741) (*S. cerevisiae*) *MATa ura3Δ0 his3Δ1 leu2Δ0 met15Δ0*	[2]	N/A
LMA2194 - BY4741 + NAM7*(UPF1)*::HIS3MX6	[3]	N/A
LMA3730 – BY4741 + NAM7*(UPF1)*-HTP	This study	N/A
LMA3314 – BY4741 + TIF4632-TAP::HIS3MX6	[3]	N/A
LMA3682 – PB1623 (S. pombe) + *nam7(upf1)*-CRAC	This study	N/A
**Oligonucleotides**		
PCR primers	This study	Table_Primers
**Recombinant DNA**		
pCM189-NFLAG-HIS3-100 p1640 (TETO7-NFLAG-HIS3-100) (URA3)	[4]	N/A
pCM189-NFLAG-HIS3-100-NoSTOP p1803 (TETO7-NFLAG-HIS3-100) (URA3)	This study	N/A
pCM189-NFLAG-HIS3-100-STOP+1+2 p1804 (TETO7-NFLAG-HIS3-100) (URA3)	This study	N/A
**Software and algorithms**		
ImageJ (1.54)	National Institutes of Health (NIH) https://imagej.net/ij/	N/A
Image Lab Software (6.1)	Bio-Rad	12012931
CFX Maestro Software (qPCR)	Bio-Rad	12013758
STAR (2.7)	[5]	N/A
Integrated Genome Viewer (IGV, 2.11)	[6]	N/A
R (3.8-4.4)	(R Core Team, 2024) https://www.R-project.org/	N/A
RStudio	https://posit.co/products/open-source/rstudio/	N/A
Guppy	Oxford Nanopore	N/A
Minimap2 (2.1)	[7]	N/A

SAMtools (1.9)	8	N/A
Nanopolish (0.13.2)	9	N/A
Rscripts were deposited on Zenodo (see Deposited Data)	Zenodo	10.5281/zenodo.14615891
**Other**		
ChemiDoc XRS+ imaging device	Bio-Rad	N/A
Trans-Blot Turbo transfer system	Bio-Rad	Cat#1704150
Supported nitrocellulose membrane	Bio-Rad	Cat#1620094
Amersham Hybond-N+	Cytiva Life Sciences	Cat#RPN303B
NextSeq 500	Illumina	N/A
Gel and PCR clean-up	Macherey-Nagel	Cat#740609.250
MinION sequencing device	Oxford Nanopore	N/A
GridION sequencing device	Oxford Nanopore	N/A
NuPAGE 4–12% Bis-Tris electrophoresis gel	ThermoFisher	Cat#NP0323BOX

Supplementary references

1. Giaever, G. & Nislow, C. The yeast deletion collection: a decade of functional genomics. *Genetics* 197, 451–465 (2014).
2. Brachmann, C. B. *et al.* Designer deletion strains derived from Saccharomyces cerevisiae S288C: a useful set of strains and plasmids for PCR-mediated gene disruption and other applications. *Yeast* 14, 115–132 (1998).
3. Ghaemmaghami, S. *et al.* Global analysis of protein expression in yeast. *Nature* 425, 737–741 (2003).
4. Audebert, L. *et al.* RNA degradation triggered by decapping is largely independent of initial deadenylation. *The EMBO Journal* 1–29 (2024) doi:10.1038/s44318-024-00250-x.
5. Dobin, A. *et al.* STAR: ultrafast universal RNA-seq aligner. *Bioinformatics* 29, 15–21 (2013).
6. Robinson, J. T. *et al.* Integrative genomics viewer. *Nat Biotechnol* 29, 24–26 (2011).
7. Li, H. Minimap2: pairwise alignment for nucleotide sequences. *Bioinformatics* 34, 3094–3100 (2018).
8. Danecek, P. *et al.* Twelve years of SAMtools and BCFtools. *Gigascience* 10, giab008 (2021).
9. Workman, R. E. *et al.* Nanopore native RNA sequencing of a human poly(A) transcriptome. *Nat Methods* 16, 1297–1305 (2019).
