# Supplementary material for: Nonsense-mediated mRNA decay factors target short poly(A)-tailed mRNAs lacking a premature termination codon
Source: Nat Commun. 2026 Apr 17;17:5333. doi: 10.1038/s41467-026-72132-1 (PMC13273070; doi:10.1038/s41467-026-72132-1)
Supplement: Supplementary file 2 — Description of Additional Supplementary Files [file 41467_2026_72132_MOESM2_ESM.docx]

Description of Additional Supplementary Files

File name: Supplementary Data 1

Description: List the yeast transcript features with coordinates and assigned categories. Excel file containing additional data, related to Figure 1

File name: Supplementary Data 2

Description: related to Figure 1, shows the normalized counts of Illumina sequencing data.

File name: Supplementary Data 3

Description: related to Figure 3, shows the normalized counts of Nanopore sequencing data.
