# Supplementary material for: Nonsense-mediated mRNA decay factors target short poly(A)-tailed mRNAs lacking a premature termination codon
Source: Nat Commun. 2026 Apr 17;17:5333. doi: 10.1038/s41467-026-72132-1 (PMC13273070; doi:10.1038/s41467-026-72132-1)
Supplement: Supplementary file 6 — Reporting Summary [file 41467_2026_72132_MOESM6_ESM.pdf]

nature portfolio

Corresponding author(s): Badis Gwenael

Last updated by author(s): Mar 24, 2026

Reporting Summary

Nature Portfolio wishes to improve the reproducibility of the work that we publish. This form provides structure for consistency and transparency in reporting. For further information on Nature Portfolio policies, see our Editorial Policies and the Editorial Policy Checklist.

Statistics

For all statistical analyses, confirm that the following items are present in the figure legend, table legend, main text, or Methods section.

n/a	Confirmed	
☐	☒	The exact sample size (*n*) for each experimental group/condition, given as a discrete number and unit of measurement
☐	☒	A statement on whether measurements were taken from distinct samples or whether the same sample was measured repeatedly
☐	☒	The statistical test(s) used AND whether they are one- or two-sided<br>*Only common tests should be described solely by name; describe more complex techniques in the Methods section.*
☒	☐	A description of all covariates tested
☐	☒	A description of any assumptions or corrections, such as tests of normality and adjustment for multiple comparisons
☐	☒	A full description of the statistical parameters including central tendency (e.g. means) or other basic estimates (e.g. regression coefficient) AND variation (e.g. standard deviation) or associated estimates of uncertainty (e.g. confidence intervals)
☐	☒	For null hypothesis testing, the test statistic (e.g. *F*, *t*, *r*) with confidence intervals, effect sizes, degrees of freedom and *P* value noted<br>*Give P values as exact values whenever suitable.*
☒	☐	For Bayesian analysis, information on the choice of priors and Markov chain Monte Carlo settings
☒	☐	For hierarchical and complex designs, identification of the appropriate level for tests and full reporting of outcomes
☐	☒	Estimates of effect sizes (e.g. Cohen's *d*, Pearson's *r*), indicating how they were calculated

Our web collection on statistics for biologists contains articles on many of the points above.

Software and code

Policy information about availability of computer code

Data collection	No software was used for data collection
Data analysis	Image Lab Software (6.1) Bio-Rad 12012931<br>CFX Maestro Software (qPCR) Bio-Rad 12013758<br>STAR (2.7.11b) (Dobin et al, 2013)<br>Integrated Genome Viewer (IGV, 2.11) (Robinson et al, 2011)<br>R (3.8-4.4) (R Core Team, 2024) https://www.R-project.org/<br>RStudio https://posit.co/products/open-source/rstudio/<br>Guppy Oxford Nanopore ont-guppy/4.5.2,<br>hdf5/1.10.6,<br>Minimap2 (2.1) (Li, 2018)<br>Samtools (1.9) (Danecek et al, 2021)<br>Nanopolish (0.13.2) (Workman et al, 2019)<br>Rscripts were deposited on Zenodo (see Deposited Data) Zenodo 10.5281/zenodo.14615891

For manuscripts utilizing custom algorithms or software that are central to the research but not yet described in published literature, software must be made available to editors and reviewers. We strongly encourage code deposition in a community repository (e.g. GitHub). See the Nature Portfolio guidelines for submitting code & software for further information.

Data

Policy information about availability of data

All manuscripts must include a data availability statement. This statement should provide the following information, where applicable:

- Accession codes, unique identifiers, or web links for publicly available datasets
- A description of any restrictions on data availability
- For clinical datasets or third party data, please ensure that the statement adheres to our policy

Sequencing data reported in this paper has been deposited at GEO: GSE283053 (Illumina RIP-seq), GSE299782 (CRAC) and GSE284490 (Nanopore) and all the sequencing experiments were performed with three biological replicates.

Research involving human participants, their data, or biological material

Policy information about studies with human participants or human data. See also policy information about sex, gender (identity/presentation), and sexual orientation and race, ethnicity and racism.

Reporting on sex and gender	not applicable
Reporting on race, ethnicity, or other socially relevant groupings	not applicable
Population characteristics	not applicable
Recruitment	not applicable
Ethics oversight	not applicable

Note that full information on the approval of the study protocol must also be provided in the manuscript.

Field-specific reporting

Please select the one below that is the best fit for your research. If you are not sure, read the appropriate sections before making your selection.

☒ Life sciences ☐ Behavioural & social sciences ☐ Ecological, evolutionary & environmental sciences

For a reference copy of the document with all sections, see nature.com/documents/nr-reporting-summary-flat.pdf

Life sciences study design

All studies must disclose on these points even when the disclosure is negative.

Sample size	At least three biological replicate of each condition were performed in all the experiments.
Data exclusions	Outliers reads with poly(A) tail length longer than 150 nt were removed from the analysis, since yeast poly(A) tails are not supposed to reach longer range.
Replication	All the experiments were replicated three time
Randomization	not applicable
Blinding	not applicable

Reporting for specific materials, systems and methods

We require information from authors about some types of materials, experimental systems and methods used in many studies. Here, indicate whether each material, system or method listed is relevant to your study. If you are not sure if a list item applies to your research, read the appropriate section before selecting a response.

Materials & experimental systems

n/a	Involved in the study	
☐	☒	Antibodies
☐	☒	Eukaryotic cell lines
☒	☐	Palaeontology and archaeology
☒	☐	Animals and other organisms
☒	☐	Clinical data
☒	☐	Dual use research of concern
☒	☐	Plants

Methods

n/a	Involved in the study	
☒	☐	ChIP-seq
☒	☐	Flow cytometry
☒	☐	MRI-based neuroimaging

Antibodies

Antibodies used	Rabbit Immunoglobulin G (IgG) - Sigma I5006-100mg, coupled to Invitrogen Dynabeads M-270 Epoxy size 300 mg ref Fisher Scientific 14302D<br>Rabbit Peroxidase Anti-Peroxidase soluble complex Sigma-Aldrich Cat#P1291
Validation	Western blot with known tagged protein.

Eukaryotic cell lines

Policy information about cell lines and Sex and Gender in Research

Cell line source(s)	Only Saccharomyces cerevisiae yeast strains originating from the BY4741 reference strain:<br>LMA1667 - BY4741 nam7(upf1)Δ::KANMX6 (Giaever et al, 2002)<br>LMA2154 (BY4741) (S. cerevisiae) MATa ura3Δ0 his3Δ1 leu2Δ0 met15Δ0 (Brachmann et al, 1998)<br>LMA2194 - BY4741 + NAM7(UPF1)::HIS3MX6 (Ghaemmaghami et al, 2003)<br>LMA3730 – BY4741 + NAM7(UPF1)-HTP This study<br>LMA3314 – BY4741 + TIF4632-TAP::HIS3MX6 (Ghaemmaghami et al, 2003)<br>LMA3682 – PB1623 (S. pombe) + nam7(upf1)-CRAC This study
Authentication	Each mutation introduced in a yeast strain was PCR verified and sequenced, plus western blot verified (when a tag with available antibody was available).
Mycoplasma contamination	not applicable
Commonly misidentified lines<br>(See ICLAC register)	not applicable

Plants

Seed stocks	not applicable
Novel plant genotypes	not applicable
Authentication	not applicable
