# Supplementary material for: Nonsense-mediated mRNA decay factors target short poly(A)-tailed mRNAs lacking a premature termination codon
Source: Nat Commun. 2026 Apr 17;17:5333. doi: 10.1038/s41467-026-72132-1 (PMC13273070; doi:10.1038/s41467-026-72132-1)
Supplement: Supplementary file 7 — Transparent Peer Review file [file 41467_2026_72132_MOESM7_ESM.pdf]

nature portfolio

Peer Review File

Nonsense-Mediated mRNA Decay factors target short poly(A)-tailed mRNAs lacking a premature termination codon

Corresponding Author: Dr Gwenael Badis-Breard

This file contains all reviewer reports in order by version, followed by all author rebuttals in order by version.

Version 0:

Reviewer comments:

Reviewer #1

(Remarks to the Author)
Manuscript by Gouhier et al., examines the collection of Upf1 targets on a transcriptome-wide scale. Authors purify RNAs associating with Upf1 and employ both Illumina and nanopore sequencing to evaluate mRNA isoform enrichment. The techniques employed are adequate to the question, and thus the paper would add a quite substantial RNA-seq RIP dataset, along with steady state datasets of Upf1/2/3 single delta strains. As expected transcripts containing premature codon stop (PTC) emerge as targets (mostly non-coding SUTs/XUTs and specific mRNAs). Such types of targets were previously numerously examined using reporter transcripts. What is surprising is that the authors propose that a subfraction of mRNAs containing short pA-tails is also subjected to Upf1-mediated nonsense mediated decay. This is based on Illumina-RIPseq and on a general accumulation of short tailed mRNAs in upf1-del, upf2del, upf3del. This is the novelty, which is notable to a broader audience of Nature Communications. Overall I think that the data itself looks sound, but the depth/presentation of the data is somehow convoluted, at least to me, and perhaps relying too heavily on the Illumina-RIPseq.

Major:

1. the Upf1-RIP-Illumina dataset is used to define enrichment of Upf1 targets. Next the Upf1-RIP-Nanopore dataset is used to establish pA-tail profiles. However, fold-change enrichment of RNAs in both datasets appear quite different (Fig. 2D), despite the statement in the text. This is only 'my biased reviewer eyes', and perhaps I am mistaken. Therefore I think that the authors should provide a Pearson/Spearman correlation coefficient to support their statement. If it turns out low they should justify why. That said, the nanpore datasets tend to have lower read yeild (at least in the old chemistry, is it the case here?) so I don't really think they should be used to determine enrichment at all. Hence, I recommend to ammend this figure and move it to the supplement.

2. Authors interpret the pA-tail profiles of steady-state total upf1/2/3 delta strains through the results of the Upf1-RIP. I do think that in the absence of spike-ins/proper normalization of the RIP (by background eg. ncRNA sno/sn?; might be difficult) its value should be treated as limited. Justification:

Model states that Upf1 is recruited to short-tailed mRNAs because there is no Pab1 on the tail. upf1/2/3 deltas do accumulate short-tailed mRNAs globally (Fig. 6), so this fits the model. But the UPF1-RIP noPTC_enr and noPTC (not enriched) mRNAs both accumulate short-tailed mRNAs to the same extent (Fig. 3C). So it seems that the Upf1-bound and -unbound mRNAs react the same way, which argues with the model. Are you really sure that the Upf1-RIP-Illumina data reliably determines the set of targets? Perhaps a CRAC experiment to complement this would be better? Nobody will expect such a heavy experiment in revision. I would just ask to reconsider the structure of the publication.

3. paper-structure-wise: Fig. 3 should go before Fig. 2 (global analysis and then single mRNA examples). Fig. 6 is the most valuable in my view (especially panel 6B). Perhaps targets should be first defined by selecting mRNAs that have the most altered pA-tail profiles, which would then be compared with the RIP-seq datasets (both Illumina and nanopore).

4. a more in depth global analysis of extra features of Upf1 mRNA targets would be appreciated. I do like the single mRNA examples in Fig. 2 but I wonder how general they are?

5. What is the correlation between pA-tail length and RNA abundance change in upf1, 2 and 3 strains?

6. There is no login for reviewers to check the RNA-seq raw data submissions. Only accession numbers(GSE284490, GSE283053) are given. GEO informs that such datasets exist but are currently private. Therefore the Reviewer cannot confirm deposition of the raw data (in accordance with journal requirements).

Minor:

1. somehow define the identity of upf1 targets (selected from RIP or total). Are they abundant mRNAs? Long-short-tailed by nature? any functional groups?

2. Fig. 1C -> please add one column marking what percentage each group represents as transcriptomic ORF/ncRNA total. Specify which groups are coding.

3. Fig. 2C -> pA-tail profiles for single mRNA examples. Please transfer pA-tail profiles from supplement S2A to main 2C. I think comparison of WT, upf1del totals to Upf1-RIP is the only correct way to show the data. It is important that in upf1del the overall profile shifts towards shorter pA-tails, and that the Upf1-RIP fraction displays even shorter pA-tails. I am missing a more global analysis for non-PTC_enriched groups that would support this for a larger number of single mRNAs. Single gene examples are a support but no definitive proof.

4. Fig. 5 -> the pA+-selection experiment. Here, different mRNAs are examined compared to Fig. 2. I wonder whether in the various pA- and pA+ fraction authors could differentiate for various isoforms given as examples in Fig. 2? Perhaps by adding a RNaseA digestion coupled to trimming the 3'UTR at the beginning to facilitate length differentiation using a 3'UTR probe?

5. Please improve the description of Fig. 1B (MA plot, please write on x-axis the same description as in the legend).

6. Fig. 2B -> comparison of RIP-Illumina and RIP-nanopore reads. Please draw a scheme of the genomic locus with ORF and pre-mRNA cleavage sites marked. Can you clearly distinguish two cleavage sites based on the sequencing data?

7. Fig. 2D -> Please introduce a diagonale. It will make the graph easier to read.

8. The paragraph concerning Fig. 3A is written in a rather convoluted manner, it is not clear when the authors refer to input (total) sample and when to RIP samples. Please improve.

9. Page 6 line 5 -> useless '_' ('Fig.2A)._These')

(Remarks on code availability)
code submission embargoed until end dec. 2025

Reviewer #2

(Remarks to the Author)
Gouhier and colleagues applied here RIP-seq (Illumina-based) and Oxford nanopore direct RNA sequencing (ONT-DRS) to identify Upf1p-bound transcripts and assess their poly(A) tail length. The RNAs identified in the RIP-seq were separated into different categories. Classical NMD targets like SUTs/CUTs and PTC-containing mRNAs were enriched as expected. Interestingly, approx. 15% of mRNAs without an apparent PTC were < 2-fold enriched in the Upf1p-IP (designated noPTC enr) but not upregulated in upf1 cells. The authors then focused on this population of stable RNAs that are associated with Upf1p yet not degraded by NMD.
Next, they performed ONT-DRS on the input, Upf1p-bound, and eIF4G-bound RNA samples in WT and upf1 cells. Interestingly, transcripts (both PTC and noPTC enr) with short poly(A) tails (0-25 As) were strongly enriched among the Upf1p-bound transcripts, while the eIF4G-bound sample enriched transcripts with medium (>25 – 50 As) and long (>51 As) poly(A) tails, suggesting that many of the Upf1p-bound transcripts might represent decapped decay intermediates. Given that NMD was reported to occur independently of deadenylation, the enrichment of RNAs with short poly(A) tails is unexpected and seemingly contradicts a previous publication of the authors, in which they reported that PTC-containing transcripts on average contained longer poly(A) tails than other transcripts (Audebert et al., 2024).
The authors then went on to present data from a polysome gradient experiment (Fig. 4) of which I can neither judge the data quality nor understand what the authors‘ conclusion is from this data. Moreover, the use of a histone mRNA (HHT2), which in mammalian cells is known to bind UPF1 for its induced rapid decay at the S phase (cell cycle-dependent histone mRNA decay), is questionable. The authors should therefore test at least one, better two other, more representative mRNAs of the noPTC enr category. Since the data in Fig. 4 does not strengthen the overall conclusions of the manuscript, and at the same time raises criticism regarding the quality of the experiment, I suggest to remove it from the manuscript.
Using a biochemical approach to experimentally separate RNPs into three fractions with (i) no or short (S), (ii) medium (M), and (iii) long (L) poly(A) tails, the authors next assessed the distribution of HHT2 (noPTC enr transcript) and pre-RPL28 (PTC transcript) RNAs from WT and upf1 yeast in this poly(A) length fractions. Most of the NMD-targeted transcript (pre-RPL28) is present in the L fraction in WT cells and shifts towards M and S fractions in upf1 cells, confirming previous observations that NMD usually occurs on transcripts with long poly(A) tails. Compared to WT cells, HHT2 mRNA was

enriched in the S fraction in the upf1 cells, consistent with the hypothesis that as in mammals, Upf1 might be required for the cell cycle-dependent histone mRNA decay also in yeast. Cell-cycle-dependent histone mRNA decay is not well understood in yeast and whether Upf1 plays a role is unclear. Therefore, the data here is potentially highly interesting, but the authors do not discuss this aspect. Since TDH3, a noPTC mRNA behaved similarly to HHT2, one could also argue that the previously described role for mammalian UPF1 in histone mRNA decay is only the tip of the iceberg and that Upf1 is generally involved in degrading deadenylated mRNAs with only a few As left, which is the interpretation of the authors.
To further investigate the effect of Upf1p on the stability of normal, not NMD-targeted mRNA, the author assessed the half-life of a poly(A) length-fractionated tet-off regulated HIS3 reporter RNA. They found that in upf1 cells, the half-life of the HIS3 RNA with a short or no poly(A) tail (fraction S) was strongly increased, while little and no stabilisation was observed in the M and L fractions, respectively. This data elegantly and compellingly confirms that Upf1 is required in selectively targeting transcripts with short or no poly(A) tails for degradation. It would be really nice if for comparison and completeness, the same experiment were also done with an NMD-targeted reporter RNA.
Finally, to address if other NMD factors are also required for the degradation of short poly(A)-tailed noPTC mRNAs, the authors measured the distribution of poly(A) tail length by ONT-DRS in input and Upf1-RIP samples from WT, upf2 , and upf3 strains. Upf2p and Upf3p depletion increased the fraction of short poly(A)-tailed RNAs in the input samples even more than upf1 (compare Fig. 3A with 6B), but in the Upf1-RIP samples, more RNA with short poly(A) tails is pulled down in WT cells than in upf2 , and upf3 cells. While the latter observation remains uncommented by the authors (see my comment below), the input data suggests that not only Upf1p but also Upf2p and Upf3p (i.e. NMD) are involved in the degradation of noPTC mRNAs with short poly(A) tails. The finding that in the absence of Upf2p or Upf3p, Upf1p-assocaited mRNAs have longer (medium) poly(A) tails does not fit in the proposed model (Fig. 7), should be discussed, and the model adjusted.

Overall, this study of the poly(A) tail lengths of Upf1p-associated mRNAs with ONT-DRS is original and has provided interesting and surprising findings. While the polysome fractionation experiment cannot be assessed, the rest of the data appear to be of high quality and convincing. Apart from some points made above and below, the authors carefully and thoughtfully interpreted the data and provide evidence for a novel function of Upf1p in the degradation of deadenylated RNAs with short or no poly(A) tails remaining. Whether Upf2p and Upf3p, and hence NMD, are also involved is not fully supported by the presented data. In my opinion, the role of Upf1p in the decapping of these short poly(A)-tailed RNAs would be worthwhile exploring, as this is likely the degradation route of these RNA species. Without clarification of this point, the presented model (Fig. 7), although appealing, remains highly speculative. Moreover, as discussed by the authors, the model predicts that Upf1p-bound short poly(A)-tailed mRNAs should lack Pab1 association and vice versa, a prediction that could and should be tested.

Additional points:
Double-check references: some references in the introduction seem not to fit. For example, ref 9 does not describe the faux 3 ´UTR model, instead it refers to the very first description of the NMD phenomenon. Also, the sentence on p.2, lines 5-7 needs one or more refs.

p.2, lines 24-27: For clarity, it should be mentioned that the cited studies pertain to mammalian cells, where UPF1 phosphorylation rather than its association with RNA appears to be the distinctive marker for NMD, whereas in yeast, extensive Upf1p phosphorylation seems not to occur and it is therefore proposed that Upf1p binding to RNA identifies NMD-targeted transcripts.

p.2, lines 35-37: Please explain how you arrived from the numbers in Fig. 1C. to the slightly smaller numbers indicated in Fig. 1D.

Fig. 2D: While fewer Upf1p-associated transcripts were identified with ONT-DRS than with Illumina, which is consistent with the expected lower sequencing depth, you curiously detected now 107 Upf1p-enriched noPTC transcripts. What is the explanation for this? Were they all close to the threshold and just represent the variability in the assay?

p4., line 38: There is no transition from the previous section (describing Fig. 2) to this section (describing Fig. 3). Please start this section by explaining the rationale for doing these experiments; otherwise it is difficult to follow.

Fig. 3A: Since the y-axis is missing in this plot, it is not possible to compare the heights of the peaks and shoulders among the four samples.

Fig. 3, legend: The definitions for short, medium, and long poly(A) tails differ slightly between the figure legend and the Results section. Please use a consistent definition.

Fig 3D: It is unclear from the figure legend and the Results section (p. 5, line 41 and below) whether the indicated proportion of reads in each category refers to the total sequenced RNAs or only to those with short poly(A) tails. Please clarify.

Fig. 4A: The polysome gradient data as presented is difficult to assess and interpret. Instead of showing 25S and 18S rRNA levels, the authors should show the A260nm trace, as is common practice to show polysome data. This would allow judging the quality of the gradient. It looks suspicious that no 18S rRNA is present in fractions 7-10 (labeled as free 40S). Also the pattern of band intensities for HHT2 and rRNA looks strangly different between the fractions in the upf1 sample. Do the authors have an explanation for why there is much more HHT2 mRNA in fractions 19-20 and 25-26 than in the other fractions, and more 25S and 18S rRNA in fractions 15-16, 21-22, and 25-26 than in the other fractions?

p.6, lines 22-25: Even though a reference is given, a brief description of the ePAT method would facilitate understanding of what is shown in Fig. 4B. Also, the abbreviation TVN is not explained.

Suppl. Fig. 4B: While the Upf1p enrichment values between the three reporter RNAs are very similar, the levels differ significantly. Do the authors have an explanation for this? Could it be that the no Stop and the Stop +1+2 constructs are less stable due to unintentionally inserted less optimal codons?

Fig. 6C: Please discuss why the fraction of short poly(A)-tailed RNA is lower in the Upf1p-RIP samples of the upf2 and upf3 strains compared to the WT strain. Could this indicate that in the absence of Upf2p or Upf3p, Upf1p has a lower affinity for its RNA targets?

Fig. 6D: The Upf1p-RIP data on the two noPTC transcripts HHT2 and TDH3 show no enrichment of the short poly(A) tail fraction in the upf2 and upf3 strains, the majority of these two Upf1p-associated mRNAs have a medium long poly(A) tail. In my view, that contradicts the conclusion that the NMD machinery has a critical role in the degradation of short poly(A)-tailed noPTC mRNAs (p. 8, lines 34/35). Rather, it suggests that when NMD is inactivated by depletion of Upf2 or Upf3, the Upf1p-assocaited mRNAs cannot be deadenylated to the same extent than in cells with active NMD. The authors cannot ignore this observation and should discuss possible interpretations and how this affects their proposed model.

(Remarks on code availability)
as stated above, this is not my area of expertise.

Reviewer #3

(Remarks to the Author)
Nonsense-mediated decay mainly targets short poly(A) tailed mRNAs lacking a premature termination codon

Gouhier et al.

Summary

In this work, short read Illumina sequencing of RNA associated with immunopurified yeast Upf1 (Upf1-RIP) revealed strong enrichment of Upf1 association with canonical NMD substrates – including PTC-containing mRNAs, Xrn1-dependent unstable transcripts (XUTs), and stable unannotated transcripts (SUTs). Additionally, a modest enrichment of transcripts lacking a PTCs ("noPTC") was also identified. Notably, however, only a small subset of noPTC mRNAs (373 out of 2254, or 16%) were found to be enriched in Upf1 binding. To better understand the class of noPTC mRNA bound to Upf1, Nanopore direct RNA sequencing was applied to provide information on both RNA poly(A) tail length and to distinguish isoforms of RNA expressed from individual genes. Based on this analysis, Upf1 was found to associate primarily with RNAs harboring short (0-25As) and medium (25-50As) poly(A) tails, but not RNAs with long (>51As) poly(A) tails, which, curiously, are a typical feature of PTC-containing transcripts undergoing targeting by NMD in yeast. Additionally, the majority of Upf1-associated transcripts identified by Nanopore sequencing lacked a PTC. The authors then showed that Hht2 mRNA, a representative noPTC mRNA enriched for Upf1 association, was polysome associated even when harboring a short poly(A) tail, and that in the absence of NMD (i.e. a upf1 deletion yeast strain), polysome-associated Hht2 mRNA had even shorter poly(A) tail lengths. The increase in abundance of Hht2 mRNA harboring short poly(A) tails in the absence of NMD was further confirmed using oligo-d(T) fractionation. Notably, the same trend (increased abundance of short poly(A) tailed transcripts in the absence of NMD) was observed for Tdh3 mRNA, which was not found associated with Upf1 in RIP assays. Using an inducible, codon-optimized HIS3 reporter, the authors attempt to measure mRNA half-life for transcript populations with short, medium and long poly(A) tails, and suggest that in the absence of NMD, short poly(A) tailed His3 mRNA is stabilized. Finally, the authors provide additional Nanopore data indicating that Upf1 association with short poly(A) tailed transcripts is dependent upon Upf2 and Upf3, its cofactors in NMD.

Based on their data, the authors propose a model by which Upf1 is recruited to mRNAs harboring short poly(A) tails and targets them to NMD, which, if true, would represent a new class of NMD substrates. Their model relies heavily on the validity of the faux 3'UTR model (proposed in Amrani et al., 2004) which posits that Upf1 is recruited to terminating ribosomes when Pab1 is unable to promote efficient translation termination through its interaction with release factor eRF3 (due to the extended distance between Pab1 and ribosomes terminating at a PTC or, as proposed in this study, for short poly(A) tailed mRNAs that are predicted to lose Pab1 binding capacity).

While provocative, direct testing of the model is glaringly lacking and data that appears to directly contradict the model is ignored. Moreover, there is sufficient published literature that questions how Upf1 is recruited to mRNA, that Upf1 association with an mRNA does not a priori lead to targeting of a transcript to NMD (i.e. binding does not equal targeting), and the role Pab1 plays in NMD substrate recognition (see below), that undermine the faux 3'UTR model itself. Critically, the authors do not show a directly link between mRNAs harboring short poly(A) tails, loss of Pab1 from the transcript, inefficient translation termination, Upf1 recruitment, and decay of the transcript through NMD-mediated mRNA decapping. Curiously, they do not address why Upf1 is not found to bind to all mRNAs with short poly(A) transcripts (i.e. from all genes) or (on the flip side) why only 15% of noPTC mRNAs are bound by Upf1. One simple interpretation for these data is that Upf1 samples all transcripts, and that it disproportionally binds a subset of stable, highly abundant, deadenylated transcripts (lacking a PTC). The observation that short poly(A) tailed mRNAs accumulate and turn over more slowly in NMD mutants could simply be a consequence of a change in kinetics of mRNA decapping (the rate limiting step in decay in yeast) in wild-type cells versus cells with a completely different transcriptome due to inactivation of NMD. Indeed, in the absence of NMD, both direct

and indirect NMD substrates would accumulate and compete for the decapping machinery and thus accumulate with short poly(A) tails.

General Comments:

1. In several instances, the data fails to support the proposed model.
a. Upf1 shows similar levels of association (by RIP) with both short poly(A)-tailed transcripts and medium poly(A)-tailed transcripts (Figs. 3A, 3C). According to the authors, medium poly(A)-tailed transcripts should still harbor long enough tails to bind Pab1 and, thus, Upf1 would not be predicted to be recruited to those transcripts.
b. Tdh3 mRNA, the representative noPTC, short poly(A) transcript that is not found to be bound by NMD also appears also to be stabilized in the absence of NMD. This suggest that the changes in abundance of these mRNAs in wild-type versus NMD mutants is independent of Upf1 association.
c. Figure 3C indicates that not all short poly(A)-tailed mRNAs are Upf1-bound/enriched, indicating that a short poly(A) tail is not the sole determinant of recruiting/binding Upf1.
d. It is unclear why only 16% of no_PTC mRNA are bound by Upf1 and the other 84% are not. Are their features of this subset that might provide an alternative explain for why Upf1 enrichment on them?

2. The authors fail to reference published literature particularly relevant to this work, including that which identified a role for accelerated deadenylation in the degradation of NMD substrates (Cao and Parker, 2003; Mitchell and Tollervey, 2003), Upf1 binding to mRNA independent of translation (Hurt et al., 2013; Zund et al., 2013), and data contradicting a role for Pab1 in NMD (Roque et al., 2014; Meaux et al., 2008).

3. It is unclear why PTC-containing mRNA harboring long poly(A) tails was not found in Upf1 RIPs by Nanopore sequencing. One explanation is that this population of transcripts is rapidly degraded by NMD and depleted from the population. If true, then it is unclear how short poly(A) tailed mRNAs that are purportedly targeted to NMD are readily found bound by Upf1 here.

4. The authors equate Upf1 binding of an mRNA with its targeting to NMD. There are numerous examples in the literature of Upf1 binding NMD in-sensitive mRNAs and also that Upf1 binding does not a priori lead to targeting the mRNA to NMD (Hogg and Goff, 2010).

Specific Comments:

1. Is it unclear in Figure 2 if the Nanopore sequencing data includes data for full length mRNA or whether decay fragments are included. This is relevant as decay intermediates would be decapped and expected to have shorter poly(A) tails (skewing the data), and that data is compared to mRNA bound by eIF4G that, by default, is capped and full length. This needs to be clarified and only full-length transcripts reported in the analysis.

2. It is unclear why the Nanopore sequencing detects a greater proportion of Upf1-bound noPTC mRNA (some of which were not observed in Illumina-prepared sequencing libraries). Is it related to #1 above; the authors need to address.
3. It is unclear from the text where the transcripts per category in Fig. 1C come from and how the Upf1-bound PTC-containing dataset compares to those in the literature. Also, the numbers do not appear to match with the number of transcripts per category in Fig. 1D.

4. Fig. 3D does not report any information related to mRNA poly(A) tail length, yet the text makes reference specifically to short poly(A) tailed transcripts when this figure is discussed.

5. The relevance for the inclusion of data on mRNA codon optimality (which influences translation elongation) and polysome association to support their model is unclear. Additionally, optimal codon content is only marginally different between PTC and noPTC transcripts (Supp Fig. 1) and there is no discussion of differences between optimal codon content between Upf1-bound and unbound transcript pools. Finally, data in Fig. 4A are difficult to interpret due to lack of quantification and do not support the suggested role for Upf1 in the “redistribution of mRNPs containing short poly(A)-tailed mRNAs, suggesting a role of NMD in their remodeling or translation.”

(Remarks on code availability)

Version 1:

Reviewer comments:

Reviewer #1

(Remarks to the Author)
The manuscript by Gouhier et al. examines Upf1 targets, adding to the well-characterized PTC-containing transcripts, also non-PTC mRNAs bearing overly short pA-tails. The manuscripts employs transcriptome prolifling using Illumina and nanopore sequencing on both total, and Upf1-immunoprecipitated RNAs. During revision the authors have put much effort in both improving the clarity of data presentation, and by adding novel data, including CRAC-profiling of Upf1 binding sites. Extending the role of Upf1 beyond decay of PTC transcriptipts into decay of normal mRNAs is a novelty, which will draw

attention to the manuscript from the RNA community. The main hypothesis concerning Upf1 recruitment to those novel targets still remains speculative (specifically the Pab1 role). However, since this novel class of targets is so much better described following revision, it is clear to me that the manuscript itself will the basis of follow-up research. In conclusion, all my concerns were properly addressed, and I would encourage publication of the draft.

(Remarks on code availability)

Reviewer #2

(Remarks to the Author)
Gouhier, Saveanu, and Badis present here a substantially revised version of their manuscripts. As was evident from the many points raised by all three reviewers, the original version was difficult to read and raised many questions, mainly regarding the interpretation of the presented data.
Also, the long replies of the authors to the reviewers' points did not clarify the original concerns, at least not for me. In fact, on certain points, I got even more confused than before, and new questions arose. Hence, I had no choice but to evaluate the entire revised version again as if it were a new, original submission. Below are my comments:

Using a combination of RNA-seq and RIP-seq, the authors aimed at shedding light on the rules governing transcript recognition by Upf1 in yeast. While the majority of Upf1-associated RNAs were known NMD targets (XUTs/SUTs, and PTC-containing mRNAs), a minor fraction of the mRNAs with no obvious PTC (termed "noPTC") was also enriched in the Upf1 RIP-seq data (345; 16% of all noPTC transcripts). Given these numbers, the title of the manuscript is misleading, because it states that this minor fraction of transcripts represents the main targets of NMD, while the authors' data (Fig. 1) clearly show that the previously characterized PTC-containing transcripts (categories XUT/SUT and PTC) are much more abundant among the Upf1-associated RNAs. Furthermore, the enrichment values (Upf1-associated / Input) of transcripts belonging to this "noPTC_enr" transcript category are much lower than the corresponding values for classical PTC-containing transcripts (Fig 1e). While it is curious and worthwhile to investigate why some PTC-lacking transcripts are also enriched in the Upf1 RIP, the authors do not answer this question. The noPTC_enr transcripts appear to interact on average more with Upf1 in the 3´UTR than noPTC non-enriched transcripts, but the authors did not further analyze which features in these 3´UTRs cause enhanced binding of Upf1.
Instead, the authors analyzed by DRS the poly(A) tail length distribution of transcripts IPed with Upf1. The authors found that Upf1-associated NMD-sensitive transcripts had longer poly(A) tails than Upf1-associated NMD-insensitive transcripts, consistent with previous papers showing that NMD initiates degradation of yeast mRNAs by direct decapping without the need for prior deadenylation. However, on the specific example shown in Fig. 3 (RPL22B vs pre-RPL22B), this difference was quite small (33 vs 38 As). In general, Upf1-associated transcripts appear to exist in two overlapping populations: one population with short (<25; average 15) and one population with medium (average 30) long poly(A) tails (Fig 4a). But as already mentioned in my previous review, the representation of the data in Fig. 4a is problematic, because the y-axis is lacking, which prevents comparisons across the different sample: For example, when comparing upf1Δ with WT, it is unclear if the peak in the short poly(A) bin increased in upf1Δ, or if the peak in the medium poly(A) bin increased in WT, or if both changed in the absence of Upf1. Compared to the overall distribution of poly (A) tail lengths in the input of WT cells, transcripts with short poly(A) tails are substantially enriched in the Upf1 RIP sample. The authors show that, in particular, noPTC and noPTC_enr transcripts are 2 – 2.5-fold enriched in the Upf1 IP among the transcripts with short poly(A). Overall, these data indicate that Upf1 binds rather indiscriminately to PTC and noPTC transcripts (consistently with data from mammalian cells) and that a substantial proportion of the Upf1-bound noPTC transcripts has short poly(A) tails.
Applying ePAT on poly(A) length fractionated RNA samples showed that the levels of both, the noPTC HHT2 and the classical NMD target RPL28 pre-mRNA, increased in upf1Δ yeast, indicating that also the short poly(A)-tailed noPTC transcripts are degraded in a Upf1-dependent manner (Fig. 5). This was confirmed by measuring the stability of a codon-optimized HIS3 reporter RNA (HIS3-opt). Until here, I think the data and interpretations are solid.
Next, the authors conducted DRS and analyzed the poly(A)-tail length distribution in upf2Δ and upf3Δ strains (Fig. 6). If the proportion of Upf1-associated short poly(A)-tailed transcripts decrease in upf2Δ and upf3Δ strains, this would either suggest that in the absence of Upf2 and Upf3, they are more efficiently degraded, or that their deadenylation is slowed down and therefore a correspondingly larger fraction of these reads is now found in the medium and long poly(A) categories. Fig. 6d suggests the latter. Therefore, in my view, the data do not support the conclusion that Upf2 and Upf3 play a critical role in the degradation of short poly(A)-tailed noPTC mRNAs (lines 402-403). Rather, the data suggest to me that their absence prevents the fast deadenylation of the Upf1-associated transcripts.

Finally, in an elegant approach that allows the distinction between capped full-length RNAs and RNA degradation intermediates with a 5´ phosphate, the authors show that depletion of Upf1 increases the proportion of capped HHT2, RPL6B, and TDH3 transcripts and decreases the proportion of decay intermediates of these three genes, similar to inactivation of Dcp2. This indicates that Upf1-binding to these three mRNAs promotes their decapping.

My three main points that should be resolved before publication of this manuscript are the following:

1.) The strong focus on the categorization of the Upf1-immunoprecipitated RNAs, in particular the distinction between noPTC_enr and noPTC in the beginning of the manuscript, confuses and distracts from the main finding of the manuscript, namely that Upf1 is found associated with many mRNAs that are not NMD-sensitive after they have been almost completely deadenylated, and that Upf1 promotes decapping of these RNAs.
2.) A weakness remains that no explanation could be found for why some noPTC transcripts bind Upf1 better than others. In some assays, TDH3 has been included as a representative transcript of the category that is not enriched in the Upf1 RIP (yet

still is bound by Upf1). It would be more informative if one would test transcriptome-wide for a correlation between Upf1-association and the effect on decapping of RNAs, rather than pick one or two specific transcripts of the different categories.
3.) In my opinion, the poly(A) tail profiles in Fig. 6 do not support the conclusion that Upf2 and Upf3, and hence NMD, are involved in promoting decapping of RNAs with short poly(A) tails. This would require additional evidence, for example, including upf2Δ and upf3Δ strains in the analysis shown in Fig. 7. With the currently provided data, the title of the manuscript is inappropriate and misleading. A more appropriate title would be “Upf1 promotes decapping on short poly(A)-tailed mRNAs lacking a premature stop codon”. Without compelling data that the observed Upf1-promoted decapping of short poly(A)-tailed noPTC transcripts is also dependent on Upf2 and Upf3, the model proposed in Fig. 8 and the entire Discussion remain highly speculative.

(Remarks on code availability)

Reviewer #3

(Remarks to the Author)
The manuscript has improved from the first submission and the data indicating Upf1 binds mRNA lacking at PTC is convincing. However, this reviewer remains unconvinced that the data supports a conclusion that Upf1 is specifically recruited to short poly(A) tail mRNAs and that these transcripts are targeted to NMD.

1. The authors claim “a low immunoprecipitation background” (line 110) in the manuscript based on almost undetectable mitochondrial-encoded mRNAs in their Upf1-IP but suggest in their rebuttal that co-purification of Upf1 with medium poly(A) transcripts is due to background. Figure 4A and B indicates that Upf1 copurifies almost equal amounts of short and medium poly(A) tail transcripts, which, if due to background contamination, puts the entire analysis of Upf1-associated RNA in question. Notably, in Fig. 3, Upf1 is shown to bind RPL22B transcripts (lacking a PTC) with an average tail length of 33 nucleotides – which would place it in the ‘medium’ length poly(A) tail category (and which would be expected to be bound by Pab1).
2. The model that NMD targets short poly(A) tailed transcripts rests on the assumption that Pab1 association is lost for transcripts with poly(A) tails shorter than 30 and that this leads to inefficient translation termination (and recruitment of the NMD machinery). Neither of these assumptions are tested here or experimentally supported. Although the authors draw attention to a previously published observation that short HHT2 mRNA is not co-purified with Pab1, this is insufficient to suggest that all short poly(A) transcripts are void of Pab1. Moreover, published biochemical and CLIP analyses of Pab1 suggests that it can efficiently bind A-rich sequences 12 nucleotides in length (although the protein covers 25-30 nucleotides).
3. The author’s interpretation of Meaux et al (2008) is that NMD occurs in the absence of Pab1; however, a key observation is that a no-PTC transcript completely lacking a poly(A) tail (due to ribozyme cleavage) is not targeted to NMD, which would be expected if their model was correct.
4. The proposed model (Fig. 8) indicates that Upf1 (and the other NMD factors) is recruited to the terminating ribosome of short poly(A) tailed transcripts (via interaction with eRF1), however, their own CRAC data (Fig. 2) maps Upf1 along the 3’ UTR of these transcripts; it is unclear how inefficient termination can lead to 3’ UTR binding by Upf1 – this needs to be reconciled.
5. The authors should assay the unbound fraction to determine the abundance of short poly(A) tailed mRNAs that fail to associate with Upf1 and explain how these transcripts escape recruitment of Upf1 and targeting to NMD under the current model.
6. The authors can not rule out that inhibition of decapping (and disappearance) of short poly(A) mRNA in strains lacking NMD activity is indirect. Indeed, in upf deletion strains, prior NMD substrates are now stable, abundant within the transcriptome, and are substrates for deadenylation-dependent decapping. The flood of additional substrates for normal mRNA turnover would be expected to lead to slower decapping rates (of deadenylated transcripts), independent of a direct effect by NMD.
7. Literature supports a model that Upf1 samples all transcripts and binds mRNA independent of translation. Under this model, Upf1 would be expected to associate disproportionally with stable, highly abundant transcripts that lack PTCs, and transcripts with longer 3’ UTRs – which is consistent with the presented data. Curiously, PTC-containing transcripts with long poly(A) tails do not copurify with Upf1 (Fig. 4B) – despite being the canonical class of NMD substrates. A likely explanation for this is that these transcripts are rapidly degraded and devoid in the sample; however, if Upf1 also targets short poly(A) transcripts to rapid decay by NMD, why are they present in the sample and copurified with Upf1?
8. There are several concerns around the RNA-Seq analysis:
a. The ‘moderate’ correlation of Upf1-associated transcripts identified by Illumina versus ONT sequencing is concerning (Fig. S3; R=0.48). Despite inherent differences in this approach, I would not expect this low of agreement on the transcripts that copurify with Upf1.
b. Ribosomal proteins represent a large portion of the genes in the noPTC category of transcripts crosslinked to Upf1 (CRAC data Fig.2) and many are known to contain introns. How are the authors able to distinguish Upf1 binding to unspliced mRNA, which would be expected to be targeted to NMD. Was Upf1 found to CRAC to intron-containing sequences for these transcripts?
c. Inclusion of partially degraded transcripts in the ONT data is misleading.

(Remarks on code availability)

Version 2:

Reviewer comments:

Reviewer #2

(Remarks to the Author)
With this second revision, the authors have addressed my remaining concerns. By replacing the highly speculative model figure with a data-based summary illustration, the manuscript is now scientifically more robust and focused on presenting the many interesting results rather than emphasizing the speculative interpretation.
I have no additional comments and judge this manuscript ready for publication.

(Remarks on code availability)

Reviewer #3

(Remarks to the Author)
The main issue with this work is not whether UPF1 associates with mRNA in yeast lacking a PTC or having a short polyA tail – this data is sufficiently convincing (although the authors have still not addressed the specificity of UPF1 for only a subset of mRNA within this category). What remains unclear is whether UPF1 association is meaningful to the turnover of bound transcripts and whether these mRNAs are, in fact, targeted to NMD – this remains unclear and not fully supported by the data. It is now admitted by the authors that the data does not distinguish between a direct or indirect role for NMD in the turnover of short polyA-tailed mRNA, and so it can not be concluded that NMD TARGETS these transcripts.

It is noted that data supporting a mechanism for UPF1 recruitment to short-tailed mRNAs is scant (and does not account for why only some of these mRNAs are targets). Indeed, the authors have removed their mechanistic model due to lack of direct evidence or contradictory findings (Fig. 8) but still invoke a role for PAB in the Discussion. The replacement for Fig. 8 represents a Graphical Abstract (of 'expected results') and is not helpful to the reader.

(Remarks on code availability)

Open Access This Peer Review File is licensed under a Creative Commons Attribution 4.0 International License, which permits use, sharing, adaptation, distribution and reproduction in any medium or format, as long as you give appropriate credit to the original author(s) and the source, provide a link to the Creative Commons license, and indicate if changes were made.
In cases where reviewers are anonymous, credit should be given to 'Anonymous Referee' and the source.
The images or other third party material in this Peer Review File are included in the article's Creative Commons license, unless indicated otherwise in a credit line to the material. If material is not included in the article's Creative Commons license and your intended use is not permitted by statutory regulation or exceeds the permitted use, you will need to obtain permission directly from the copyright holder.

To view a copy of this license, visit https://creativecommons.org/licenses/by/4.0/

Point by point answer to referees

We thank the reviewers for their comments and questions, as they showed where we failed to clearly present and explain our results and oriented further validation experiments that are now shown in the revised version of the manuscript. We tried to simplify the presentation of the results, improve the explanations about how heterogenous populations of mRNA can be analysed with different sequencing methods and performed further data analyses that should answer all the major issues raised by the reviewers.

REVIEWER COMMENTS

Reviewer #1 (Remarks to the Author):

Manuscript by Gouhier et al., examines the collection of Upf1 targets on a transcriptome-wide scale. Authors purify RNAs associating with Upf1 and employ both Illumina and nanopore sequencing to evaluate mRNA isoform enrichment. The techniques employed are adequate to the question, and thus the paper would add a quite substantial RNA-seq RIP dataset, along with steady state datasets of Upf1/2/3 single delta strains. As expected transcripts containing premature codon stop (PTC) emerge as targets (mostly non-coding SUTs/XUTs and specific mRNAs). Such types of targets were previously numerously examined using reporter transcripts. What is surprising is that the authors propose that a subfraction of mRNAs containing short pA-tails is also subjected to Upf1-mediated nonsense mediated decay. This is based on Illumina-RIPseq and on a general accumulation of short tailed mRNAs in upf1-del, upf2del, upf3del. This is the novelty, which is notable to a broader audience of Nature Communications. Overall I think that the data itself looks sound, but the depth/presentation of the data is somehow convoluted, at least to me, and perhaps relying too heavily on the Illumina-RIPseq.

Major:

1. the Upf1-RIP-Illumina dataset is used to define enrichment of Upf1 targets. Next the Upf1-RIP-Nanopore dataset is used to establish pA-tail profiles. However, fold-change enrichment of RNAs in both datasets appear quite different (Fig. 2D), despite the statement in the text. This is only 'my biased reviewer eyes', and perhaps I am mistaken. Therefore I think that the authors should provide a Pearson/Spearman correlation coefficient to support their statement. If it turns out low they should justify why. That said, the nanpore datasets tend to have lower read yeild (at least in the old chemistry, is it the case here?) so I don't really think they should be used to determine enrichment at all. Hence, I recommend to ammend this figure and move it to the supplement.

The Pearson correlation value was present in the original text in the legend to Fig 2d, but was not directly commented in the text. We moved Fig. 2d to Supplementary Fig. 3a and modified the corresponding paragraph accordingly (page 5 lines 35-45):

"Despite biases inherent of both techniques, the global trend showed a moderate although positive correlation (Pearson correlation coefficient r=0.48072). To further understand these results, we compared transcript ranks obtained from Nanopore and Illumina sequencing (rank 1 being the most abundant and 12296 the least abundant – Supplementary Fig. 3b). We noticed

a strong positive correlation in the three scatterplots (with a Spearman correlation coefficient >0.8), indicating that despite variations inherent to Illumina and Nanopore technics, the relative estimated abundance of transcripts is comparable and consistent between the two methods (Supplementary Fig. 3b). However, the difference of rank showed a wider range in Upf1-RIP than in upf1Δ experiments (plot Rank WT – Rank Upf1 RIP compared to Rank WT – Rank upf1Δ Supplementary Fig. 3c and 3d), that look similar to foldchange comparison shown in Supplementary Fig. 3a.".

Nanopore datasets provided effectively lower yield with the R9.4 flowcell version, but were performed in triplicate and without multiplexing and with a depth good enough that the obtained values were usable with Deseq2 (see SupplementaryData3_GSE284490_Nanopore.xlsx), so we are confident that Nanopore results are also reliable. To complete the analysis, we made a comparison of transcript ranks obtained from Nanopore and Illumina sequencing. It yielded a strong positive correlation in the three comparisons for different samples (Spearman correlation coefficient >0.8, see new Supplementary Fig. 3b). This support that both Illumina and Nanopore Upf1-RIP experiments are comparable and that the differences observed can be attributed to technical variations. It is expected that different methods yield in different results: Illumina sequencing contains a fragmentation step and a PCR and was performed on ribodepleted mRNAs, whereas Nanopore sequencing has a priming step with oligodT. In addition, both sequencing series resulted from a different set of RNA Immunoprecipitation experiments, with unavoidable variations in the purification efficiency.
In order to highlight the relative enrichment by rank, we also calculated the difference of ranks in WT – ranks in *upf1Δ* or Upf1-RIP (new Supplementary Fig. 3c-d). In this context, transcripts with similar ranks in both conditions tend to zero, and transcripts enriched or depleted are displaced in the scale.
Results obtained with *upf1Δ* show this grouping around zero for noPTC categories (meaning that the overall ranking is not affected by the absence of Upf1), whereas PTC-NMD targets tend to be present towards positive values because they are stabilized in the deleted strain (Supplementary Fig. 3c). However, despite a similar rank correlation in RIP experiments, delta ranking has shown a more variable profile in Upf1-RIP experiments (Supplementary Fig. 3d).
Finally, Rank WT – Rank Upf1 RIP scatterplot (Supplementary Fig. 3d) resembles to FoldChange scatterplot (old Fig 2D, new Supplementary Fig. 3a), and confirms the enrichment differences between Illumina and Nanopore, consistent with the fact that the observed differences are inherent to the methods used.

2. Authors interpret the pA-tail profiles of steady-state total upf1/2/3 delta strains through the results of the Upf1-RIP. I do think that in the absence of spike-ins/proper normalization of the RIP (by background eg. ncRNA sno/sn?; might be difficult) its value should be treated as limited. Justification:

Model states that Upf1 is recruited to short-tailed mRNAs because there is no Pab1 on the tail. upf1/2/3 deltas do accumulate short-tailed mRNAs globally (Fig. 6), so this fits the model. But the UPF1-RIP noPTC_enr and noPTC (not enriched) mRNAs both accumulate short-tailed mRNAs to the same extent (Fig. 3C). So it seems that the Upf1-bound and -unbound mRNAs react the same way, which argues with the model. Are you really sure that the Upf1-RIP-Illumina data reliably determines the set of targets? Perhaps a CRAC experiment to complement this would be better? Nobody will expect such a heavy experiment in revision. I would just ask to reconsider the structure of the publication.

Indeed, noPTC_enr and noPTC transcripts can both bind to Upf1 even if their level of

enrichment in Upf1-RIP is different (old Fig. 3c = new Fig. 4c). NoPTC_enr have a fold change > 2 in Illumina results for RIP, whereas noPTC (not enriched) have a fold change < 2. Enrichment computation depends on the relative amounts of Upf1-associated transcript compared with the transcript in the input sample. If we were unable to distinguish different population of transcripts by the size of their poly(A) tails, we would agree with the reviewer. However, we have demonstrated that the noPTC transcripts associated with Upf1 represent only a specific fraction of the noPTC mRNA, specifically the one with shortened poly(A) tails (see Fig. 4c and *TDH3* example in Supplementary Fig 4a). Thus, "not enriched", does not necessarily mean "not bound". mRNAs co-purified with Upf1 in the RIP experiment, no matter they can be classified as enriched or not, contain mRNA with shortened poly(A) tails compared to the input (see Supplementary Fig. 3a, *HHT2* and *TDH3,* and Fig. 4a).
If we see the transcripts for a given gene as a mix of various poly(A) tail mRNA, with only the short poly(A) ones being bound by Upf1, apparent enrichment in a RIP will depend on how large is the fraction of short poly(A) mRNA and their dynamic. For transcripts, such as HHT2, present mostly as a short poly(A) tail mRNA in the total extract, we can measure enrichment with Upf1, as most of the HHT2 mRNA can bind Upf1. For other transcripts, even if Upf1 can bind to the oligo(A) mRNA fraction, if this fraction is low, no enrichment can be measured. Even if not enriched by Upf1, the mRNA detected in the immunoprecipitation is different than in the input RNA, and is specifically recognized by Upf1. It is thus improper to qualify it of « unbound », but rather « not enriched » , which solves the major concerns of reviewer #1 and #3.

Regarding the normalisation, we performed DESeq2 normalization with the option « shorth ». The "shorth" option (for shortest half) is a robust estimator of the central tendency of a distribution. Compared to the mean or median, the shorth is less sensitive to extreme values, making it particularly useful for distributions that are skewed or contain outliers as it is often the case in RIP-seq.

Finally, to confirm the specific affinity of Upf1 for noPTC transcripts, we have performed CRAC experiments and confirmed a specific binding of Upf1 on noPTC and noPTC_enr transcripts. Notably, Upf1-CRAC signal is weaker and more spread on noPTC transcripts and stronger and more localized in the 3'UTR of noPTC_enr transcripts. We also observed a signal in PTC transcripts (see examples in new Fig. 2 and Dataset GSE299782). We added new Figures in the manuscript to present these results (Fig. 2).

3. paper-structure-wise: Fig. 3 should go before Fig. 2 (global analysis and then single mRNA examples). Fig. 6 is the most valuable in my view (especially panel 6B). Perhaps targets should be first defined by selecting mRNAs that have the most altered pA-tail profiles, which would then be compared with the RIP-seq datasets (both Illumina and nanopore).

We changed the structure of the manuscript by adding a figure (Fig. 2, CRAC experiment) that identify direct RNA targets of Upf1 and by removing Figure 4, considered not very informative by the reviewers. However, we did not switch the order of the old Fig. 2 and Fig. 3 (now Fig. 3 and Fig. 4) since we think it is pertinent to exemplify the power of Nanopore sequencing before presenting genome wide data.

We defined noPTC and noPTC_enr categories from Illumina sequencing data, since it was until now the gold standard for RNA quantification. CRAC experiment further refined our understanding of the different behaviour of Upf1 regarding theses two categories.
Nanopore sequencing revealed a comparable behaviour of Upf1 on noPTC and noPTC_enr transcripts regarding the poly(A) tail status, finding that binding to the short poly(A) fraction of transcripts is a common feature for theses two categories. We concluded that differences in enrichment status are the result of differences between the relative amounts of short poly(A) tail mRNA for different genes. Finally, the subfraction specifically detected by Upf1 (corresponding to short poly(A) tailed mRNAs) is stabilized when NMD is inactivated.

4. a more in-depth global analysis of extra features of Upf1 mRNA targets would be appreciated. I do like the single mRNA examples in Fig. 2 but I wonder how general they are?

Examples from Figure 2 (now Figure 3) are shown to highlight the power of Nanopore sequencing compared to Illumina. In our analysis, we distinguished transcriptome-wide the pre-mRNAs from the mature mRNAs by computationally filtering reads covering the intron part of mRNA. This is made by the Filtering step (code on Zenodo), that distinguishes and separates intron (pre-mRNA) from intron-less isoforms (mRNA). These results are listed in SupplementaryData3_GSE284490_Nanopore.xlsx

In order to present a more in-depth global analysis, we added MA plot comparisons such as in Fig. 1b (new Supplementary Fig. 5a), where transcripts from categories noPTC, noPTC enr, XUT_SUT and PTC are shown. It shows that the absence of Upf2 or Upf3 causes the loss of Upf1 affinity for all the mRNA.

Finally, since CRAC experiment confirmed Upf1 mRNA binding and position on mRNA, we analysed globally features related to noPTC and noPTC_enr transcripts, and found that the ORF and UTR length could be determinant to distinguish these categories, noPTC_enr harboring slightly shorter coding sequences and longer 3'UTR than noPTC (see also new supplementary Fig. 1d-e).

5. What is the correlation between pA-tail length and RNA abundance change in upf1, 2 and 3 strains?
To answer this question, we examined the relationship between poly(A) tail length and mRNA levels in different conditions. The next figure contains scatterplots representing the poly(A) tail length as a function of Abundance. It demonstrates that poly(A) length observed in Upf1 RIP upon *upf2* or *upf3* depletion are nearly identical to Total input in theses background and differ from the Upf1 RIP in a WT context.

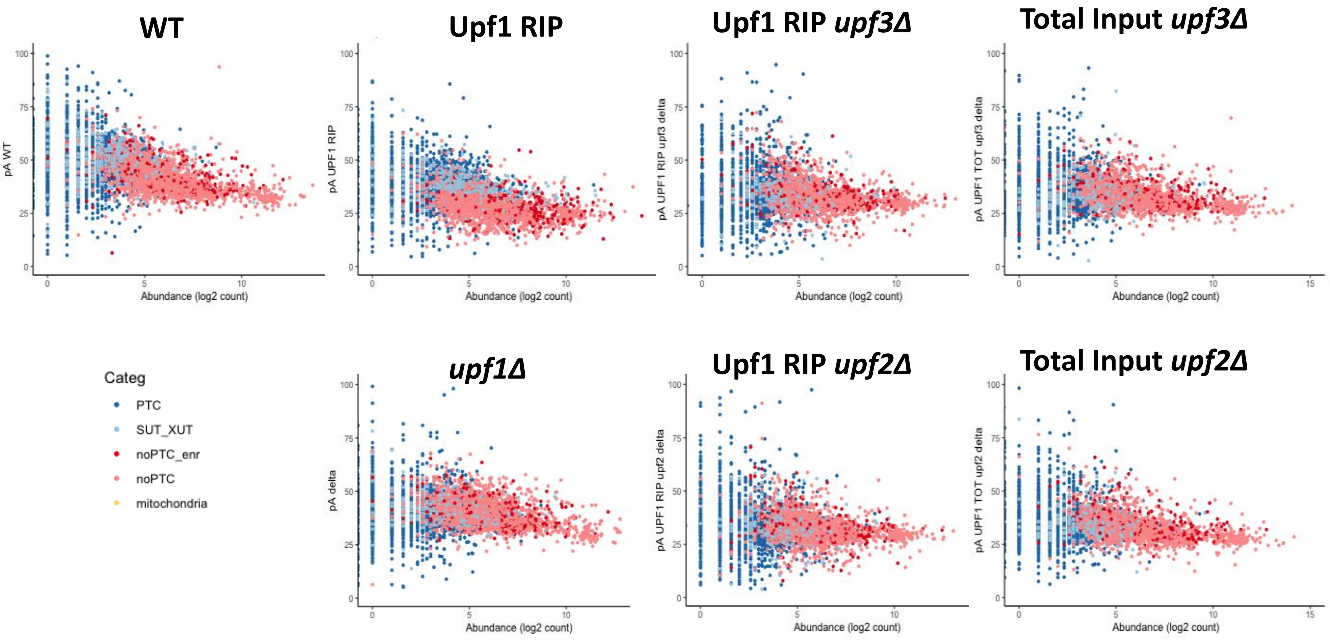

6. There is no login for reviewers to check the RNA-seq raw data submissions. Only accession numbers(GSE284490, GSE283053) are given. GEO informs that such datasets exist but are currently private. Therefore the Reviewer cannot confirm deposition of the raw data (in accordance with journal requirements).

Tokens for data access for reviewers were provided in the initial submission but were probably not obvious to find, we are sorry for that. Since the manuscript is now available as a preprint, we made the data publicly available.

Minor:

1. somehow define the identity of upf1 targets (selected from RIP or total). Are they abundant mRNAs? Long-short-tailed by nature? any functional groups?

The Upf1 targets are listed in Table S1 and Supplementary Data2_GSE283053_Illumina.xlsx and Supplementary Data3_GSE284490_Nanopore.xlsx. Aside the PTC containing transcripts, which have longer than average poly(A) tails, as expected from their short life that does not allow deadenylation to occur, the noPTC mRNAs correspond to a wide variety of transcripts that show similar codon usage and display some differences in coding sequence length and 3’ UTR length (now shown in Supplementary Fig. 1c, d, e and Supplementary Fig. 2). We added MA plots (FC in Upf1 RIP or in *upf1Δ*, *upf2Δ* or *upf3Δ* compared to WT or Input) to report the relative abundance of each transcript per category (see Supplementary Fig. 5).
GO term enrichment search did not identify any relevant functional group (half of noPTC_enr transcripts belong to the very large “protein binding” subcategory, so does the noPTC subgroup).

2. Fig. 1C -> please add one column marking what percentage each group represents as transcriptomic ORF/ncRNA total. Specify which groups are coding.

We updated Fig. 1c accordingly.

3. Fig. 2C -> pA-tail profiles for single mRNA examples. Please transfer pA-tail profiles from supplement S2A to main 2C. I think comparison of WT, upf1del totals to Upf1-RIP is the only correct way to show the data. It is important that in upf1del the overall profile shifts towards shorter pA-tails, and that the Upf1-RIP fraction displays even shorter pA-tails. I am missing a more global analysis for non-PTC_enriched groups that would support this for a larger number of single mRNAs. Single gene examples are a support but no definitive proof.

We updated the old Fig. 2c (now Fig. 3c) with the poly(A) profiles in *upf1Δ*, WT, Upf1 RIP and eIF4G RIP. In addition, initial Supplementary Fig. 2b (now Supplementary Fig. 4b) shows the global behaviour of poly(A) per categories of transcripts. PTC transcripts in the WT have much longer poly(A) tails globally compared to *upf1Δ* where they are stabilized. Similarly, other categories, including noPTC, noPTC_enr and even PTC_pos and unknown present shortened poly(A) tails in Upf1-RIP. The number of transcripts in each category is the same as in Fig. 1d and covers a large number of individual genes.

4. Fig. 5 -> the pA+-selection experiment. Here, different mRNAs are examined compared to Fig. 2. I wonder whether in the various pA- and pA+ fraction authors could differentiate for various isoforms given as examples in Fig. 2? Perhaps by adding a RNaseA digestion coupled to trimming the 3'UTR at the beginning to facilitate length differentiation using a 3'UTR probe?

We have voluntarily chosen for Fig. 5c representative example of the three relevant categories. Examples shown in Fig 2 illustrate the power of Nanopore sequencing, but they have features that make them difficult to analyse by other methods (in particular *RPL8A* with its two isoforms). We appreciate the suggestion of using enzymatic treatments to help differentiate different poly(A) length isoforms, but such treatments might reveal to be problematic and highly dependent on the abundance of the transcripts studied.

5. Please improve the description of Fig. 1B (MA plot, please write on x-axis the same description as in the legend).

We modified the legend accordingly.

6. Fig. 2B -> comparison of RIP-Illumina and RIP-nanopore reads. Please draw a scheme of the genomic locus with ORF and pre-mRNA cleavage sites marked. Can you clearly distinguish two cleavage sites based on the sequencing data?

We modified the Figure (now Fig. 3b) accordingly, to clearly indicate isoforms.

Yes, we can distinguish cleavage sites, especially in the WT, since long reads come in one sequence per mRNA, so reads that contain introns are easy to separate from reads that do not. Bigwig files do not provide the details of reads such as in BAM, but the shape of the signal gives information about the structure of the read. In the *RPL22B* example, reads from the upper side correspond to mature mRNA because they have sharp edges that correspond to the exon-exon junction (visible on the IGV screenshot from BAM files below). It is certain for the reads that overlap exon 1, and considering the sharp edge of the others and the fact that they do not contain the intron, it is most likely that they are also mRNA, even if we cannot exclude at 100% that some of them could also be degradation fragments.

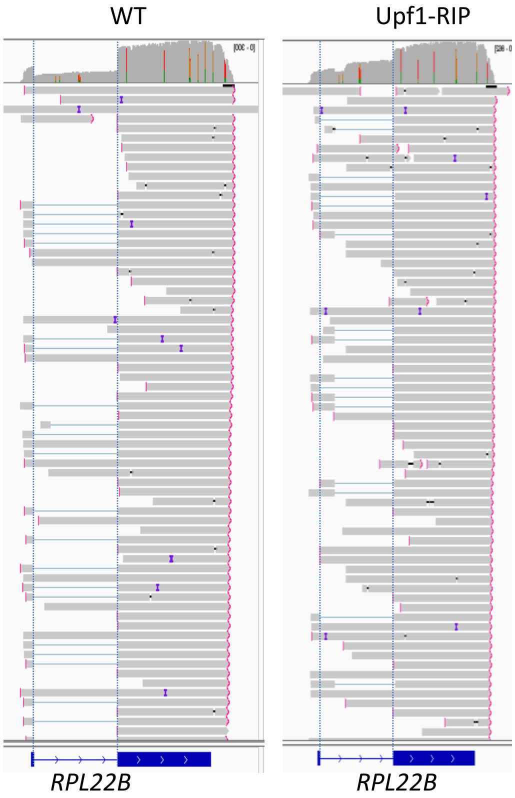

7. Fig. 2D -> Please introduce a diagonal. It will make the graph easier to read.
We modified the Figure 2d (now Supplementary Fig. 3a) accordingly.

8. The paragraph concerning Fig. 3A is written in a rather convoluted manner, it is not clear when the authors refer to input (total) sample and when to RIP samples. Please improve.
We modified the text to avoid confusion - now “WT total input” is used instead of “WT input”.
9. Page 6 line 5 -> useless '_' ('Fig.2A)._These')
We modified the text accordingly.

Reviewer #1 (Remarks on code availability):

code submission embargoed until end dec. 2025
Tokens for reviewers were provided at the initial submission. Since the manuscript is now available as a preprint, we made all the code publicly available without the need of a token.

Reviewer #2 (Remarks to the Author):

Gouhier and colleagues applied here RIP-seq (Illumina-based) and Oxford nanopore direct RNA sequencing (ONT-DRS) to identify Upf1p-bound transcripts and assess their poly(A) tail length. The RNAs identified in the RIP-seq were separated into different categories. Classical NMD targets like SUTs/CUTs and PTC-containing mRNAs were enriched as expected. Interestingly, approx. 15% of mRNAs without an apparent PTC were > 2-fold enriched in the Upf1p-IP (designated noPTC enr) but not upregulated in upf1Δ cells. The authors then focused on this population of stable RNAs that are associated with Upf1p yet not degraded by NMD.

1) Next, they performed ONT-DRS on the input, Upf1p-bound, and eIF4G-bound RNA samples in WT and upf1Δ cells. Interestingly, transcripts (**both PTC and noPTC enr**) with short poly(A) tails (0-25 As) were strongly enriched among the Upf1p-bound transcripts, while the eIF4G-bound sample enriched transcripts with medium (>25 – 50 As) and long (>51 As) poly(A) tails, suggesting that many of the Upf1p-bound transcripts might represent decapped decay intermediates. Given that NMD was reported to occur independently of deadenylation, the enrichment of RNAs with short poly(A) tails is unexpected and seemingly contradicts a previous publication of the authors, in which they reported that PTC-containing transcripts on average contained longer poly(A) tails than other transcripts (Audebert et al., 2024).

Our results on transcripts that are not known to be NMD substrates, but also on PTC containing NMD targets are compatible with previous publications. PTC transcripts (NMD targets) with short poly(A) tails **were not particularly** enriched among the Upf1-bound transcripts (see Fig. 4c) and this is consistent with the deadenylation-independent degradation of NMD targets (in which "NMD targets" correspond to our "PTC containing-NMD targets"), and to the results from Audebert *et al*, EMBO J 2024. These PTC targets (and XUT/SUT targets) are enriched with medium tailed mRNAs whereas all the other categories are enriched with short tailed mRNA (noPTC, noPTC_enriched, PTC possible and unknown, see also Supplementary Fig. 4c). In conclusion, the specific binding of Upf1 to short poly(A) tail transcripts is restricted to "non-PTC-NMD" mRNA *sensu lato*.

2) The authors then went on to present data from a polysome gradient experiment (Fig. 4) of which I can neither judge the data quality nor understand what the authors' conclusion is from this data. Moreover, the use of a histone mRNA (HHT2), which in mammalian cells is known to bind UPF1 for its induced rapid decay at the S phase (cell cycle-dependent histone mRNA decay), is questionable. The authors should therefore test at least one, better two other, more representative mRNAs of the noPTC enr category. Since the data in Fig. 4 does not strengthen the overall conclusions of the manuscript, and at the same time raises criticism regarding the quality of the experiment, I suggest to remove it from the manuscript.

We agree that the polysome gradient experiment was not essential to this study and is dispensable. It showed that the short-tailed HHT2 mRNAs were present in heavy polysomes and they were displaced to lighter polysome fractions when Upf1 was absent. We removed it from the revised version of the manuscript.

As requested, we quantified the amount of two other non-histone transcripts of the noPTC_enr category, and of *RPL6B* and *HCR1*, in the short, medium and long poly(A) subfractions. We observed the same trend as reported in the original version of the manuscript for *HHT2* for these transcripts: an increase in mRNA levels in the short fraction upon UPF1 deletion (new Fig. 5c and Supplementary Fig. 6b). Thus, the effect of Upf1 on short poly(A)-tailed mRNA is not restricted to the histone transcripts.

3) Using a biochemical approach to experimentally separate RNPs into three fractions with (i) no or short (S), (ii) medium (M), and (iii) long (L) poly(A) tails, the authors next assessed the distribution of HHT2 (noPTC enr transcript) and pre-RPL28 (PTC transcript) RNAs from WT and upf1Δ yeast in this poly(A) length fractions. Most of the NMD-targeted transcript (pre-RPL28) is present in the L fraction in WT cells and shifts towards M and S fractions in upf1Δ cells, confirming previous observations that NMD usually occurs on transcripts with long poly(A)

tails. Compared to WT cells, HHT2 mRNA was enriched in the S fraction in the upf1Δ cells, consistent with the hypothesis that as in mammals, Upf1 might be required for the cell cycle-dependent histone mRNA decay also in yeast. Cell-cycle-dependent histone mRNA decay is not well understood in yeast and whether Upf1 plays a role is unclear. Therefore, the data here is potentially highly interesting, but the authors do not discuss this aspect.
Since TDH3, a noPTC mRNA behaved similarly to HHT2, one could also argue that the previously described role for mammalian UPF1 in histone mRNA decay is only the tip of the iceberg and that Upf1 is generally involved in degrading deadenylated mRNAs with only a few As left, which is the interpretation of the authors.

We thank reviewer #2 for raising this point. We included additional considerations about histone transcripts in the discussion.
To evaluate the effect of Upf1 on mRNA with shortened-poly(A) tails in yeast grown asynchronously in bulk, we needed to separate the RNA in S (Small poly(A) tails), M (medium length) and L (for long poly(A) tail length) fractions and we observed the accumulation of *HHT2, HCR1, RPL6B* or *TDH3* transcripts only in the sub-fraction corresponding to short poly(A) tails, but not at the level of the total RNA, consistent with a short-tailed subpopulation of these transcripts being targeted by Upf1 and the NMD machinery. Additional experiments (e.g. analysing histone mRNA dynamics across the cell cycle in *upf1Δ* cells in synchronized yeast) would be needed to investigate whether this represents an important conserved cell-cycle–dependent mechanism. Yet, we believe that such involved experiments are beyond the scope of the current manuscript. Herrero and Moreno (EMBO J 2011) have shown a strong post transcriptional regulation of Histones transcripts, dependent of Lsm1. This is interesting and it could be related to the short poly(A) NMD pathway, since half of the histone transcript fall in the noPTC_enr category (HTA2, HTB2, HHF2, HHT2, and HTZ1). The paper and this work are now mentioned in the discussion section.

Histone level variations during the cell cycle are radical and are believed to be mainly driven at the transcriptional level (Eriksonn et al, Genetics 2012) which can consistently not be fully explained by the subtle post transcriptional regulation that we observed in asynchronous yeast. However, the possibility that Upf1 could also contribute to histone mRNA turnover in yeast cannot be excluded. We agree that this point is highly interesting and deserves further investigation.

The parallell between Upf1-mediated regulation of Histone transcript in mammals and a potential role of Upf1 in such regulation in yeast is also interesting. Mammalian Histone transcripts are deadenylated and their degradation via Upf1 is mediated by SLBP which is not conserved in yeast (Kaygun and Marzluff, NSMB 2005). In addition, such degradation in mammals also depends on a poly-uridylation step operated by TUTase (Meaux et al 2018), which are also absent in yeast. It is possible that the evolution has selected Upf1 to post-transcriptionnally regulate Histone transcripts in yeast by a different route that those in mammals.

We added a paragraph discussing these points (page 12 line 1_19).

The short poly(A)-NMD could be an important cellular process to post transcriptionally regulate histone transcripts levels. Cell-cycle-dependent histone mRNA decay is not well understood in yeast and whether Upf1 plays a role is unknown. Mammalian histone transcripts are deadenylated[53], and display a specific post transcriptional regulation by Upf1 independently of Upf2 and Upf3, and mediated by an interaction with the cofactor SLPB that

recruits Upf1 on a stem loop on these mRNAs [54,55]. Contrarily to human, yeast histone transcripts are polyadenylated and SLBP is not present, thus whether Upf1 plays a role in their regulation is unknown. Histone post transcriptional regulation seems also driven by LSM1 in yeast as histone transcripts strongly accumulate in a lsm1Δ mutant following hydroxyurea treatment[56].

In our study, we found that Upf1 has an intrinsic affinity for the noPTC_enr transcripts (Fig1 and 2) that are more susceptible to be affected by the short poly(A) NMD route. These mRNAs include half of the histone transcripts (HTA2, HTB2, HHF2, HHT2, and HTZ1). The others, (HTA1, HTB1, HHF1, and HHT1) belong to noPTC and HHO1 to PTC_pos categories and do not seem to have a such a strong affinity for Upf1. Additionally, we demonstrated a role of Upf1 in the decapping of HHT2 (Fig 6). Altogether, our observations suggest that both Lsm1 and Upf1 could coexist on the same mRNP to trigger the decapping and targeted degradation of histone transcripts in yeast by short poly(A) NMD. It is possible that the evolution has conducted Upf1 to post-transcriptionally regulate Histone transcripts in yeast by a different route that those in mammals.

4) To further investigate the effect of Upf1p on the stability of normal, not NMD-targeted mRNA, the author assessed the half-life of a poly(A) length-fractionated tet-off regulated HIS3 reporter RNA. They found that in upf1Δ cells, the half-life of the HIS3 RNA with a short or no poly(A) tail (fraction S) was strongly increased, while little and no stabilisation was observed in the M and L fractions, respectively. This data elegantly and compellingly confirms that Upf1 is required in selectively targeting transcripts with short or no poly(A) tails for degradation. It would be really nice if for comparison and completeness, the same experiment were also done with an NMD-targeted reporter RNA.

Considering the very short half-life of a PTC-NMD target, it is challenging to measure the signal in a kinetic experiment in WT conditions. While we agree with the reviewer that the proposed experiment would be nice, it is technically highly challenging due to the low levels of the NMD reporter and its short life. However, we do not need a kinetic to observe the important stabilization of PTC-NMD targets upon Upf1 deletion because this population is homogeneously targeted to NMD (see Fig. 5b Tot *pre-RPL28*)

5) Finally, to address if other NMD factors are also required for the degradation of short poly(A)-tailed noPTC mRNAs, the authors measured the distribution of poly(A) tail length by ONT-DRS in input and Upf1-RIP samples from WT, upf2Δ, and upf3Δ strains. Upf2p and Upf3p depletion increased the fraction of short poly(A)-tailed RNAs in the input samples even more than upf1Δ (compare Fig. 3A with 6B), but in the Upf1-RIP samples, more RNA with short poly(A) tails is pulled down in WT cells than in upf2Δ, and upf3Δ cells. While the latter observation remains uncommented by the authors (see my comment below), the input data suggests that not only Upf1p but also Upf2p and Upf3p (i.e. NMD) are involved in the degradation of noPTC mRNAs with short poly(A) tails. The finding that in the absence of Upf2p or Upf3p, Upf1p-assocaited mRNAs have longer (medium) poly(A) tails does not fit in the proposed model (Fig. 7), should be discussed, and the model adjusted.

We have shown that in the absence of upf2 or upf3, Upf1 losses its affinity for short tailed RNA (Fig. 6b). The fact that Upf1-associated mRNAs have more (medium) poly(A) tails in the absence of Upf2 and Upf3 is a consequence of the relative loss of short tailed mRNA, since results are expressed in percentages (small + medium+ long = 100%). Total RNA Input distribution of transcripts among poly(A) categories and Upf1 RIP were very similar in the absence of Upf2 or Upf3 (Supplementary Fig. 5c, Supplementary Fig. 7c and Fig. 6c). We added

MAplots to illustrate the loss of affinity of Upf1 to mRNA upon *upf2Δ* or *upf3Δ* (Supplementary Fig. 5) and we modified Fig. 6c to emphasize the population of short poly(A) tailed mRNAs.

Overall, this study of the poly(A) tail lengths of Upf1p-associated mRNAs with ONT-DRS is original and has provided interesting and surprising findings. While the polysome fractionation experiment cannot be assessed, the rest of the data appear to be of high quality and convincing. Apart from some points made above and below, the authors carefully and thoughtfully interpreted the data and provide evidence for a novel function of Upf1p in the degradation of deadenylated RNAs with short or no poly(A) tails remaining. Whether Upf2p and Upf3p, and hence NMD, are also involved is not fully supported by the presented data. In my opinion, the role of Upf1p in the decapping of these short poly(A)-tailed RNAs would be worthwhile exploring, as this is likely the degradation route of these RNA species. Without clarification of this point, the presented model (Fig. 7), although appealing, remains highly speculative.
To address the role of decapping in the Upf1-mediated stabilisation of short poly(A) transcripts, we developed a test that quantifies the relative proportion of 5' cap and 5'P end, and validated the global role of Upf1 in the decapping of noPTC_enr transcripts (*HHT2* and *RPL6B*) and to a lesser extent on a noPTC mRNA (*TDH3*). The obtained results were validated by using a strain in which the decapping enzyme Dcp2 was depleted. We added these results in a new Figure (Figure 7). These new results answer the question of the reviewer, in particular the fact that decapping is responsible for the degradation of short poly(A) fraction of transcripts.

Moreover, as discussed by the authors, the model predicts that Upf1p-bound short poly(A)-tailed mRNAs should lack Pab1 association and vice versa, a prediction that could and should be tested.

To answer this question we tried to tether Pab1 to a reporter transcript to see if this would change the association of the transcript with Upf1. However, these tethering experiments are technically challenging and were not successful. Despite the fact that we could not easily adapt a tethering assay to the short-tailed subpopulation, we already know that, for example, the population of *HHT2* mRNAs associated to Upf1 is short tailed, and different from that associated to Pab1 (Figure EV2 D from Audebert et al, 2024 extracted below).

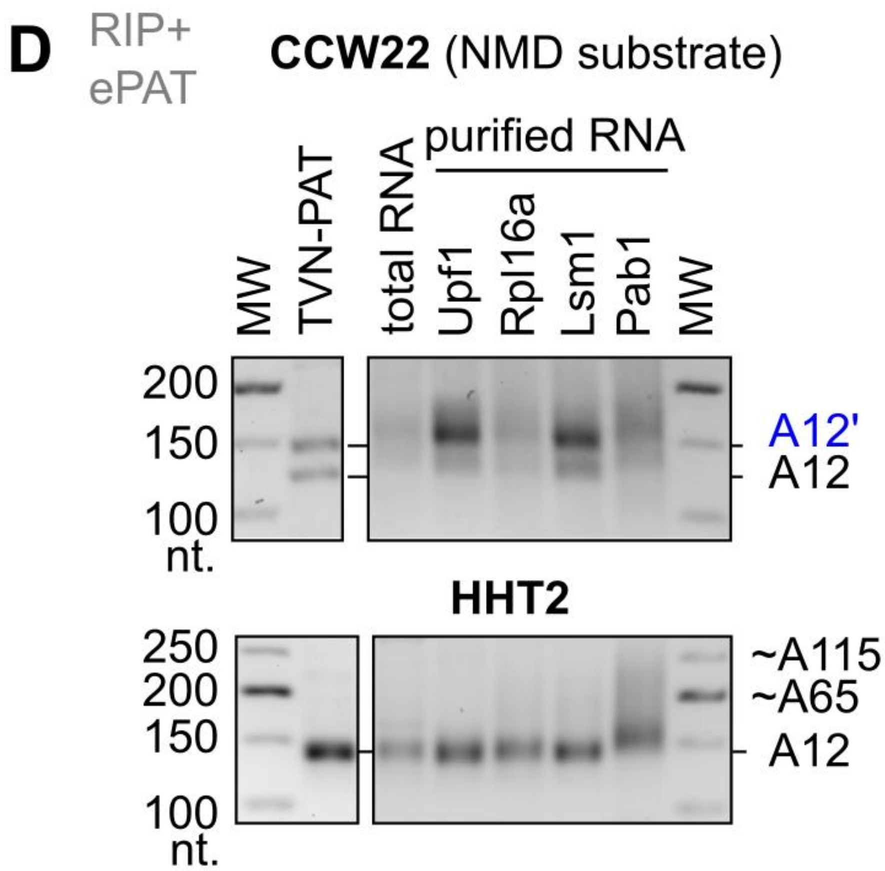

In the *HHT2* example (figure EV2 from Audebert et al, EMBO J 2024), poly(A) tail length shows the majority of tails being relatively short in the Total RNA sample. Interestingly, Upf1-associated *HHT2* transcripts were very similar. Strikingly, it is a different subpopulation of *HHT2* transcripts that is associated to Pab1, harbouring much longer poly(A) tails (lane on the right). This strongly support our model as it suggests that *HHT2* mRNA is globally poorly associated to Pab1 (lane Total RNA), contrarily to the PTC representative CCW22, and that the population detected by Upf1 is different than that bound by Pab1 and considering their very short poly(A) length, might reasonably lack Pab1.
We added a paragraph in the discussion to point this previous work partially validating our model (page 11, line 25-33) :

We have previously shown that the population of HHT2 mRNAs in the WT Total RNA sample harbour short poly(A) tails and is most likely poorly associated to Pab1, as the Pab1-associated HHT2 poly(A) tails are much longer (see Figure EV2 from Audebert et al [27]). Strikingly, Upf1-associated HHT2 harbours shorter tails than the total RNA. These results suggest that Pab1 is absent on the HHT2 mRNAs associated to Upf1 and Lsm1. This is consistent with previous work demonstrating that NMD can be active even in cells lacking Pab1 and for reporters devoid of a poly(A) tail [49]. Although we couldn't measure the effect of tethering Pab1 nor eRF3 on noPTC_enr mRNAs (not shown), we could evaluate the significant effect of Upf1 on the decapping of noPTC and noPTC_enr mRNAs (Fig. 7).

.

Additional points:

Double-check references: some references in the introduction seem not to fit. For example, ref 9 does not describe the faux 3´UTR model, instead it refers to the very first description of the NMD phenomenon. Also, the sentence on p.2, lines 5-7 needs one or more refs.

We modified the text accordingly.

p.2, lines 24-27: For clarity, it should be mentioned that the cited studies pertain to mammalian cells, where UPF1 phosphorylation rather than its association with RNA appears to be the distinctive marker for NMD, whereas in yeast, extensive Upf1p phosphorylation seems not to occur and it is therefore proposed that Upf1p binding to RNA identifies NMD-targeted transcripts.

We modified the text accordingly

p.2, lines 35-37: Please explain how you arrived from the numbers in Fig. 1C. to the slightly smaller numbers indicated in Fig. 1D.

Figure 1c numbers represent all the possible transcripts that we categorized in Supplementary Data 1. Figure 1d numbers represent all the transcripts from this previous list, that were detected in our RIP-seq experiment (in SupplementaryData2_GSE283053_Illumina.xlsx).

Fig. 2D: While fewer Upf1p-associated transcripts were identified with ONT-DRS than with Illumina, which is consistent with the expected lower sequencing depth, you curiously detected now 107 Upf1p-enriched noPTC transcripts. What is the explanation for this? Were they all close to the threshold and just represent the variability in the assay? An analysis by ranking confirmed that it is most likely due to the differences inherent to the methods used and to the variability in the assay. See also answer 1) to reviewer#1, that addressed the same issue.

p4., line 38: There is no transition from the previous section (describing Fig. 2) to this section (describing Fig. 3). Please start this section by explaining the rationale for doing these experiments; otherwise it is difficult to follow.

We modified the text to introduce the rationale of doing RIP seq with Nanopore Direct RNA sequencing, now page 6 lane 1 to 6 :

"We conclude that Nanopore sequencing provides a robust quantification of Upf1-bound RNAs, comparable to the one obtained by short reads sequencing. The differences inherent to each method (ribodepletion, fragmentation and PCR in Illumina, dT10 priming in Nanopore) probably explain the observed discrepancies. However, considering that one mRNA result in one read with Nanopore technology, and that isoform complexity is also defined, this method depicted an accurate view of Upf1-associated mRNA, in addition to permit concomitant investigation of enrichment and poly(A) status, crucial for the next step of our study."

Fig. 3A: Since the y-axis is missing in this plot, it is not possible to compare the heights of the peaks and shoulders among the four samples.

We represented the data by bins of poly(A) length for this purpose.

Fig. 3, legend: The definitions for short, medium, and long poly(A) tails differ slightly between the figure legend and the Results section. Please use a consistent definition.

We modified the texts accordingly.

Fig 3D: It is unclear from the figure legend and the Results section (p. 5, line 41 and below) whether the indicated proportion of reads in each category refers to the total sequenced RNAs or only to those with short poly(A) tails. Please clarify.
It has been corrected in the legend and in the text (p7 lines 21-23)

Fig. 4A: The polysome gradient data as presented is difficult to assess and interpret. Instead of showing 25S and 18S rRNA levels, the authors should show the A260nm trace, as is common practice to show polysome data. This would allow judging the quality of the gradient. It looks suspicious that no 18S rRNA is present in fractions 7-10 (labeled as free 40S). Also the pattern of band intensities for HHT2 and rRNA looks strangly different between the fractions in the upf1Δ sample. Do the authors have an explanation for why there is much more HHT2 mRNA in fractions 19-20 and 25-26 than in the other fractions, and more 25S and 18S rRNA in fractions 15-16, 21-22, and 25-26 than in the other fractions?
Since this experiment did not bring essential results for the story, as suggested by reviewer #2 and #3 we removed it from the revised manuscript.

p.6, lines 22-25: Even though a reference is given, a brief description of the ePAT method would facilitate understanding of what is shown in Fig. 4B. Also, the abbreviation TVN is not explained.
We completed the description of the ePAT method (and TVN control) in the text (page 7 lines 38-47).
"To assess the poly(A) tail length of individual transcripts, we employed the extended Poly(A) Test (ePAT), a method allowing to visualize the diversity of poly(A) tail lengths [43]. We confirmed the efficient separation of mRNA species with different poly(A) tail lengths across the three fractions using ePAT on HHT2 (Fig. 5b, WT). ePAT uses a primer anchored to the end of the poly(A) tail to amplify a specific transcript by a PCR over the poly(A) tail [43]. ePAT signal is compared to a TVN signal, anchored at the beginning of the poly(A) tail with an oligoTVN primer marking the length of the amplicon without the poly(A) tail (A0). We confirmed the efficient separation of mRNA species with different poly(A) tail lengths across the three fractions using ePAT[43] on HHT2 (Fig. 5b, WT)."

Suppl. Fig. 4B: While the Upf1p enrichment values between the three reporter RNAs are very similar, the levels differ significantly. Do the authors have an explanation for this? Could it be that the no Stop and the Stop +1+2 constructs are less stable due to unintentionally inserted less optimal codons?
Yes, it is our interpretation as well. In order to remove all the stop codons within frame +1 and +2, we had to modify some of the codons and amino acids. These changes are the probable explanation to the observed destabilization of the mRNA at steady state compared to the original version.

Fig. 6C: Please discuss why the fraction of short poly(A)-tailed RNA is lower in the Upf1p-RIP samples of the upf2Δ and upf3Δ strains compared to the WT strain. Could this indicate that in the absence of Upf2p or Upf3p, Upf1p has a lower affinity for its RNA targets?
That is indeed our interpretation of the results. It is corroborated by MAplot representations that show that Upf1 losses its affinity for mRNA in the absence of upf2 or upf3.
In order to clarify this point, we integrated the MA plots in the revised manuscript (Supplementary Fig 5) and modified the text accordingly.

Fig. 6D: The Upf1p-RIP data on the two noPTC transcripts HHT2 and TDH3 show no enrichment of the short poly(A) tail fraction in the upf2Δ and upf3Δ strains, the majority of

these two Upf1p-associated mRNAs have a medium long poly(A) tail. In my view, that contradicts the conclusion that the NMD machinery has a critical role in the degradation of short poly(A)-tailed noPTC mRNAs (p. 8, lines 34/35). Rather, it suggests that when NMD is inactivated by depletion of Upf2 or Upf3, the Upf1p-assocaited mRNAs cannot be deadenylated to the same extent than in cells with active NMD. The authors cannot ignore this observation and should discuss possible interpretations and how this affects their proposed model.
We have a different interpretation of this observation. As illustrated by the MA plot showing the loss of affinity of Upf1 for short polyA tailed mRNA upon upf2 or upf3 deletion, the signal observed in Upf1 RIP is similar to the one of Input RNA when upf2 or upf3 are absent (Supplementary Fig. 5c, Supplementary Fig. 7c and Fig. 6c). The signal measured here is background corresponding to Input RNA. See also our answer to Point 5) of reviewer#1

Reviewer #2 (Remarks on code availability):

as stated above, this is not my area of expertise.

Reviewer #3 (Remarks to the Author):

Nonsense-mediated decay mainly targets short poly(A) tailed mRNAs lacking a premature termination codon

Gouhier et al.

Summary

In this work, short read Illumina sequencing of RNA associated with immunopurified yeast Upf1 (Upf1-RIP) revealed strong enrichment of Upf1 association with canonical NMD substrates – including PTC-containing mRNAs, Xrn1-dependent unstable transcripts (XUTs), and stable unannotated transcripts (SUTs). Additionally, a modest enrichment of transcripts lacking a PTCs (“noPTC”) was also identified. Notably, however, only a small subset of noPTC mRNAs (373 out of 2254, or 16 %) were found to be enriched in Upf1 binding. To better understand the class of noPTC mRNA bound to Upf1, Nanopore direct RNA sequencing was applied to provide information on both RNA poly(A) tail length and to distinguish isoforms of RNA expressed from individual genes. Based on this analysis, Upf1 was found to associate primarily with RNAs harboring short (0-25As) and medium (25-50As) poly(A) tails, but not RNAs with long (>51As) poly(A) tails, which, curiously, are a typical feature of PTC-containing transcripts undergoing targeting by NMD in yeast. Additionally, the majority of Upf1-associated transcripts identified by Nanopore sequencing lacked a PTC. The authors then showed that Hht2 mRNA, a representative noPTC mRNA enriched for Upf1 association, was polysome associated even when harboring a short poly(A) tail, and that in the absence of NMD (i.e. a upf1 deletion yeast strain), polysome-associated Hht2 mRNA had even shorter poly(A) tail lengths. The increase in abundance of Hht2 mRNA harboring short poly(A) tails in the absence of NMD was further confirmed using oligo-d(T) fractionation. Notably, the same trend (increased abundance of short poly(A) tailed transcripts in the absence of NMD) was observed for Tdh3 mRNA, which was not found associated with Upf1 in RIP assays. Using an

inducible, codon-optimized HIS3 reporter, the authors attempt to measure mRNA half-life for transcript populations with short, medium and long poly(A) tails, and suggest that in the absence of NMD, short poly(A) tailed His3 mRNA is stabilized. Finally, the authors provide additional Nanopore data indicating that Upf1 association with short poly(A) tailed transcripts is dependent upon Upf2 and Upf3, its cofactors in NMD.

Based on their data, the authors propose a model by which Upf1 is recruited to mRNAs harboring short poly(A) tails and targets them to NMD, which, if true, would represent a new class of NMD substrates. Their model relies heavily on the validity of the faux 3'UTR model (proposed in Amrani et al., 2004) which posits that Upf1 is recruited to terminating ribosomes when Pab1 is unable to promote efficient translation termination through its interaction with release factor eRF3 (due to the extended distance between Pab1 and ribosomes terminating at a PTC or, as proposed in this study, for short poly(A) tailed mRNAs that are predicted to lose Pab1 binding capacity).

While provocative, direct testing of the model is glaringly lacking and data that appears to directly contradict the model is ignored. Moreover, there is sufficient published literature that questions how Upf1 is recruited to mRNA, that Upf1 association with an mRNA does not a priori lead to targeting of a transcript to NMD (i.e. binding does not equal targeting), and the role Pab1 plays in NMD substrate recognition (see below), that undermine the faux 3'UTR model itself. Critically, the authors do not show a directly link between mRNAs harboring short poly(A) tails, loss of Pab1 from the transcript, inefficient translation termination, Upf1 recruitment, and decay of the transcript through NMD-mediated mRNA decapping. Curiously, they do not address why Upf1 is not found to bind to all mRNAs with short poly(A) transcripts (i.e. from all genes) or (on the flip side) why only 15% of noPTC mRNAs are bound by Upf1. One simple interpretation for these data is that Upf1 samples all transcripts, and that it disproportionally binds a subset of stable, highly abundant, deadenylated transcripts (lacking a PTC). The observation that short poly(A) tailed mRNAs accumulate and turn over more slowly in NMD mutants could simply be a consequence of a change in kinetics of mRNA decapping (the rate limiting step in decay in yeast) in wild-type cells versus cells with a completely different transcriptome due to inactivation of NMD. Indeed, in the absence of NMD, both direct and indirect NMD substrates would accumulate and compete for the decapping machinery and thus accumulate with short poly(A) tails.

General Comments:

1) In several instances, the data fails to support the proposed model.
    a) Upf1 shows similar levels of association (by RIP) with both short poly(A)-tailed transcripts and medium poly(A)-tailed transcripts (Figs. 3A, 3C). According to the authors, medium poly(A)-tailed transcripts should still harbor long enough tails to bind Pab1 and, thus, Upf1 would not be predicted to be recruited to those transcripts.
    We previously demonstrated that the fraction of the representative noPTC_enr HHT2 transcript associated to Pab1 has much longer poly(A) tails than those associated to Upf1 (see Figure EV2 D in Audebert et al, 2024), which are very short.
    In the Upf1-RIP, it is also expected to get a bit of background during the immunoprecipitation. Medium polyA tails are very abundant in the input (53% of all

transcripts), and thus might be also be present in the background signal. Contrarily, the short fraction represents 23% in the input, and increase to on average 44% in the RIP (with values comprised from 40 to 47% depending the replicate). The percentage difference between the WT Input and the Upf1-RIP sample is +20.6% for short, and -6.7% for medium or -14% for long, which means that Upf1 has a clear preference for short tailed mRNA. To conclude, the presence of some medium tailed transcripts in the Upf1 purification is not incompatible with our model.

b) Tdh3 mRNA, the representative noPTC, short poly(A) transcript that is not found to be bound by NMD also appears also to be stabilized in the absence of NMD. This suggest that the changes in abundance of these mRNAs in wild-type versus NMD mutants is independent of Upf1 association.
We demonstrated that the fraction of TDH3 that is associated to Upf1 has shortened poly(A) tails compared to input, which implies that the subfraction of mRNAs detected by Upf1 is specifically bound, otherwise it would show the same poly(A) profile than in the WT input. We think that there is a confusion between “not enriched” in Upf1-RIP and “not bound” by Upf1 (see also reviewer #1, answer 2). If we take in account the variability of the fractions of mRNA with different poly(A) tail length for a given gene, it is easy to see why for some transcripts, having relatively high levels of short poly(A), we can see an enrichment in the Upf1-associated fraction, while for others, no enrichment would be apparent, but binding of Upf1 can be demonstrated by analysing the increase in the proportion of short poly(A) transcripts in the purified fraction.

c) Figure 3C indicates that not all short poly(A)-tailed mRNAs are Upf1-bound/enriched, indicating that a short poly(A) tail is not the sole determinant of recruiting/binding Upf1.
It is entirely possible that the presence of a short poly(A) tail and binding of Upf1 are independent but correlated events. However, as explained above, enrichment alone is not a robust estimator of binding. The fact that Upf1 purified transcripts display a different poly(A) tail profile, with an increase in short poly(A) tail transcripts in the Immunoprecipitation strongly support the specificity of Upf1 for these RNA isoforms. A major message of the manuscript is that the use of poly(A) length allows to unravel a new and important class of Upf1-bound transcripts.

d) It is unclear why only 16% of no_PTC mRNA are bound by Upf1 and the other 84% are not. Are their features of this subset that might provide an alternative explain for why Upf1 enrichment on them?
We believe that the other 84% can contain a fraction that is associated with Upf1, even if enrichment is more complex to analyse. If the short-tailed subfraction is minor in the input, even when strongly present in the Upf1-RIP, it might not be seen as enriched (*TDH3* is such an example, shown in Fig 6d). Only poly(A) length measurement allows to estimate the real binding of Upf1 to short poly(A), when such mRNA represent a minor fraction of a given population of transcripts for a given gene.
We looked for additional features to explain the difference between noPTC and noPTC_enr. Interestingly, we found that noPTC_enr transcripts tend to have slightly longer 3’UTR and shorter coding sequences (see new Supplementary Fig1 d and e). Codon usage for the no_PTC_enrich mRNA population was similar with the codon

usage of no_PTC mRNAs not obviously enriched in the Upf1 bound fraction with the notable exception of the GGG and GGC glycine codons, which were over-represented in the no_PTC_enr. Both noPTC categories completely differed from the codon usage of PTC mRNAs (see also new Supplementary Fig 2). We amended the text with supplementary figures and a comment about this point (page 4 lines 8-22).

"We further analysed the features of noPTC and noPTC_enr mRNAs. Notably, despite no stabilization upon UPF1 deletion, noPTC_enr mRNAs harboured shorter coding sequences and slightly longer 3'UTRs (Supplementary Fig. 1d-e). Since codon usage correlates with mRNA abundance and stability[35], we examined the features of PTC, no_PTC_enr and no_PTC coding sequences. Relative codon usage, in comparison with all coding sequences from yeast, showed specific patterns (Supplementary Fig. 2a). Consistently, coding sequences for PTC containing transcripts were enriched in destabilizing codons, as estimated by the negative correlation between relative codon usage and the codon stability coefficient (Supplementary Fig. 2b). On the contrary, the noPTC population consisted mostly of relatively abundant mRNAs with a codon usage that was biased accordingly (Supplementary Fig. 2c). Finally, the noPTC_enr population showed a positive correlation between its codon usage and codon stability coefficients, with the notable exception of the GGG and GGC glycine codons, which were over-represented (Supplementary Fig. 2d). Altogether, codon usage for the no_PTC_enrich mRNA population was similar with the codon usage of no_PTC mRNAs not obviously enriched in the Upf1 bound fraction, and differed from the codon usage of PTC mRNAs."

2) The authors fail to reference published literature particularly relevant to this work, including that which identified a role for accelerated deadenylation in the degradation of NMD substrates (Cao and Parker, 2003; Mitchell and Tollervey, 2003), Upf1 binding to mRNA independent of translation (Hurt et al., 2013; Zund et al., 2013), and data contradicting a role for Pab1 in NMD (Roque et al., 2014; Meaux et al., 2008).

We agree with the reviewer that if previous work is directly related to the manuscript, it needs to be cited. We thus took a close and critical look at the results presented in the articles referenced by the reviewer and how they are related with the current manuscript and with other published work.

A) About the role of Upf1 in accelerating deadenylation and 3' to 5' degradation. We agree that, when decapping is affected, degradation from the 3' end of an NMD substrate migth be accelerated. The Cao & Parker 2003 Cell study used a deadenylation independent model to fit the degradation kinetics for several versions of PGK1-derived NMD substrates. The deadenylation-independent model was able to fit the experimental results, showing that deadenylation did not play a role in the degradation of NMD substrates. Moreover, a set of PTC containing *HIS4*-derived reporters displayed a very clear pattern of steady state poly(A) tail distribution: for the most unstable variant, the only visible species of mRNA had long poly(A) tails, while these tails showed broader distributions in more stable variants. These observations fit with the concept that mRNAs that are rapidly degraded do not have time to be deadenylated and present long poly(A) tails on average under steady-state. Since the RNAs were degraded rapidly, it was difficult for the authors to assess the speed of deadenylation, and they decided to decrease decapping efficiency by using a thermosensitive dcp1-2 strain at 30°C. Under these conditions, the four PGK1 mRNA containing PTCs displayed identical half-lives of about 20 minutes, in comparison with 40 minutes for the PGK1 without a PTC. Figure 5A of that paper presents the deadenylation profiles of the various constructs under these conditions. Examination of the published images seem to show no change in deadenylation rate up to 10 minutes of the kinetics, which, at the estimated rate of deadenylation of 2 .5A/min, would represent 25 As (almost half from the maximum length of about 60-70). This

simple visual inspection is at odds with the deadenylation rates of Table 2, which estimates deadenylation to 8-10 A/min for the NMD substrates. At these rates, Fig. 5A should have been quite different, with a size of poly(A) tail that was shortened by 25 As by 3 minutes, not 10 minutes. By the way, Figure 5B, showing the longest poly(A) tails detected along the chase period, also indicates rapid similar rates of decrease in the first 10 minutes for WT and PTC-containing PGK1. Thus, with the reserve that the quality of scanned images might not be ideal, the estimated high deadenylation rate in the paper for the measured NMD substrates might have been overestimated. The presence of heterogeneous populations of transcripts with various deadenylation rates might provide an alternative explanation for the observed results. To conclude, while a slightly faster deadenylation could affect NMD substrates in a Upf1-dependent manner, this effect was only detected when decapping was altered, under conditions that might also have altered Upf1 function. Affecting deadenylation directly, through depletion of deadenylation enzymes stabilizes some mRNA in yeast, but not NMD substrates (e.g. Audebert et al., EMBOJ 2024, Fig 4C). It is thus unclear to what extent deadenylation speed changes occur during NMD. Moreover, the focus of our manuscript is not deadenylation *per se*, but the specific binding of Upf1 to deadenylated mRNA species. It is for these reasons that we did not cite Cao and Parker, 2003, Mitchell & Tollervey 2003 or Takahashi et al 2003 articles. Moreover, please note that the Mitchel & Tollervey and Takahashi et al. papers do not present evidence for accelerated deadenylation of NMD substrates – only that, when the 5' to 3' degradation pathway is affected, Upf1 can facilitate the degradation of NMD substrates from 3' to 5'. The proposal that this facilitation would be mediated by an interaction between Upf1 and Ski7 was not confirmed in any subsequent studies to our knowledge. In contrast, the interactions between Ski7 and the cytoplasmic exosome have been confirmed both by biochemical experiments and functional analyses.

Since degradation of mRNA from the 3' to 5' when decapping is diminished can play a role for oligoadenylated noPTC substrates to which Upf1 binds, we included the following phrase in the discussion (page 10 Lines 42-45) :

« Additionally, the variable rates of 3' to 5' degradation across different mRNAs may contribute to the observed heterogeneity of the degradation profiles for any given RNA, although this aspect could not be assessed with DRS either. »

B) The paper by Meaux et al., Mol Cell 2008, shows that an mRNA lacking a poly(A) tail but having a PTC is more unstable than its equivalent mRNA without a PTC and that Upf1 is required for this instability. Thus, since there is no poly(A) tail, Pab1 is not required for NMD. Moreover, strains lacking Pab1 also showed the ability of NMD to work. In conclusion, that paper reinforces the hypothesis that Pab1 assists translation termination when the stop codon is close to the 3' end of the transcript and that, if the stop is further upstream, Pab1 can no longer play this role and this leads to triggering NMD. The paper by Roque et al (RNA 2015) analysed the interactions between eRF3 and Pab1, which could underlie a role of Pab1 in translation termination. The expected result would be that NMD substrates should not be affected in any way by disrupting an interaction between eRF3 and Pab1. Strains with truncated versions of eRF3 and Pab1 domains showed indeed that *RPL28*(*CYH2*) pre-mRNA levels were not affected even if the half-life of a PGK1pG non-NMD reporter was increased. Altogether, these studies reinforced the hypothesis that a stop codon can be detected as a PTC if far from Pab1.

The revised version of the manuscript include a reference to the Meaux et al. 2008 demonstration that NMD can be active even in cells that lack Pab1 and for reporters lacking a

poly(A) tail. Since absence of Pab1 or its positioning far away from the stop codon are equivalent molecular events, those findings are fully compatible with our results and model. It is now mentioned on page 11 line 29-31 :

« This is consistent with previous work demonstrating that NMD can be active even in cells lacking Pab1 and for reporters devoid of a poly(A) tail[49].”

C) In addition, we added in the CRAC result section a paragraph citing the Zund and Hurt results, although it is unclear to what extent the observed binding was specific or not. It is cited in the text page 5, lines 2-5 :

« This observation is consistent with previous studies in mammalian cells, where Upf1 was shown to bind broadly to mRNAs, with a marked enrichment in 3' UTRs presumably due to translation-dependent displacement from coding sequences by elongating ribosomes[23,24].”

3) It is unclear why PTC-containing mRNA harboring long poly(A) tails was not found in Upf1 RIPs by Nanopore sequencing. One explanation is that this population of transcripts is rapidly degraded by NMD and depleted from the population. If true, then it is unclear how short poly(A) tailed mRNAs that are purportedly targeted to NMD are readily found bound by Upf1 here.

This is a pertinent remark. PTC-containing mRNA are moderately enriched in Upf1-RIP by Nanopore sequencing (3% in the input and 6 % in the RIP so it is doubled, see also Maplot in new Supplementary Fig. 3b). It is indeed surprising that classical NMD substrates do not represent a higher fraction of mRNA bound by Upf1. However, as mentioned by referee #3, PTC containing transcripts are very unstable (and also poorly abundant) whereas the population of short-tailed NMD targets that we identify corresponded to relatively stable and abundant mRNA species (see also MAplot Figures 1b and Supplementary Fig 5). In addition, the PTC targets are probably not part of the same mRNPs as Lsm1 whereas short-poly(A) targets are likely to be (as illustrated by the RNAse-sensitive association of Upf1 and Lsm1, Dehecq *et al, EMBO J* 2018). Finally, PTC mRNA are expected to be poorly translated (see Heyer and Moore, Cell 2016), whereas noPTC and noPTC_enr are found in the heavy polysome fractions (previous Fig. 4) and are optimised for translation (Supplementary Fig. 2). Considering the difference in the nature of these targets and their related mRNPs, it is reasonable to believe that PTC- and short poly(A)- NMDs follow a different degradation kinetics.

4) The authors equate Upf1 binding of an mRNA with its targeting to NMD. There are numerous examples in the literature of Upf1 binding NMD in-sensitive mRNAs and also that Upf1 binding does not a priori lead to targeting the mRNA to NMD (Hogg and Goff, 2010). The cited paper shows a correlation between Upf1 binding to long 3' UTR regions and the sensitivity of the bound transcripts to NMD in HeLa cells. When sequences that induce stop codon readthrough were added to a reporter mRNA, the authors showed that the amount of bound Upf1 decreased when ribosomes could infrequently translate over the region downstream the stop codon (in which Upf1 binds). Moreover, later work from the Hogg group (e.g. Fritz et al., J Biol Chem 2020), showed that, for transcripts that are protected from NMD, PTBP1 contributes to removing Upf1 from 3' UTRs.

It is possible that, as suggested by the reviewer, RNA bound by Upf1 and not subjected to NMD, might exist in yeast. However, we did not pretend to identify all the possible targets of Upf1 in our study. We unravelled a new class of substrates for NMD and shown evidence of their degradation through a pathway comprising Upf1 and other NMD factors. We also evaluated that this new class of NMD target might be quantitatively important as it comprises a

substantial fraction of Upf1 targets identified by Nanopore sequencing (> 40% of all reads, see Figure 4c and 4d - when PTC totalizes only 6%). This was also corroborated by CRAC analysis (see new Fig. 2).

Specific Comments:

1. Is it unclear in Figure 2 if the Nanopore sequencing data includes data for full length mRNA or whether decay fragments are included. This is relevant as decay intermediates would be decapped and expected to have shorter poly(A) tails (skewing the data), and that data is compared to mRNA bound by eIF4G that, by default, is capped and full length. This needs to be clarified and only full-length transcripts reported in the analysis. This is a good point. We performed a new careful analysis of the results and detected only very subtle differences in the profiles (mostly an important drop in sequencing depth - see figure below). In addition, since the 5' transcription start site (TSS) coordinate is sometimes fuzzy (see Malabat *et al*, eLife 2015), we could not be sure that the coordinates correspond to real 5'TSS so we preferred keeping all the transcripts. Moreover, we performed additional experiments that evaluated, for specific transcripts, the impact of UPF1 deletion and DCP2 depletion on their cap status (and 5'P fragments levels). The results, presented in the new Fig. 7, indicate that, for specific transcripts, such as HHT2, RPL6B and TDH3, the capped fraction increases in the absence of Upf1, with a decrease in the levels of 5'P fraction, especially for HHT2. These results are consistent to binding of Upf1 to capped oligoadenylated transcripts and with an impact of Upf1 on triggering decapping of these transcripts.

**Ratio FL/all reads**	**WT**	**Upf1-RIP**
All transcripts	60%	47%
XUT/SUT	44%	40%
Pre-RPL22B	95%	88%
TDH3	58%	55%
HHT2	73%	54%

Comparison ALL vs fragments

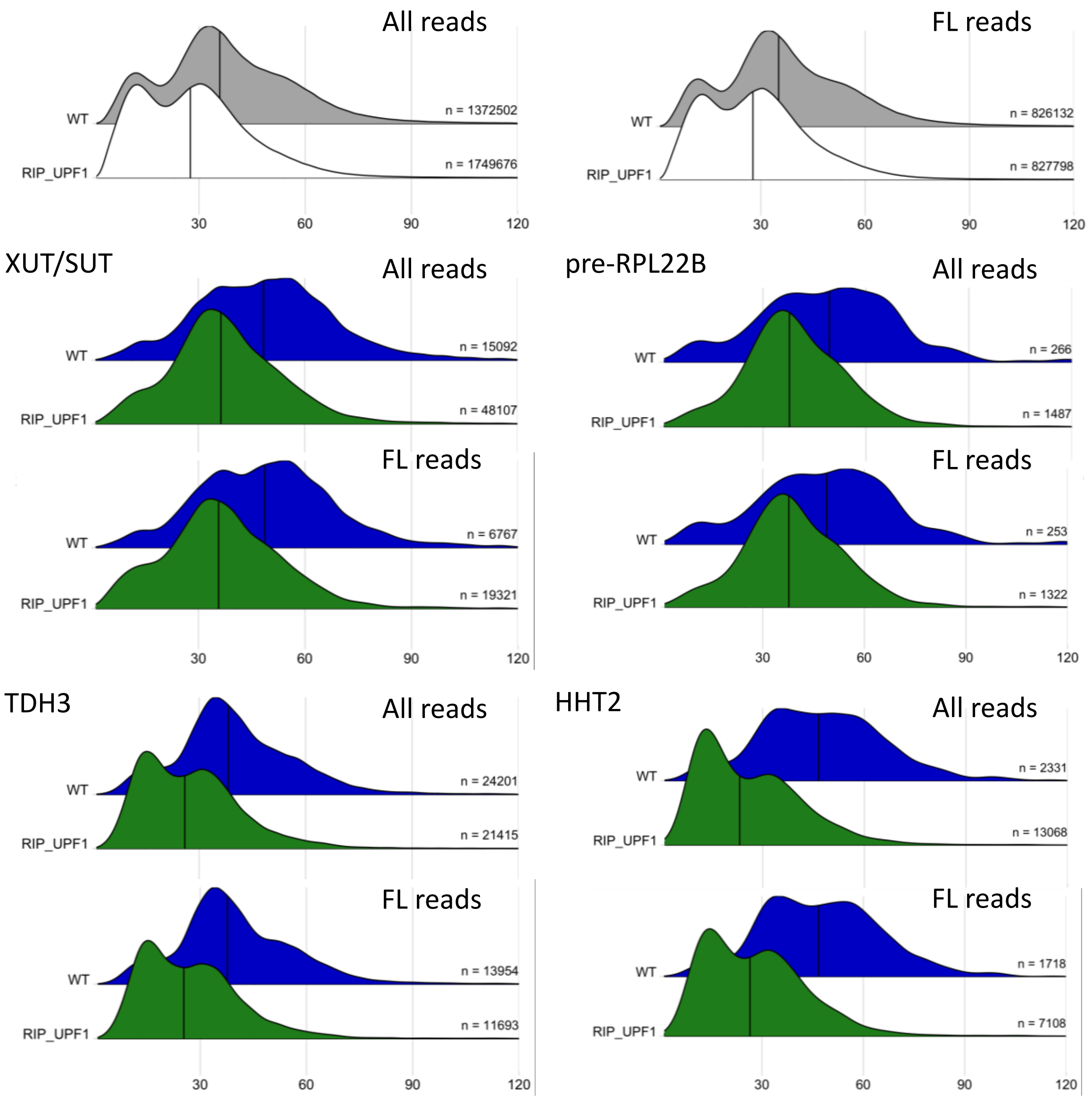

2. It is unclear why the Nanopore sequencing detects a greater proportion of Upf1-bound noPTC mRNA (some of which were not observed in Illumina-prepared sequencing libraries). Is it related to #1 above; the authors need to address.

This point was further explained in the text (page 5 lines 28-42) and in the Supplementary Fig 3. See also answer 1) to reviewer #1 :

"To further understand these results, we compared transcript ranks obtained from Nanopore and Illumina sequencing (rank 1 being the most abundant and 12296 the least abundant – Supplementary Fig. 3b). We noticed a strong positive correlation in the three scatterplots (with a Spearman correlation coefficient >0.8), indicating that despite variations inherent to Illumina and Nanopore technics, the relative estimated abundance of transcripts is comparable and consistent between the two methods (Supplementary Fig. 3b). However, the difference of rank showed a wider range in Upf1-RIP than in upf1Δ experiments (plot Rank WT – Rank Upf1 RIP compared to Rank WT – Rank upf1Δ Supplementary Fig. 3c and 3d), that look similar to foldchange comparison shown in Supplementary Fig. 3a."

3. It is unclear from the text where the transcripts per category in Fig. 1C come from and how the Upf1-bound PTC-containing dataset compares to those in the literature.

We gathered a list of 12695 transcripts including protein coding genes, pervasive RNAs (CUT, XUT, SUT), snoRNA, mitochondrial, Ty, ...
The list of **XUT/SUT** comes from Malabat *et al*, eLife 2015.
For protein coding genes, as mentioned in the text, we define the "PTC category including genuine **PTC**-containing protein coding mRNAs (He et al, Mol Cell 2003, Heyer and Moore Cell 2016) and mRNAs stabilized more than two-fold upon Upf1 deletion (this study)."
We refined the Heyer and Moore 2016 list by adding the information contained in Malabat et al eLife 2015, that characterized the NMD-sensitive 5' transcript isoforms transcriptome wide. "Accordingly, genes that express mRNAs without an obvious PTC were annotated **noPTC**, provided they express less than 10% of NMD-sensitive 5' transcript isoforms (in Malabat et al eLife 2015) and are not stabilized upon Upf1 deletion (in this study). Genes expressing more than 10% of NMD-sensitive 5' transcript isoforms were annotated as possible PTC (**PTC_pos**). The remaining transcripts (missing information) were considered to have an "**unknown**" NMD status".

Also, the numbers do not appear to match with the number of transcripts per category in Fig. 1D.
We answered this point in referee#2 additional points p.2, lines 35-37 and clarified in the text accordingly.

4. Fig. 3D does not report any information related to mRNA poly(A) tail length, yet the text makes reference specifically to short poly(A) tailed transcripts when this figure is discussed.
See also answer to referee#2 4) . It has been corrected in the legend and in the text (p7 lines 19-23) :
"The noPTC, noPTC-enr, PTC_pos and unknown categories, collectively accounted for 94% of the total reads in Upf1-RIP sample (Fig. 4d), among which 44% are short tailed, closely mirroring the overall distribution (Fig.4b and 4d). Short-tailed mRNAs thus represent more than 41% of all reads in the Upf1-RIP fraction while they constitute only 23% of all reads in the WT input."

5. The relevance for the inclusion of data on mRNA codon optimality (which influences translation elongation) and polysome association to support their model is unclear.

Additionally, optimal codon content is only marginally different between PTC and noPTC transcripts (Supp Fig. 1) and there is no discussion of differences between optimal codon content between Upf1-bound and unbound transcript pools.
We added a Supplementary Figure 2 with a more detailed analysis of codon optimality content across PTC and noPTC, enriched or not, categories. We found differences between these categories and amended the text accordingly (page 4 lines 10-22) :
"Since codon usage correlates with mRNA abundance and stability[35], we examined the features of PTC, no_PTC_enr and no_PTC coding sequences. Relative codon usage, in comparison with all coding sequences from yeast, showed specific patterns (Supplementary Fig. 2a). Consistently, coding sequences for PTC containing transcripts were enriched in destabilizing codons, as estimated by the negative correlation between relative codon usage and the codon stability coefficient (Supplementary Fig. 2b). On the contrary, the noPTC population consisted mostly of relatively abundant mRNAs with a codon usage that was biased accordingly (Supplementary Fig. 2c). Finally, the noPTC_enr population showed a positive correlation between its codon usage and codon stability coefficients, with the notable exception of the GGG and GGC glycine codons, which were over-represented (Supplementary Fig. 2d). Altogether, codon usage for the no_PTC_enrich mRNA population was similar with the codon usage of no_PTC mRNAs not obviously enriched in the Upf1 bound fraction, and differed from the codon usage of PTC mRNAs."

Finally, data in Fig. 4A are difficult to interpret due to lack of quantification and do not support the suggested role for Upf1 in the “redistribution of mRNPs containing short poly(A)-tailed mRNAs, suggesting a role of NMD in their remodeling or translation.”

As also suggested by reviewer #2, we removed Figure 4 from the revised version.

Point by point answer to referees
We thank the reviewers for their time, comments and questions, as they helped us to improve the quality of the manuscript. We addressed below the points raised by the reviewers that should answer all the major issues.

REVIEWER COMMENTS

Reviewer #1 (Remarks to the Author):

The manuscript by Gouhier et al. examines Upf1 targets, adding to the well-characterized PTC-containing transcripts, also non-PTC mRNAs bearing overly short pA-tails. The manuscript employs transcriptome prolifling using Illumina and nanopore sequencing on both total, and Upf1-immunoprecipitated RNAs. During revision the authors have put much effort in both improving the clarity of data presentation, and by adding novel data, including CRAC-profiling of Upf1 binding sites. Extending the role of Upf1 beyond decay of PTC transcripts into decay of normal mRNAs is a novelty, which will draw attention to the manuscript from the RNA community. The main hypothesis concerning Upf1 recruitment to those novel targets still remains speculative (specifically the Pab1 role). However, since this novel class of targets is so much better described following revision, it is clear to me that the manuscript itself will the basis of follow-up research. In conclusion, all my concerns were properly addressed, and I would encourage publication of the draft.
We removed the speculative model and replaced Figure 8 by a schematic representation of our results, that should clarify the notions of relative enrichment and global level changes when only a fraction of a given mRNA, the short poly(A) tail one, is bound and affected by Upf1/NMD.
The Figure 8 is now :

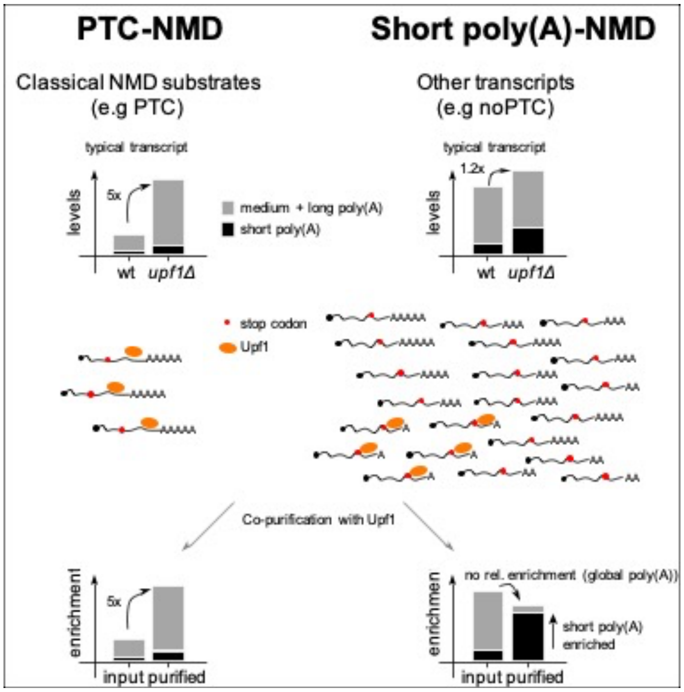

Reviewer #2 (Remarks to the Author):
Gouhier, Saveanu, and Badis present here a substantially revised version of their manuscripts. As was evident from the many points raised by all three reviewers, the original version was difficult to read and raised many questions, mainly regarding the interpretation of the presented data. Also, the long replies of the authors to the reviewers' points did not clarify the original concerns, at least not for me. In fact, on certain points, I got even more confused than before, and new questions arose. Hence, I had no choice but to evaluate the entire revised version again as if it were a new, original submission. Below are my comments:

Using a combination of RNA-seq and RIP-seq, the authors aimed at shedding light on the rules governing transcript recognition by Upf1 in yeast. While the majority of Upf1-associated RNAs were known NMD targets (XUTs/SUTs, and PTC-containing mRNAs), a minor fraction of the mRNAs with no obvious PTC (termed "noPTC") was also enriched in the Upf1 RIP-seq data (345; 16% of all noPTC transcripts). Given these numbers, the title of the manuscript is misleading, because it states that this minor fraction of transcripts represents the main targets of NMD, while the authors' data (Fig. 1) clearly show that the previously characterized PTC-containing transcripts (categories XUT/SUT and PTC) are much more abundant among the Upf1-associated RNAs. Furthermore, the enrichment values (Upf1-associated / Input) of transcripts belonging to this "noPTC_enr" transcript category are much lower than the corresponding values for classical PTC-containing transcripts (Fig 1e). While it is curious and worthwhile to investigate why some PTC-lacking transcripts are also enriched in the Upf1 RIP, the authors do not answer this question. The noPTC_enr transcripts appear to interact on average more with Upf1 in the 3´UTR than noPTC non-enriched transcripts, but the authors did not further analyse which features in these 3´UTRs cause enhanced binding of Upf1.
Instead, the authors analyzed by DRS the poly(A) tail length distribution of transcripts IPed with Upf1. The authors found that Upf1-associated NMD-sensitive transcripts had longer poly(A) tails than Upf1-associated NMD-insensitive transcripts, consistent with previous papers showing that NMD initiates degradation of yeast mRNAs by direct decapping without the need for prior deadenylation. However, on the specific example shown in Fig. 3 (RPL22B vs pre-RPL22B), this difference was quite small (33 vs 38 As). In general, Upf1-associated transcripts appear to exist in two overlapping populations: one population with short (<25; average 15) and one population with medium (average 30) long poly(A) tails (Fig 4a). But as already mentioned in my previous review, the representation of the data in Fig. 4a is problematic, because the y-axis is lacking, which prevents comparisons across the different sample: For example, when comparing upf1Δ with WT, it is unclear if the peak in the short poly(A) bin increased in upf1Δ, or if the peak in the medium poly(A) bin increased in WT, or if both changed in the absence of Upf1. Compared to the overall distribution of poly (A) tail lengths in the input of WT cells, transcripts with short poly(A) tails are substantially enriched in the Upf1 RIP sample. The authors show that, in particular, noPTC and noPTC_enr transcripts are 2 – 2.5-fold enriched in the Upf1 IP among the transcripts with short poly(A). Overall, these data indicate that Upf1 binds rather indiscriminately to PTC and noPTC transcripts (consistently with data from mammalian cells) and that a substantial proportion of the Upf1-bound noPTC transcripts has short poly(A) tails.
Applying ePAT on poly(A) length fractionated RNA samples showed that the levels of both, the noPTC HHT2 and the classical NMD target RPL28 pre-mRNA, increased in upf1Δ yeast, indicating

that also the short poly(A)-tailed noPTC transcripts are degraded in a Upf1-dependent manner (Fig. 5). This was confirmed by measuring the stability of a codon-optimized HIS3 reporter RNA (HIS3-opt). Until here, I think the data and interpretations are solid. Next, the authors conducted DRS and analyzed the poly(A)-tail length distribution in upf2Δ and upf3Δ strains (Fig. 6). If the proportion of Upf1-associated short poly(A)-tailed transcripts decrease in upf2Δ and upf3Δ strains, this would either suggest that in the absence of Upf2 and Upf3, they are more efficiently degraded, or that their deadenylation is slowed down and therefore a correspondingly larger fraction of these reads is now found in the medium and long poly(A) categories. Fig. 6d suggests the latter. Therefore, in my view, the data do not support the conclusion that Upf2 and Upf3 play a critical role in the degradation of short poly(A)-tailed noPTC mRNAs (lines 402-403). Rather, the data suggest to me that their absence prevents the fast deadenylation of the Upf1-associated transcripts.

Finally, in an elegant approach that allows the distinction between capped full-length RNAs and RNA degradation intermediates with a 5´ phosphate, the authors show that depletion of Upf1 increases the proportion of capped HHT2, RPL6B, and TDH3 transcripts and decreases the proportion of decay intermediates of these three genes, similar to inactivation of Dcp2. This indicates that Upf1-binding to these three mRNAs promotes their decapping.

My three main points that should be resolved before publication of this manuscript are the following:

1.) The strong focus on the categorization of the Upf1-immunoprecipitated RNAs, in particular the distinction between noPTC_enr and noPTC in the beginning of the manuscript, confuses and distracts from the main finding of the manuscript, namely that Upf1 is found associated with many mRNAs that are not NMD-sensitive after they have been almost completely deadenylated, and that Upf1 promotes decapping of these RNAs.
We modified the text (page 3 lines 37-46) in order to put the accent on this point: **Upf1 is found associated with many mRNAs that are not NMD-sensitive**, as requested.
The text is now: "*A reasonable assumption is that the mitochondrial signal reflects the background level of mRNA detection by Upf1. In these immunoprecipitations, almost all the transcripts identified in the Upf1 RIP experiment are above the mitochondrial background. We can thus conclude that Upf1 is found associated with many abundant mRNAs that are not NMD sensitive (**noPTC** categories, red and pink dots in Figure 1b). We distinguished 345 mRNAs from this category that are particularly enriched with Upf1 (log2FC RIP versus Input >1, **noPTC_enr**), whereas 1762 were not (**noPTC** – Fig. 1d). In addition, the **noPTC_enr** transcripts, were not stabilized upon Upf1 deletion, similar to other noPTC mRNAs (Supplementary Fig.1b). Considering the abundance of the noPTC transcripts, we counted that the non NMD-sensitive categories, composed of noPTC transcripts enriched or not in the RIP with Upf1, represented 56% (52%+4%) of all the reads in the Illumina sequencing dataset, while NMD-sensitive transcripts, composed of PTC and XUT/SUT represented only 2% (Figure 1c), although they were strongly enriched in Upf1 immunoprecipitates in comparison with their relative levels in the total extracts, as illustrated in Fig. 1e. Among the noPTC representative examples, some, such as HHT2 or RPS27A were enriched, though to lower levels than the PTC ones.*"

2.) A weakness remains that no explanation could be found for why some noPTC transcripts bind Upf1 better than others. In some assays, TDH3 has been included as a representative transcript of the category that is not enriched in the Upf1 RIP (yet still is bound by Upf1). It would be more informative if one would test transcriptome-wide for a correlation between Upf1-association and the effect on decapping of RNAs, rather than pick one or two specific transcripts of the different categories.

We identified differences between the noPTC and noPTC_enr categories. For example, the coding sequence and 3'UTR's lengths were different between these two subcategories (Supplementary Fig. 1d-e), with some differences as well in terms of specific codon usage (Supplementary Fig. 2a-d). The single transcript approach for testing cap vs 5'P ends levels that we developed for the first manuscript revision shows that Ufp1 promotes mRNA decay through decapping of noPTC transcripts. In addition, for the current revision, we validated the role of Upf2 and Upf3 in the same process.

Testing transcriptome-wide with the same approach for a correlation between Upf1-association and the effect on decapping of RNA, in particular for specific poly(A) fractions, would represent a whole project in itself, with extensive fine tuning to obtain robust results, in particular for the 5'P ends that are present in cells at very low levels. We agree that such a project would be of major interest for further investigation of the role of decapping and NMD in noPTC short poly(A) transcripts degradation.

As an alternative and to address the reviewer's question, we analysed data on mRNA levels changes following Dcp2 depletion from Chappleboim et al, NAR 2022. The levels of transcripts from the PTC and noPTC_enr categories were globally increased upon the inactivation of decapping while they were not affected by the inactivation of the deadenylation complex component Not1 (new Fig 7c). We added the sentence:

"To evaluate the sensitivity of the noPTC (enriched or not in Upf1-RIP) and PTC transcript categories to decapping or deadenylation, we assessed changes in transcript levels 60 minutes after Dcp2 or Not1 inactivation using a dataset from Chappleboim et al.[45]. We observed that transcripts from the PTC and noPTC_enriched categories increased the most upon decapping inactivation (dcp2), while remaining unaffected by the deadenylase complex inactivation (not1 - Fig. 7c)" page 10-11.

We added the Fig. 7c:

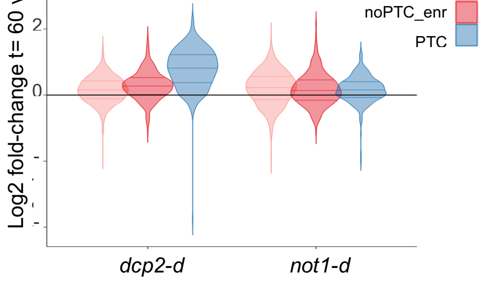

To better explain why TDH3 can serve as an endogenous reporter for short poly(A)-NMD effects, for example, we have also replaced the speculative mechanism model in Fig. 8 with a schematic representation of our observations from this study. This makes our findings easier to understand, illustrating how a transcript with lower relative abundance in the Upf1-purified fraction can still be specifically purified, enriched, and degraded when examining the short poly(A) fraction.

3.) In my opinion, the poly(A) tail profiles in Fig. 6 do not support the conclusion that Upf2 and Upf3, and hence NMD, are involved in promoting decapping of RNAs with short poly(A) tails. This would require additional evidence, for example, including upf2Δ and upf3Δ strains in the analysis shown in Fig. 7. With the currently provided data, the title of the manuscript is inappropriate and misleading. A more appropriate title would be "**Upf1 promotes decapping on short poly(A)-tailed mRNAs lacking a premature stop codon**". Without compelling data that the observed Upf1-promoted decapping of short poly(A)-tailed noPTC transcripts is also dependent on Upf2 and Upf3, the model proposed in Fig. 8 and the entire Discussion remain highly speculative.

To address this point, we added to Fig. 7 the results of decapping tests in *upf2Δ* and *upf3Δ* strains. These new results show that the absence of Upf2 and Upf3 confers a similar effect on the capped population of mRNA as the absence of Upf1 and enabled us to confirm the involvement of the whole NMD machinery in the process of "short poly(A)-NMD".

According to these new results, we modified the text (Page 10 and 11) as follow:

"In addition, the enrichment of PTC and noPTC_enr targets observed with Upf1-purified fraction in a WT context was reduced to 1 (no change) when Ufp2 or Upf3 was absent (Supplementary Fig. 4a and 4b). These results strongly suggest that Upf1's ability to bind short poly(A)-tailed noPTC transcripts could be altered in the absence of Upf2 or Upf3. As expected, the proportion of short-, medium- and long-tail species by categories in upf2Δ and upf3Δ strains were very similar to the ones observed in upf1Δ, but the proportions of mRNA immunoprecipitated with Upf1 in these conditions were importantly decreased (Supplementary Fig 4, 5c, and 8c).

…

Upf1 and cofactors Upf2 and Upf3 trigger the decapping of noPTC mRNAs

…

While our data suggest that the decapping defects demonstrated here are the direct consequence of Upf1 binding to short poly(A) mRNA, we cannot fully exclude the hypothesis that stabilized NMD substrates accumulating in the absence of Upf1 could, somehow, decrease decapping efficiency for other mRNAs.

…

Overall, our findings support the involvement of Upf1, Upf2 and Upf3 in promoting the degradation of noPTC mRNAs, and especially on noPTC_enr mRNAs, via stimulation of their decapping. These observations, combined with the observed loss of Upf1's ability to recognize both short poly(A) and canonical PTC-NMD targets in upf2Δ or upf3Δ strains, strongly suggest that short poly(A)-tailed RNA degradation is mediated through a mechanism involving the full NMD machinery."

The new Fig. 7b is now:

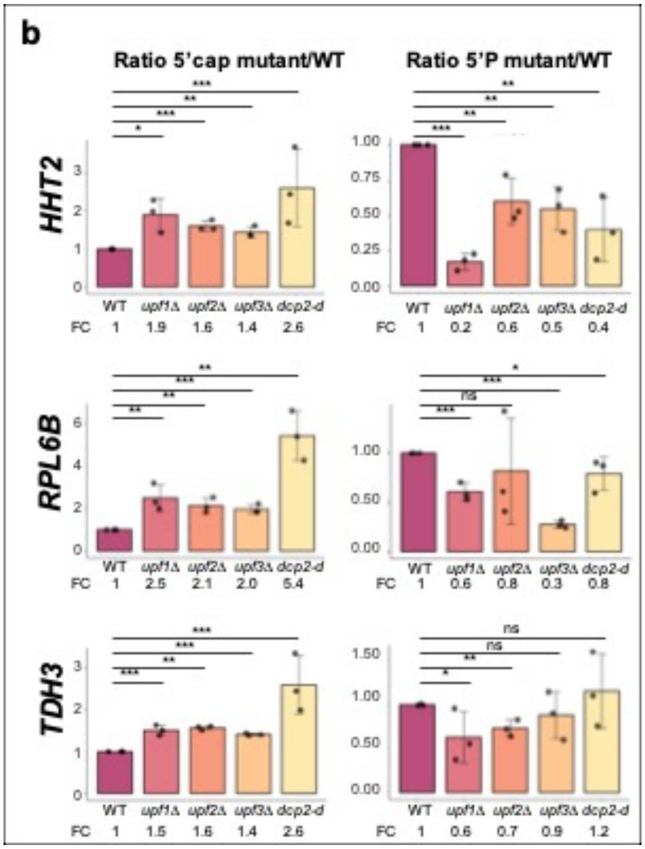

Reviewer #3 (Remarks to the Author):
The manuscript has improved from the first submission and the data indicating Upf1 binds mRNA lacking at PTC is convincing. However, this reviewer remains unconvinced that the data supports a conclusion that Upf1 is specifically recruited to short poly(A) tail mRNAs and that these transcripts are targeted to NMD.

1. The authors claim “a low immunoprecipitation background” (line 110) in the manuscript based on almost undetectable mitochondrial-encoded mRNAs in their Upf1-IP but suggest in their rebuttal that co-purification of Upf1 with medium poly(A) transcripts is due to background. Figure 4A and B indicates that Upf1 copurifies almost equal amounts of short and medium poly(A) tail transcripts, which, if due to background contamination, puts the entire analysis of Upf1-associated RNA in question. Notably, in Fig. 3, Upf1 is shown to bind RPL22B transcripts (lacking a PTC) with an average tail length of 33 nucleotides – which would place it in the ‘medium’ length poly(A) tail category (and which would be expected to be bound by Pab1).

Our RIP-seq Illumina results presented a very low background level (as validated by the very low mitochondrial transcripts levels). RIP-seq results from Nanopore experiments had potentially higher background levels, but had the advantage of being able to distinguish subpopulations of transcripts with different poly(A) tail lengths. This striking specificity, originally shown on individual transcripts in Suppl. Fig. 4a, was visualized by the relative increase in the fraction of short

poly(A) transcripts that we now represented in a new Supplementary Fig. 6 and copied below for convenience. It is clear that the increase in the fraction of short poly(A) tails observed with Upf1 and the relative decrease observed with the control eIF4G purification cannot be due to background. As requested by the reviewers and the editor, we now removed the speculative model originally shown in Fig. 8 about the involvement of Pab1 and replaced it with an illustration that clarifies how short poly(A) tail mRNA can be enriched in the transcripts associated with Upf1.
To answer the last part of the reviewer's question, about *RPL22B* transcripts results shown in Fig. 3. The manuscript text was probably unclear, as the pre-mRNA of *RPL22B* is definitely in the PTC category. We modified the text and figure annotations to better convey this. We do expect that poly(A) tail length have no major impact on the binding to Upf1 of PTC containing, NMD targeted transcripts.
The Supplementary Fig. 6 is now:

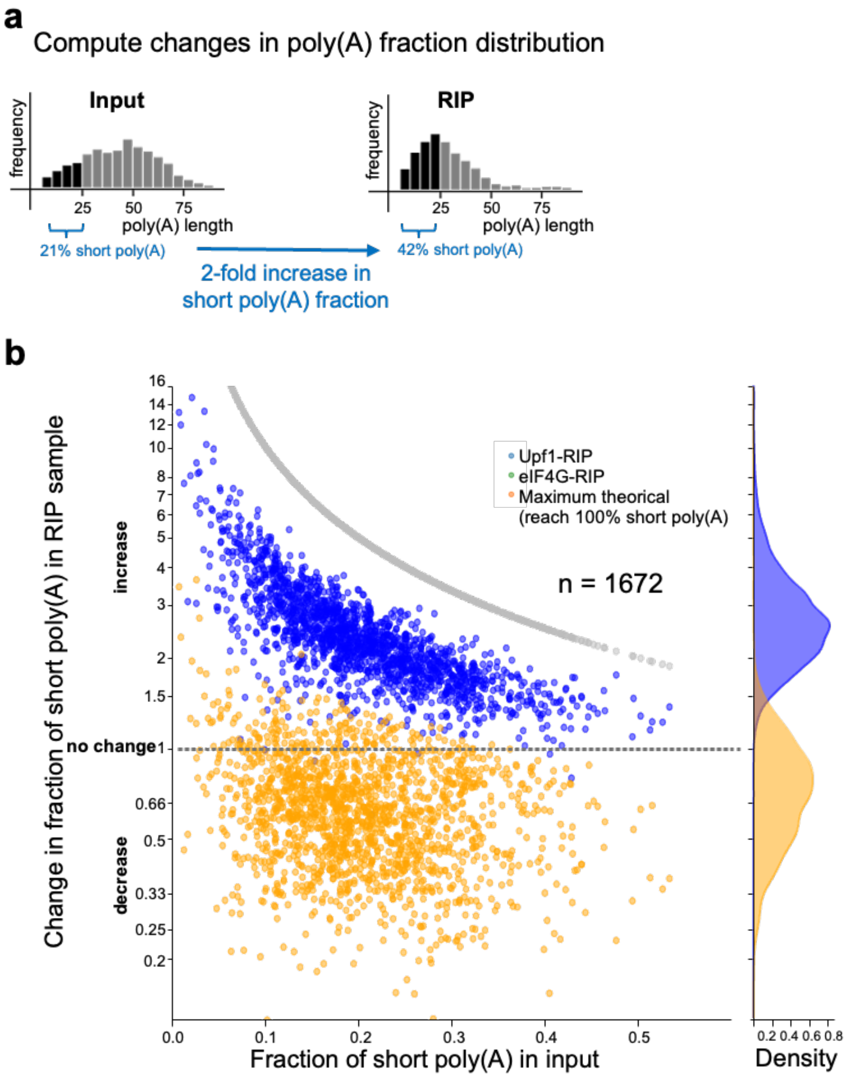

2. The model that NMD targets short poly(A) tailed transcripts rests on the assumption that Pab1 association is lost for transcripts with poly(A) tails shorter than 30 and that this leads to inefficient translation termination (and recruitment of the NMD machinery). Neither of these assumptions are tested here or experimentally supported. Although the authors draw attention to a previously published observation that short HHT2 mRNA is not co-purified with Pab1, this is insufficient to suggest that all short poly(A) transcripts are void of Pab1. Moreover, published biochemical and CLIP analyses of Pab1 suggests that it can efficiently bind A-rich sequences 12 nucleotides in length (although the protein covers 25-30 nucleotides).

Although supported by observation from Audebert et al. 2024 in the case of *HHT2*, we agree with the reviewer that the role of Pab1 in affecting the binding of short poly(A) mRNA to Upf1 is unclear and will need further investigation in the future. We tested during the previous revision period the tethering Pab1 to a reporter transcript to consolidate this point but the experiments proved to be technically challenging and more difficult than we initially thought. Since the model based on a role of Pab1 in the binding of Upf1 to short poly(A) transcripts is no longer presented in the current revision, we believe that the questions raised by the reviewer are interesting points to be addressed in future work.

3. The author's interpretation of Meaux et al (2008) is that NMD occurs in the absence of Pab1; however, a key observation is that a no-PTC transcript completely lacking a poly(A) tail (due to ribozyme cleavage) is not targeted to NMD, which would be expected if their model was correct.

Since the title of the Meaux et al., paper is "NMD in yeast does not require PAB1 or a poly(A) tail", our interpretation is in line with the original paper main message. We understand the point raised by the reviewer and we agree that it would be expected that a reporter lacking a poly(A) tail or Pab1 should be a substrate for NMD. We are not sure that the data reported in the Meaux et al. paper actually answer this question. One of the reporters, based on GFP, has an estimated 3' UTR region of 6 nucleotides and it is unlikely that it can bind the NMD machinery. The *HIS3*-based reporter has an estimated 3' UTR region of 55 nucleotides, but it is rapidly degraded by the 3' to 5' pathway. The Meaux et al paper could not thus analyse the impact of the absence of Upf1 alone on the reporter's half-life. The reported results only involve double mutants of ski7Δ/upf1Δ. Thus, we do not believe the Meaux et al data definitely answers the question of whether short or no poly(A) mRNA are targeted for degradation. Our results, obtained by fractionation of transcripts according to their poly(A) tail, either with reporter mRNA or with endogenous ones however, indicate their stabilization in the absence of functional NMD.

4. The proposed model (Fig. 8) indicates that Upf1 (and the other NMD factors) is recruited to the terminating ribosome of short poly(A) tailed transcripts (via interaction with eRF1), however, their own CRAC data (Fig. 2) maps Upf1 along the 3' UTR of these transcripts; it is unclear how inefficient termination can lead to 3' UTR binding by Upf1 – this needs to be reconciled.
Upf1 recruitment to RNA is an open question in the NMD field that is not directly addressed in our manuscript. Once bound to an RNA, Upf1 is an ATP-dependent RNA helicase that can advance from 5' to 3' on relatively long distances, as demonstrated by single molecule experiments (for example, Kanaan et al., Nat Commun 2018, PMID: 30218034). A recruitment downstream the

stop codon does not imply that Upf1 and its associated factors cannot move along the 3' UTR. We agree with the reviewer that whether and how translation termination defects triggers Upf1 binding to the downstream untranslated region is a major open question in the field. We removed the model and replaced it with a schematic representation of the results (Fig. 8).

5. The authors should assay the unbound fraction to determine the abundance of short poly(A) tailed mRNAs that fail to associate with Upf1 and explain how these transcripts escape recruitment of Upf1 and targeting to NMD under the current model.

This kind of experiments are challenging because it would need to be finely calibrated in order to ensure depleting all the Upf1 proteins from the lysate and it is outside the perimeter of our study. At this stage of revision and in regard of the amount of work that the proposed experiments would require, we do not believe that the request is reasonable. Moreover, as in any other molecular system at equilibrium, we expect a distribution of short poly(A) mRNA in the Upf1 bound and in Upf1 free fractions.

6. The authors cannot rule out that inhibition of decapping (and disappearance) of short poly(A) mRNA in strains lacking NMD activity is indirect. Indeed, in upf deletion strains, prior NMD substrates are now stable, abundant within the transcriptome, and are substrates for deadenylation-dependent decapping. The flood of additional substrates for normal mRNA turnover would be expected to lead to slower decapping rates (of deadenylated transcripts), independent of a direct effect by NMD.

By definition, changes in the levels of various RNA species in a strain lacking a factor can be direct or indirect. We can imagine, as the reviewer mentioned, various scenarios by which the observed increase in the levels of the short poly(A) mRNAs in the absence of Upf1 can occur. But we prefer to use the simplest explanation that is backed up by biochemical evidence presented in this manuscript and solid biochemical and functional results published in the past on the mechanisms and interactions between yeast Upf1 and decapping. Binding of a protein, in this case Upf1, to a short poly(A) mRNA together with the proven ability of Upf1 to recruit Dcp2 and its cofactors and to activate decapping on NMD substrates, strongly suggest that the effects we see are direct. We now added a cautionary phrase in the text in the Discussion section:

"While our data suggest that the decapping defects demonstrated here are the direct consequence of Upf1 binding to short poly(A) mRNA, we cannot fully exclude the hypothesis that stabilized NMD substrates accumulating in the absence of Upf1 could, somehow, decrease decapping efficiency for others mRNAs."

7. Literature supports a model that Upf1 samples all transcripts and binds mRNA independent of translation. Under this model, Upf1 would be expected to associate disproportionally with stable, highly abundant transcripts that lack PTCs, and transcripts with longer 3' UTRs – which is consistent with the presented data. Curiously, PTC-containing transcripts with long poly(A) tails do not copurify with Upf1 (Fig. 4B) – despite being the canonical class of NMD substrates. A likely explanation for this is that these transcripts are rapidly degraded and devoid in the sample; however, if Upf1 also targets short poly(A) transcripts to rapid decay by NMD, why are they present in the sample and copurified with Upf1?

PTC-containing transcripts with long poly(A) tails **do** copurify with Upf1, but they are so poorly represented (6%) of all reads compared to other categories much more abundant, that they are

not visible in the global picture Fig4b. Insofar, looking at the most representative population of PTC-NMD target, the XUT_SUT category of transcripts, we can observe poly(A) tails longer than 30A in the Upf1 RIP experiment (Supplementary Fig 4).
The reviewer's interpretation of the data presented in Fig. 4b, that PTC-containing NMD substrates would not be bound by Upf1 is incorrect. We actually see a high enrichment of PTC containing long poly(A) tail mRNA with Upf1, although in this case the poly(A) status does not seem to affect binding to Upf1, as expected.

8. There are several concerns around the RNA-Seq analysis:
a. The 'moderate' correlation of Upf1-associated transcripts identified by Illumina versus ONT sequencing is concerning (Fig. S3; R=0.48). Despite inherent differences in this approach, I would not expect this low of agreement on the transcripts that copurify with Upf1.
We already addressed this point in the first revision and have shown that the transcript ranks are well correlated between Illumina and Nanopore results. We concluded that these differences are inherent to the methods used and variability of background in experiments.

b. Ribosomal proteins represent a large portion of the genes in the noPTC category of transcripts crosslinked to Upf1 (CRAC data Fig.2) and many are known to contain introns. How are the authors able to distinguish Upf1 binding to unspliced mRNA, which would be expected to be targeted to NMD. Was Upf1 found to CRAC to intron-containing sequences for these transcripts?
We observed Upf1-CRAC signal near the stop codon in the retained intron of some of the RP pre-mRNAs, especially in those prone to intron retention. However, in term of number of molecules, the majority of the mRNA measurable for all the RP genes corresponded to mature mRNA, which was distinguishable and quantifiable in Nanopore sequencing data and confirmed by Northern blot.

c. Inclusion of partially degraded transcripts in the ONT data is misleading.
We addressed this point in the first revision, showing that the overall profile (as well as the profile of individual transcripts) was similar to the Full-length transcripts alone, but with a substantially greater depth, to justify maintaining the data analysis for all of them. This is now mentioned explicitly in the manuscript:

"A similar distribution of poly(A) tail length was observed for full-length RNA molecules, defined by the presence of the signal all along the transcript. Considering only these molecules for further analysis would strongly reduce the depth and the number of analysed genes."

We thank the reviewers for the time allowed to explain their remaining concerns about the revised manuscript.

REVIEWERS' COMMENTS

Reviewer #2 (Remarks to the Author):

With this second revision, the authors have addressed my remaining concerns. By replacing the highly speculative model figure with a data-based summary illustration, the manuscript is now scientifically more robust and focused on presenting the many interesting results rather than emphasizing the speculative interpretation.
I have no additional comments and judge this manuscript ready for publication.

Reviewer #3 (Remarks to the Author):

The main issue with this work is not whether UPF1 associates with mRNA in yeast lacking a PTC or having a short polyA tail – this data is sufficiently convincing (although the authors have still not addressed the specificity of UPF1 for only a subset of mRNA within this category). What remains unclear is whether UPF1 association is meaningful to the turnover of bound transcripts and whether these mRNAs are, in fact, targeted to NMD – this remains unclear and not fully supported by the data.

For the first comment, about not having addressed the specificity of Upf1 for a subset of mRNA, the manuscript, including the new Fig. 8, answers the previous questions. The presented results demonstrate the specific affinity of Upf1 for short poly(A) tail mRNA. We now explain how global enrichment cannot be used as a metric to understand binding of Upf1 to different mRNA species. Altogether, the question of specificity is clearly addressed by showing that short poly(A) mRNAs are enriched with Upf1 for all transcripts for which we obtained reliable measurements.
To summarise our findings in relation with the second part of the reviewer's concerns, in our study we have shown that:

1. Upf1 is strongly associated to noPTC mRNA by RIP (Fig1) and CRAC (Fig2) experiments, and preferentially to short-poly(A)-tailed mRNA (RIP-seq associated to Nanopore sequencing Fig4).
2. That the short-poly(A)-tailed fraction is increased (Fig4b and c), stabilized (Fig 6a) and even less decapped (Fig7b) in the absence of Upf1
3. That this specific recognition (Fig6) and decapping (Fig.7) require the other NMD factors Upf2 and Upf3

The results are consistent to qualify short-poly(A)-tailed mRNA as a new category of NMD machinery targets.

In the literature, a plethora of articles defines NMD targets as mRNA stabilized more than two-fold upon Upf1 inactivation (Celik et al, 2017; Heyer & Moore, 2016; Alalam et al, 2022; Johansson et al, 2007; Malabat et al, 2015, ...).

Here we have provided robust evidence that the short-poly(A)-tailed subpopulation of mRNA are stabilized in the absence of Upf1, Upf2 and Upf3, thus fiting the commonly accepted definition of NMD targets. The only difference with canonical NMD targets is that the

stabilization is distinguishable only after fractionation and isolation of the short-tailed subpopulation.

It is now admitted by the authors that the data does not distinguish between a direct or indirect role for NMD in the turnover of short polyA-tailed mRNA, and so it can not be concluded that NMD TARGETS these transcripts.
This comment is not based on what is actually written in the manuscript. Here is the part to which the reviewer probably refers to:

"While our data suggest that the decapping defects demonstrated here are the direct consequence of Upf1 binding to short poly(A) mRNA, we cannot fully exclude the hypothesis that stabilized NMD substrates accumulating in the absence of Upf1 could, somehow, decrease decapping efficiency for other mRNAs."

Thus, we do not state the belief that our results are not linked with NMD. As scientists, we express doubts, although, also as scientists, we prefer the simplest explanation to the observed results. This explanation is that short poly(A) tail mRNAs are bound by Upf1, which assists their degradation through its well characterized interaction with the decapping machinery. Please note that the same sceptical view can be expressed for canonical NMD - one could say that maybe the effects of an absence of Upf1 on decapping of NMD substrates are indirect. One day, when an NMD process will be reconstituted *in vitro*, the direct or indirect action of decapping will be definitely established. Until then, the lines of evidence we provided and a large corpus of published results suggest that the impact of Upf1 on mRNA degradation goes directly through Dcp2 recruitment and decapping activation.

To recapitulate, what we show in this manuscript is that Upf1 binds to short poly(A) mRNA (Nanopore results, Fig. 4) and that this binding is likely to be direct since cross-linking with Upf1 occurs for “non-NMD” noPTC transcripts (Fig. 2). The interactions of Upf1 with the decapping machinery are well established (please refer to our recent review summarizing the published results about this, Ruiz-Gutierrez et al., J Mol Biol 2026). Finally, we have shown that for several noPTC mRNA, the absence of Upf1 or the NMD cofactors Upf2 and Upf3 leads to a decrease in the decapped mRNA fraction. All these findings support that Upf1, helped by other NMD cofactors most likely targets noPTC short-poly(A) mRNA for degradation.

It is noted that data supporting a mechanism for UPF1 recruitment to short-tailed mRNAs is scant (and does not account for why only some of these mRNAs are targets). Indeed, the authors have removed their mechanistic model due to lack of direct evidence or contradictory findings (Fig. 8) but still invoke a role for PAB in the Discussion. The replacement for Fig. 8 represents a Graphical Abstract (of ‘expected results’) and is not helpful to the reader.

We would like to point out the fact that even the mechanism that recruit Upf1 to NMD targets is still not fully understood and is still a subject of debate in the field. Thus, it seems unfair to ask, when describing an entirely new role for the NMD machinery, for the detailed molecular mechanisms of Upf1 recruitment in this new process. In the latest revision, we invoke the possibility of a role of Pab1in the induction of this process. Since the short-poly(A)-tailed mRNAs targeted by Upf1/2/3 have too short poly(A) tails (<25A) to be efficiently associated to Pab1 it is reasonable to speculate that Pab1 is absent on these mRNPs, and that the absence of Pab1 on these mRNA could provoke the same problem on the termination of translation than when Pab1 is too far from the stop codon (in the case of the long 3’UTR of canonical PTC-NMD targets).

The text in the discussion is now:

"Similarly to the PTC-NMD where the long distance between Pab1 and the stop codon dictates mRNA recognition by the NMD machinery, we speculate that the absence of Pab1 could also triggers NMD in a way that we propose to name "short poly(A)-NMD". In both scenarios, translation termination could be impaired due to the absence of Pab1's stimulating effect on the translation termination factor eRF3[51] and Upf1, Upf2 and Upf3 could be required to solve the same translation termination defect, both by stimulating ribosome recycling and by recruiting the decapping complex for RNA degradation"

Finally, Reviewer #2 found the Figure 8 useful as a data-based summary illustration and we think it is important to help the reader to appreciate the complexity of Upf1 targets and the impact of their heterogeneity in computing enrichment factors from co-purification or mutant strain analyses.
